# Chronic hypoxia is associated with transcriptomic reprogramming and increased genomic instability in cancer cells

**DOI:** 10.3389/fcell.2023.1095419

**Published:** 2023-03-09

**Authors:** Raefa Abou Khouzam, Mohak Sharda, Shyama Prasad Rao, Stephanie Maame Kyerewah-Kersi, Nagwa Ahmed Zeinelabdin, Ayda Shah Mahmood, Husam Nawafleh, Munazza Samar Khan, Goutham Hassan Venkatesh, Salem Chouaib

**Affiliations:** ^1^ Thumbay Research Institute for Precision Medicine, Gulf Medical University, Ajman, United Arab Emirates; ^2^ National Centre for Biological Sciences, Tata Institute of Fundamental Research, Bangalore, Karnataka, India; ^3^ School of Life Science, The University of Trans-Disciplinary Health Sciences & Technology (TDU), Bangalore, India; ^4^ Center for Bioinformatics, NITTE deemed to be University, Mangaluru, India; ^5^ INSERM UMR 1186, Integrative Tumor Immunology and Cancer Immunotherapy, Gustave Roussy, EPHE, Faculty De médecine University Paris-Sud, University Paris-Saclay, Villejuif, France

**Keywords:** hypoxia, tumor mutational burden, solid tumors, transcriptome remodeling, genomic instability, chronic hypoxia, copy number variation

## Abstract

Hypoxia afflicts the microenvironment of solid tumors fueling malignancy. We investigated the impact of long hypoxia exposure on transcriptional remodeling, tumor mutational burden (TMB), and genomic instability of cancer cells that were grouped based on their inherent sensitivity or resistance to hypoxia. A hypoxia score was used as a metric to distinguish between the most hypoxia-sensitive (hypoxia high (HH)), and most resistant (hypoxia low (HL)) cancer cells. By applying whole exome sequencing and microarray analysis, we showed that the HH group was indeed more sensitive to hypoxia, having significantly higher TMB (*p* = 0.03) and copy number losses (*p* = 0.03), as well as a trend of higher transcriptional response. Globally cells adapted by decreasing expression of genes involved in metabolism, proliferation, and protein maturation, and increasing alternative splicing. They accumulated mutations, especially frameshift insertions, and harbored increased copy number alterations, indicating increased genomic instability. Cells showing highest TMB simultaneously experienced a significant downregulation of DNA replication and repair and chromosomal maintenance pathways. A sixteen-gene common response to chronic hypoxia was put forth, including genes regulating angiogenesis and proliferation. Our findings show that chronic hypoxia enables survival of tumor cells by metabolic reprogramming, modulating proliferation, and increasing genomic instability. They additionally highlight key adaptive pathways that can potentially be targeted to prevent cancer cells residing in chronically hypoxic tumor areas from thriving.

## 1 Introduction

Hypoxia is common to solid tumors manifesting upon the drop in oxygen below physiological levels. Moving away from the functional vasculature within the growing tumor mass, different levels of oxygenation levels exist creating sites of intermittent, or cyclic, hypoxia experiencing fluctuations in perfusion leading to episodes of hypoxia and reoxygenation; sites of acute hypoxia due to transient disruption of perfusion lasting minutes to hours; and areas of chronic hypoxia where there is continuously limited oxygen diffusion (Vaupel and Mayer, 2014; Bader et al., 2020). Hypoxia is known to contribute to tumor malignancy by promoting metabolic derangement, genomic instability, angiogenesis, invasion and metastasis, immune suppression, as well as treatment resistance (Vaupel and Mayer, 2014; Schito and Semenza, 2016; Abou Khouzam et al., 2020a; Abou Khouzam et al., 2020b; Abou Khouzam et al., 2022). One key player orchestrating these processes is the transcription factor hypoxia-inducible factor (HIF)-1α, which is only stabilized at low oxygen levels (Schito and Semenza, 2016). Hypoxia has also been implicated in aberrant splicing, reportedly inducing thousands of alternative splicing events, and affecting protein expression of both HIF-activated and independent genes (Sena et al., 2014; Brady et al., 2017; Han et al., 2017). Genes regulated by HIF and hypoxia have been used to generate hypoxia gene signatures acting as proxy indicators of a tumor’s hypoxic state and have associated hypoxia with worse prognosis in multiple solid tumors (Abou Khouzam et al., 2020a; Abou Khouzam et al., 2020b; Abou Khouzam et al., 2022). We have recently derived such a signature starting from fourteen cell lines representing six different tumor types (Abou Khouzam et al., 2021). The signature, constructed of eight hypoxia-activated genes was found to be associated with worse prognosis in pancreatic cancer. Furthermore, tumors that were classified as more hypoxic based on our signature displayed higher immunosuppression. In addition, we could report a higher number of mutations in more hypoxic tumors, and higher microsatellite instability (MSI), underlining the significance of hypoxia’s contribution to genomic instability (Abou Khouzam et al., 2021).

Genomic integrity is maintained through tight control and coordination between cell cycle progression and error-free DNA-damage repair. Failure of these systems results in genomic instability, a universal cancer hallmark, in which there is increased propensity of accumulating sequence mutations, MSI, as well as chromosomal instability. The latter entails structural changes through copy number gains, losses and translocations, or numerical changes, such as aneuploidy, and polyploidy (Kaplan and Glazer, 2020; Tang et al., 2021). Thus far, patient studies reporting on association between hypoxia and genomic integrity have done so primarily using hypoxia gene signatures. Bhandari and co-workers have published two such studies, examining 19 tumor types from The Cancer Genome Atlas (TCGA) in one and 27 tumor types from the Pan-Cancer Analysis of Whole Genomes (PCAWG) Consortium (Bhandari et al., 2019; Bhandari et al., 2020). They reported on positive associations between hypoxia and tumor mutational load and chromosomal instability. Looking at single base substitution (SBS) signatures, more hypoxic tumors experienced signatures with etiology of defective homologous recombination-based repair and DNA mismatch repair (MMR) (Bhandari et al., 2020). *In vitro* investigations have shown that hypoxia could lead to genomic instability through its downregulation of DNA repair genes and their respective pathways of MMR (Lu et al., 2014; Cowman et al., 2021), homologous recombination repair (HRR) (Bindra et al., 2005; Fanale et al., 2013), base excision repair (BER) (Chan et al., 2014; Farias et al., 2018) and Fanconi anemia (Scanlon and Glazer, 2014) (reviewed in (Luoto et al., 2013; Begg and Tavassoli, 2020)). Hypoxia has additionally been implicated in the repression of DNA replication genes and increased replication stress through stalled replication forks and decreased new origin firing that is time and oxygen dependent (Pires et al., 2010; Ng et al., 2018) (reviewed in (Luoto et al., 2013; Begg and Tavassoli, 2020)). In a study on breast cancer cells, we have shown that both intermittent and chronic hypoxia downregulate genes involved in DNA replication and repair. An increase in mutational load could also be reported upon exposure of cells to either condition, with frameshift deletions or insertions being the most common lesion (Hassan Venkatesh et al., 2020). How prolonged exposure to hypoxia could affect these processes, however, remains to be investigated. Furthermore, whether hypoxia can cause genomic alterations in the form of copy number alterations across the exome has not been tackled *in vitro*.

Increased genetic instability could mean an increase in novel non-self-peptides that could be processed and presented to the surveilling immune cells as neoantigens, thus eliciting a cytotoxic immune response. In support of this notion, MMR inactivation in mouse colorectal cancer (CRC) cells not only resulted in increased TMB, but also dynamic mutational profiles that led to neoantigens renewal *in vitro* and *in vivo*; an effect that was not observed in cells proficient for MMR (Germano et al., 2017). This increased TMB and clonal production of dynamic neoantigens was thought to explain the increased immune detection of MMR inactivated tumors and their resulting growth impairment in immune-competent mice (Germano et al., 2017). A longitudinal *in vitro* study on CRC cell lines with different molecular backgrounds reported that cells carrying mutations in DNA repair genes presented with a dynamic, fluctuating neoantigen load (Rospo et al., 2019). Xenografts using such cells, as well as patient derived xenografts carrying DNA repair lesions, displayed high TMB and neoantigen evolvability that was associated with decreased or even inactivation of antigen-presenting functions (Rospo et al., 2019). We have shown that hypoxia can contribute to clonal neoantigen production in breast cancer cells, where there was an increased neoantigen load in cells cultured under intermittent and chronic hypoxia compared to those cultured in normoxia (Hassan Venkatesh et al., 2020). While the cancer mutanome could produce mutated proteins, the cell requires functional machinery to process them to mutant peptides, as well as the presence of Major Histocompatibility Complex (MHC) molecules to present the neoantigens to circulating immune cells. Immune cells also need to be surveilling in the vicinity to become activated and elicit an immune response (Schreiber et al., 2011). In that respect, hypoxia has been shown to negatively impact the immunogenicity of tumors, with mounting evidence implicating this condition in promoting an immunosuppressive TME (Baginska et al., 2013; Berchem et al., 2016; Watson et al., 2021; Ziani et al., 2021).

Thus far, the effect of hypoxia has been investigated *in vitro* by applying different oxygenation levels and exposure times, but a comprehensive study on the prolonged exposure of tumor cells to hypoxia is yet to be conducted. This is highly relevant, since the actual time tumor cells can remain in a hypoxic state within the tumor can range from hours to even weeks (Vaupel and Mayer, 2014). Another missed consideration is that while hypoxia exists in 60% of tumors, it varies among tumor types, similarly, the *in vitro* response to hypoxia of cancer cells derived from these tumors could additionally vary. The degree of this inherent variability is yet to be measured, and whether it is correlated with the overall cellular adaptation to hypoxia has not been investigated to date. In this work we set out to address these open questions by ranking the hypoxic response of tumor cells based on our previously established hypoxia gene signature (Abou Khouzam et al., 2021). Then by focusing on the cells that are most sensitive in their response to hypoxia and those that are least sensitive, we examined the effect of long passaging in hypoxia on the genetic and transcriptional landscapes, including alternative splicing.

## 2 Materials and methods

### 2.1 Hypoxia scoring of cancer cell lines

Fold change (FC) values for the eight signature genes corresponding to three interdependent experiments carried out in duplicate were obtained from our previously published work (Abou Khouzam et al., 2020a). There were 12 cell lines included in total, representing cancers of the breast, cervix, colon, lung, ovaries, and pancreas. Two cell lines were scored per tumor type, and this was done based on the cells’ FC. First FC values for each gene per cell line were averaged and subsequently the geometric mean of all 8 genes per cell line was calculated. This value represented the cell line’s hypoxia score. Relative hypoxia scores were then obtained to compare the cells to each other by subtracting their hypoxia score from the geometric mean of all scores.

### 2.2 Hypoxia scoring of TCGA datasets

Cbioportal for cancer genomics was accessed (https://www.cbioportal.org/) to download RNA sequencing data (RSEM - Batch normalized from Illumina HiSeq_RNASeqV2) for the 8 signature genes for the following datasets: Breast Invasive Carcinoma (TCGA, PanCancer Atlas); Colorectal Adenocarcinoma (TCGA, PanCancer Atlas); Ovarian Serous Cystadenocarcinoma (TCGA, PanCancer Atlas) and Pancreatic Adenocarcinoma (TCGA, PanCancer Atlas). Subsequently the patients in each dataset were given a hypoxia score as previously described (Abou Khouzam et al., 2021). In brief, for every independent dataset the median expression of each gene was first calculated and then each patient was given a gene score, that was of a value of 1 or -1, depending on whether the particular gene was expressed more or less than the median respectively. The hypoxia score was then calculated as the sum of the gene scores. The dataset was then stratified into hypoxia high (HH) or hypoxia low (HL) based on whether the hypoxia score was greater than zero, or less than or equal to zero. The patient IDs were then used to create the HH and HL groups on cbioportal and genomic alteration events were compared.

### 2.3 Culture conditions

The three highest scoring cell lines, H226, MCF-7 and HT-29, as well as the three lowest scoring cell lines, Capan-1, SKOV-3, MIA PaCa-2 were maintained in a 5% CO_2_, 21% O_2_ humidified incubator (ESCO Cell Culture incubator, USA). They were cultured in appropriate Dulbecco’s Modified Eagle Medium/F-12 (DMEM/F-12), GlutaMAX Supplement (Cat. No 31331028; Thermo Fisher Scientific, Inc. United States) or in Roswell Park Memorial Institute Medium 1640 (RPMI 1640) Medium, GlutaMax supplement (Cat. No. 61870010; Gibco, ThermoFisher Scientific, United States), supplemented with 10% Fetal Bovine Serum (FBS) (Cat. No. 10270106; Gibco, ThermoFisher Scientific, United States), 1% Sodium pyruvate (Cat. No. 11360039; Gibco, ThermoFisher Scientific, United States) and 1% Penicillin - Streptomycin (Cat. No. 15140122; Gibco, ThermoFisher Scientific, United States), as previously described (Abou Khouzam et al., 2020a). Two independent experiments were conducted using early passages from *mycoplasma* free cells. At each seeding time, cells were allowed to adhere for 24 h before incubating in hypoxia or keeping back in normoxia. Hypoxic conditions were set at 1% O_2_, 5% CO_2_ and maintained using the humidified Whitley H35 HypoxyStation (Don Whitley123 Scientific Limited, UK). Cells were passaged twenty times in the two conditions and DNA and RNA was isolated and processed as described below.

### 2.4 Whole transcriptome analysis by microarrays

#### 2.4.1 RNA isolation and arrays preparation

Total RNA was first isolated using EasyBlue (iNtRON Biotechnology, Korea) following manufacturer’s instructions, and subsequently purified with repeated washes and in 75% ethanol. After drying, isolated pellets were resuspended in nuclease free DEPC treated water and concentration and purity were evaluated using the Nanodrop. The integrity of RNA was determined by running 300 ng on 1% agarose gel and the 28S/18S bands were observed. Only RNA with acceptable integrity (28S twice the intensity of 18S and without smearing), as well as purity ratios (260/280 between 1.8–2.0, and 230/230 ratio of greater than 2.0) were processed for transcriptome analysis with the Human Clariom™ D Assay (Affymetrix, Applied Biosystems™). Aliquots of 50 ng/μL were prepared and 100 ng of total RNA was used to prepare fragmented and labeled single stranded cDNA. The steps were conducted with the GeneChip™ WT PLUS Reagent Kit (Applied Biosystems™) following manufacturer’s instructions. Each sample was hybridized to one array respectively by placing the arrays in specialized trays and incubating them for 17 h at 45°C and 60 rotations per minute using the Genechip^®^ Hybridization oven 640 (Affymetrix). Arrays were subsequently washed and stained using appropriate reagents from the GeneChip™ WT PLUS Reagent Kit (Applied Biosystems™) as well as the GeneChip Fluidics Station 450 (Affymetrix/Thermo Scientific inc) following manufacturer’s recommendations. The arrays were immediately scanned with the GeneChip™ Scanner 3000 7G (Affymetrix/Thermo Scientific inc).

#### 2.4.2 Gene expression analysis

The images and raw data files from scanned arrays were first checked to ensure correct alignment and that there were no bubbles or debris affecting the image’s clarity. Subsequently, cell probe defined files (.CEL) files were processed using the Transcriptome Analysis Console (TAC) 4.2 software (Applied Biosystem). Gene + Exon summarization using the Signal Space Transformation-Robust Multi-Array Average (SST-RMA) algorithm was first applied to summarize, background-subtract, and normalize the CEL files. Further QC checks were applied to ensure that all samples were properly labeled and hybridized and had acceptable positive signal intensity. The resulting CHP files were imported back to TAC to carry out differential gene expression analysis using ANOVA method for statistical testing with ebayes (Empirical Bayes Statistics for Differential Expression) correction for small sample size. The hypoxia samples in each cell line were compared to their respective normoxia samples from passage zero and passage twenty. Only transcript clusters (TCs) that showed a fold change (FC) of ≤ −2 or ≥2 with a false discovery rate (FDR) *p*-value ≤0.05 were considered significant. The lists of significant differentially expressed TCs per comparison per cell line can be found in Supplementary File S1.

#### 2.4.3 Pathway enrichment analysis

Pathway analysis on the differentially expressed genes per cell line was conducted using GSEA v4.2.2. Default settings were used (1000 permutations, with weighted enrichment statistic, real gene list sorting mode and descending gene list ordering mode), except that the permutation type was gene_set and not phenotype, given that the sample numbers per group were less than 7. The metric for ranking genes was the ratio of classes, which is equivalent to the fold change. Curated gene sets from HALLMARK (//pub/gsea/gene_sets/h.all.v7.5.1. symbols.gmt,ftp.broadinstitute.org), KEGG (//pub/gsea/gene_sets/c2. cp.kegg.v7.5.1. symbols.gmt,ftp.broadinstitute.org), REACTOME (//pub/gsea/gene_sets/c2. cp.reactome.v7.5.1. symbols.gmt,ftp.broadinstitute.org), BIOCARTA (//pub/gsea/gene_sets/c2. cp.biocarta.v7.5.1. symbols.gmt,ftp.broadinstitute.org) and WIKIPATHWAYS (//pub/gsea/gene_sets/c2. cp.wikipathways.v7.5.1. symbols.gmt) were used for enrichment analysis. Enriched pathways are given with a normalized enrichment score (NES) that is an enrichment score which has been normalized across the analyzed gene-sets. The enrichment score itself, represents the degree to which a particular gene-set is over-represented at the top or bottom ranked list of genes present in the expression data. Significantly enriched pathways were considered as those having a NES with an FDR q-value of at least 0.05.

#### 2.4.4 Alternative splicing analysis

Potential alternative splicing was assessed due to the presence of probe selection regions (PSRs) that represent gene exons and junctions (JUCs), which are used to probe the inter-exon boundary. Eventpointer algorithm is applied to predict the presence of alternative splicing events considering the signals of the JUCs and PSRs upon their normalization to the expression levels of the gene itself. The summary expression signals of JUCs and PSRs are then used to determine the splicing index in condition 2 (hypoxia) compared to condition 1 (normoxia). TC with exon splicing index of greater than 2 or less than −2 and an exon FDR *p*-value of less than 0.05 are considered differentially expressed. In addition, based on the signals of the PSRs and JUCs of a gene, the event of alternative splicing is determined as being an exon cassette (splicing out of exon from primary transcript), intron retention (either an intron sequence is retained, or a sequence is spliced out as an intron, in mature mRNA transcript), alternative 5′ donor site (presence of 2 or more splices at the 3′ end of the exon indicating the presence of alternative 5′ splice junction, changing the 3′ boundary of the upstream exon), alternative 3′ acceptor site (presence of 2 or more splices at the 5′ end of the exon indicating presence of alternative 3’splice junction, changing the 5′ boundary of the downstream exon), mutually exclusive exons (2 back to back exons excluded from the mature mRNA transcript), alternative last exon and alternative first exon, as well as complex events that do not fit in these categories. How well the event explains the splicing pattern visualized is given by an event score with values from 0 – 1. The event with the highest value is considered as the most possible mode of alternative splicing. Alternatively spliced TC without any reported event score were excluded from the analysis.

### 2.5 Whole exome sequencing and data analysis

#### 2.5.1 DNA isolation, library preparation and sequencing

Genomic DNA was isolated using the PureLink^®^ Genomic DNA Kit (Invitrogen, Life Technologies) following manufacturer’s recommendation. In brief, DNA pellets were lysed by vortexing in 200 μL PureLink^®^ Genomic Lysis/Binding Buffer following treatment with Proteinase K and RNase A. DNA was subsequently bound on the PureLink^®^ Spin Columns upon addition of 200 μL absolute ethanol. The DNA was washed with appropriate buffers, which were removed by centrifugation and finally eluted using 40–60 μL low binding TE buffer (Ion Torrent™, ThermoFisher Scientific). The purity of gDNA was confirmed with the Nanodrop and the integrity was checked by running 500 ng on agarose gel electrophoresis. The Ion S5 System (Ion Torrent™, ThermoFisher Scientific) was used for whole exome sequencing. Before starting, gDNA was accurately quantified by fluorometry using the Qubit dsDNA HS Assay Kit (Q32851, Invitrogen) and the Qubit 4 Fluorometer (Invitrogen). Subsequently 100 ng of DNA was used for exome library preparation with Ion AmpliSeq™ Exome RDY Kit and Ion Xpress™ Barcode Adapters 17–32 Kit (4474009, Ion Torrent™) or Ion Xpress™ Barcode Adapters 33–48 Kit (4474518, Ion Torrent™) following manufacturer’s protocols. For every sample, target regions were amplified by PCR and the resulting amplicons were pooled, partially digested then ligated with suitable adaptors to achieve barcoded libraries. Libraries were purified using the CleanNGS system (Cleanna, CNGS-0500) then finally quantified by qPCR using the Ion Library TaqMan Quantification Kit (4468802, Ion Torrent™) and diluted to 100pM. The Ion 540™ Kit–Chef (A30011, Ion Torrent™) was used to implement the sequencing reactions following recommended protocols. The Ion template preparation on 540 Ion Chip™ was done using the automated Ion Chef™ System (Ion Torrent™) and the sequencing reaction was carried out in the Ion S5™ sequencer (Ion Torrent™). Two differentially barcoded libraries could be sequenced simultaneously at 25 pM per library.

#### 2.5.2 Data analysis

Raw sequencing data was first aligned against hg19 with Ion Torrent Suite software to generate Binary Alignment Map (BAM) files. Low stringency somatic variant and indel calling was then conducted with the Torrent Variant Caller by applying default settings to obtain Variant Call Format (VCF) files. VCF files were converted to MAF files using *vcf2maf*, as described in (Zaarour et al., 2022). Two Capan-1 files were manually checked during the vcf to maf conversion for rows that generated an error. Those rows (*n* = 2 across both the files) were removed as a part of the data cleaning process from further analysis. Furthermore, two MIA PaCa-2 files were removed from the downstream analyses as they generated TMB levels that were 300 orders of magnitude higher than all values achieved for this cell line, as well as the other cell lines. The TMB values were also higher than those previously reported for the cell line as per the cancer cell line encyclopedia on cbioportal (https://www.cbioportal.org/). Therefore, there were 44 files that were analyzed further. The full information of these samples can be found in the Supplementary Table S1. There were two technical replicates per experiment and there were two experiments or biological replicates per cell line, i.e., a total of four samples (two biological replicates with two technical replicates per biological replicate). For the analysis, there were four pairs of matched control (normoxia) and treated (hypoxia) samples. For example, a technical replicate 1 for a biological replicate 1 of normoxia was used for the corresponding technical replicate 1 for a biological replicate 1 of hypoxia sample for a given cell line. The mutations reported are the ones that are only present in hypoxia after 20 passages and not present in normoxia sample. All the analysis was run on individual cell lines and after grouping them into two groups: Hypoxia High or HH (MCF-7, H226, HT29) and Hypoxia Low or HL (Capan-1, SKOV-3, MIA PaCa-2).

R package *maftoolsv2.6.05* was used to analyze MAF files. Mutational landscape across the samples HL was analyzed after normalizing the variants called in the hypoxia-treated cancer cells against the normoxia cancer cells used as the control. In this way only variants that emerged in the cancer cells after exposure to long term hypoxic conditions could be identified. The mutational landscape was then reported as follows: 1. Tumor Mutational Burden, 2. Gene mutation summary and 3. Sample mutational summaries. Next, Copy Number Variations (CNVs) were calculated using the command line version of Control-FREEC. The analysis was run on. bam files. Control-FREEC requires an additional custom config file with all the parameter values to be run according to the data input and the desired output. CNVs were computed, normalized, and segmented for each hypoxia tumor sample against a corresponding normoxia sample as a control dataset using a window size of 50kb. breakPointThreshold was set to 0.8, contamination parameter was set to 0, minMappabilityPerWindow was set to 0.85 and ploidy was set to multiple values of 2, 3, 4 and the ploidy was allowed to be chosen according to the value that explains most observed CNVs across all samples. All the supporting files including input, output, config and codes written to carry out the analysis can be found at the following github link: https://github.com/Mohak91/hypoxia_pan_cancer_types_genomics_study.

### 2.6 Reverse transcription quantitative PCR (RT-qPCR) for validation of gene expression

Reverse transcription reactions were conducted on 1 μg RNA using the High-Capacity cDNA Reverse Transcription Kit (4368814, Applied Biosystems™) following manufacturer’s instructions. Twenty nanograms of cDNA were subjected to 10 μL qPCR reactions run in triplicate with the Maxima SYBR Green/ROX qPCR Master Mix (2X) (K0223, Thermo Scientific™). Select genes were quantified by RT-qPCR using specifically designed primers (Supplementary Table S2). Quantitative PCR reactions were run on the CFX384 Touch Real-Time PCR Detection System (Bio-Rad) and fold change was calculated by applying the 2^−ΔΔCq^ method (Livak and Schmittgen, 2001). Beta actin was included as endogenous control.

### 2.7 Statistical analysis

Statistical analysis was conducted using GraphPad Prism 9.3.1. Unpaired two-sided t-test was applied for comparisons between only two groups, otherwise 2-way ANOVA with the recommended correction for multiple comparisons was used. Statistical significance was considered as *p*-value ≤0.05. Heatmaps of expression were generated using Rstudio version 1.3.1073 (RStudio, PBC, United States) by applying the gplots package. Hierarchical clustering was performed using Euclidean distance and ward. D2 method.

## 3 Results

### 3.1 A hypoxia metric distinguishes the hypoxic response of cancer cells

**FIGURE 1:**
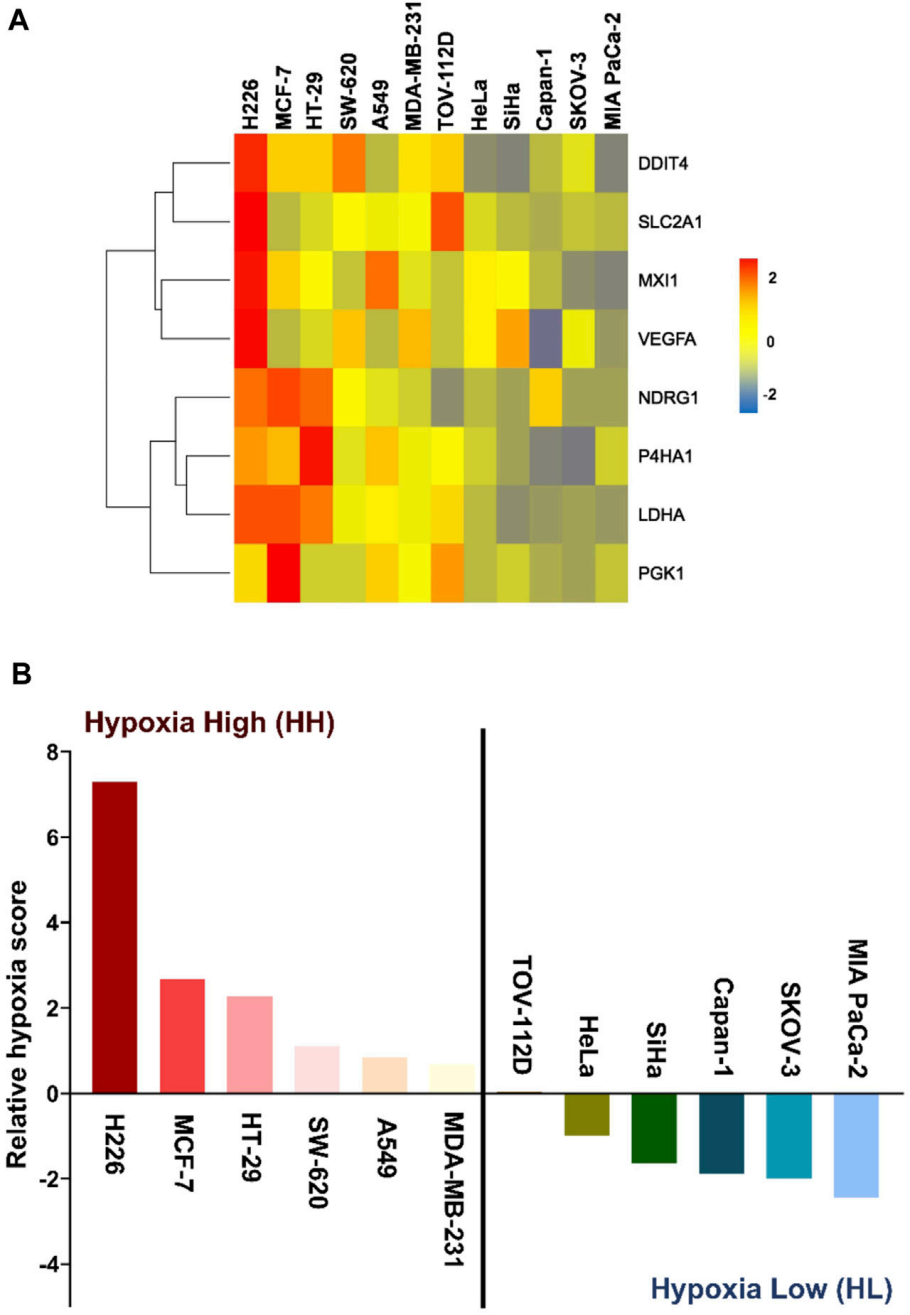
Hypoxia-scored cancer cell lines. **(A)** Heatmap of the fold change of the eight genes across the twelve cancer cell lines. **(B)** Relative hypoxia score of each cell line ordered from highest to lowest. Cell lines with relative hypoxia score greater than zero are considered as hypoxia high (HH) and those less than zero are considered as hypoxia low (HL).

We have recently derived an *in vitro* 8-gene hypoxia signature (Abou Khouzam et al., 2020a). The characteristic response of twelve cancer lines to hypoxia was evaluated by first calculating the gene score of each of the 8 genes in the signature as the mean fold change of three independent experiments. As shown in the heatmap (Figure 1A), the degree of response to hypoxia varied from extremely low in the pancreatic ductal carcinoma cell line (MIA PaCa-2), to remarkably high in the squamous cell carcinoma cell line (H226). This gradient of response was then measured by giving each cell line a hypoxia score calculated as the geometric mean of all 8 gene scores. The hypoxic response of the different cell lines was then compared by plotting their relative hypoxia scores (Figure 1B). Two groups could be distinguished, the hypoxia high (HH) group, which displayed higher sensitivity to hypoxia, and the hypoxia low (HL) group, having much lower sensitivity to this condition. To further dissect the differential impact of hypoxia on the two groups, the three highest and three lowest cancer cell lines were subjected to twenty passages in hypoxia and normoxia and subsequently interrogated at the transcriptomic and genomic levels.

### 3.2 Cancer cells present both a common and cell-line specific response to hypoxia

**FIGURE 2:**
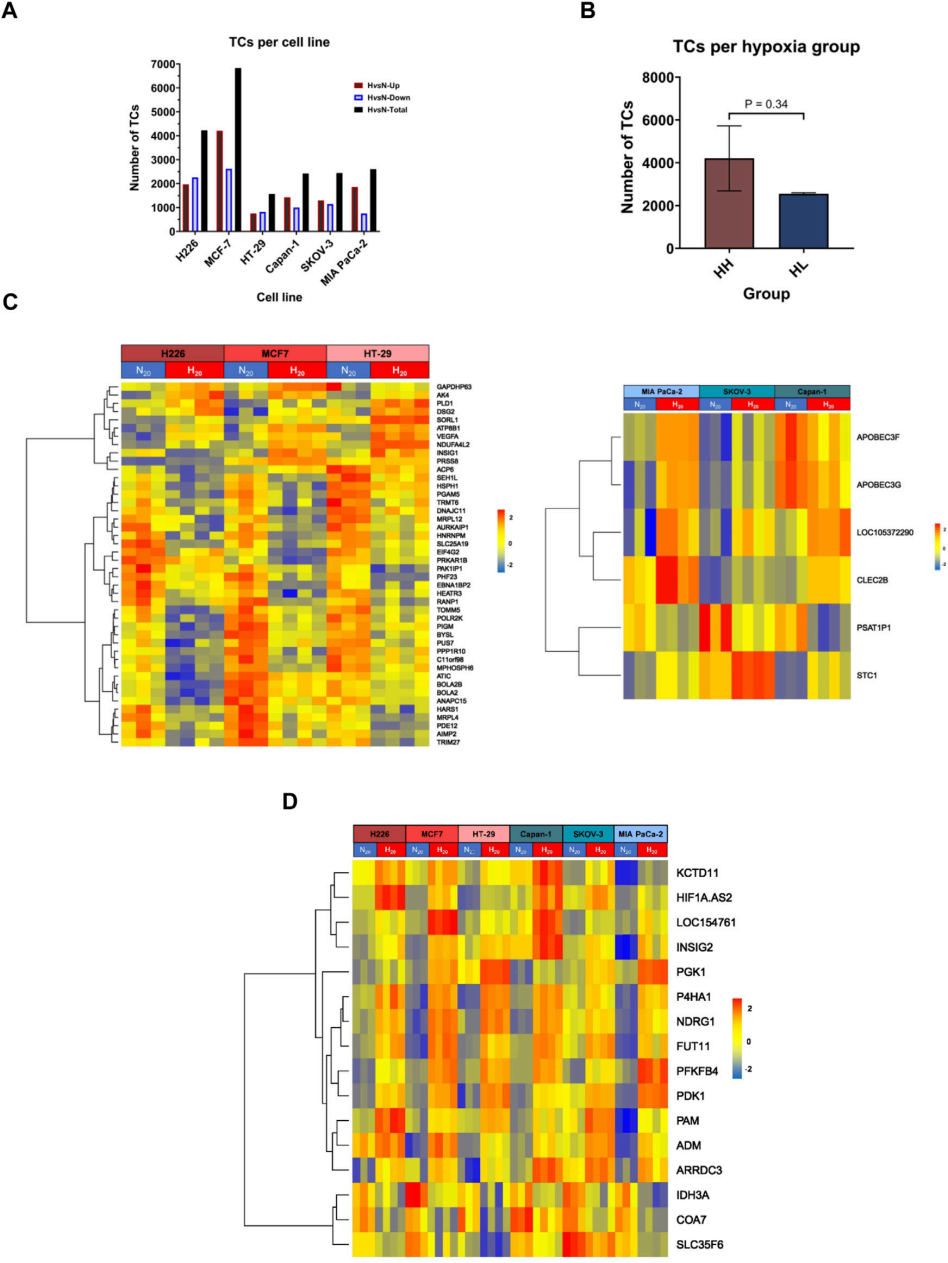
Differentially expressed transcripts in hypoxia. **(A)** The total number of transcript clusters (TCs) and the downregulated and upregulated number of TCs in hypoxia (H) *versus* normoxia (N) in each cell line. **(B)** The mean number of differentially expressed TCs in hypoxia high (HH) *versus* hypoxia low (HL) groups. **(C)** Heatmaps of the HH specific differentially expressed genes (left panel) and HL specific differentially expressed genes (right panel). **(D)** Heatmap of the common differentially expressed genes in both HH and HL groups. Statistical analysis based on unpaired two-tailed t-test with *p* ≤ 0.05 considered statistically significant.

To evaluate the impact of chronic hypoxic conditions on transcriptional remodeling within the two hypoxia groups, RNA obtained following twenty passages in normoxia or in hypoxia was subjected to microarray analysis. Among the 135′750 transcript clusters (TCs) (also known as probe-sets) identified on the chip, 14′799 unique transcript clusters were differentially expressed in at least 1 cell line. The bulk of response in all cell lines, except for HT-29, was cell-line specific. Indeed, the biggest contributing factor to the variation seen among samples was their cell line-dependent characteristics (Supplementary Figure S1). The cell lines clearly exhibited variable severity of response to hypoxia. Both the lowest and the highest number of differentially expressed TCs were observed in the HH cell lines, HT-29, and MCF-7 respectively. The HL group showed a more consistent number of differentially expressed TCs, varying between 2′400 and 2′500 transcripts (Figure 2A). On average, the HH group demonstrated a stronger response to hypoxia, as determined by a higher number of TCs; however, the difference between the two groups did not reach statistical significance (Figure 2B). In addition, 45 differentially expressed genes (DEGs) were common to the HH group, while only 6 could be specifically assigned to the HL group (Figure 2C; Supplementary Table S3) and 16 were common among both groups (Figure 2D; Supplementary Table S3).

Regarding the HH specific genes, two genes showed variable directionalities of expression, nine were upregulated, and the majority (33 genes), were downregulated in hypoxia. The expression of five of these genes was validated by RT-qPCR (Supplementary Figure S2A). In particular, the stress response gene *AK4*, the pro-angiogenic gene *VEGFA* and the cell adhesion gene *DSG2* were upregulated in all 3 cell lines, while the translation regulation gene, *EIF4G2* and the mitochondrial gene *PGAM5* were downregulated. Among the six HL specific genes, two showed variable expression (*APOBEC3F*, *APOBEC3G*), while one was consistently downregulated (*PSAT1P1*) and three upregulated (*LINC01764*, *STC1* and *CLEC2B*) in all 3 cell lines. The upregulation of the cell adhesion gene *CEC2B* and the glycoprotein hormone *STC1* were validated by RT-qPCR (Supplementary Figure S2B).

In terms of the common response to hypoxia that was achieved in all cell lines, three genes were downregulated, two of which are involved in metabolism, while the remaining thirteen genes were consistently upregulated across all 6 cell lines. Those included long non-coding RNAs, genes involved in glycolysis, apoptosis, extracellular matrix (ECM) remodeling, proliferation, and angiogenesis (Supplementary Table S3; Supplementary Figure S2C). Of interest, among the 8 signature genes, only three genes, namely *PGK1*, *NDRG1* and *P4HA1*, maintained significant differential expression in all cell lines. Significant *VEGFA* increased expression was also maintained in the HH group.

In addition to its impact on gene transcription, long incubation under hypoxic conditions affected the nature of RNA being expressed. There was strong differential splicing in hypoxia compared to normoxia across all cell lines, ranging from 774 transcripts in HT-29 to 3′304 in MCF-7 (Supplementary Figure S3A, Supplementary File S2). The number of alternatively spliced transcripts was clearly cell-line dependent and did not vary between the two hypoxia groups (Supplementary Figure S3B). The main predicted event behind the alternatively spliced transcripts across the cell lines was described as cassette exon, in which an exon is spliced out from the primary transcript (Supplementary Figure S3C). Four transcripts, namely *PDK1*, *NDRG1*, *LDHA*, and *SLC3A2* were alternatively spliced in all the cell lines. In addition, *HIF-1α* was significantly spliced in all cell lines (Supplementary File S2). However, no splicing event could be attributed in HT-29 and so it was filtered out from the analysis. Clearly, even after long incubation in hypoxia, hypoxia-modulated genes are prone to alternative splicing events that could impact their functional state.

### 3.3 Transcriptional remodeling of ranked tumor cells under hypoxic stress

Pathway analysis was conducted using GSEA and by surveilling the curated Hallmark, KEGG, REACTOME, biocarta, and wiki gene-sets. Only pathways with an FDR q-value ≤0.05 were considered significantly enriched (Supplementary File 3). There were 175 pathways in H226 (51 specific), 183 pathways in MCF-7 (63 specific), 26 pathways HT-29 (2 specific). With respect to the HL group, there were 58 pathways in Capan-1 (6 specific), 34 pathways in SKOV-3 (16 specific) and 47 pathways in MIA PaCa-2 (3 specific) (Supplementary File 3). Taken together, the HH group observed a higher number of enriched pathways compared to the HL group.

**FIGURE 3:**
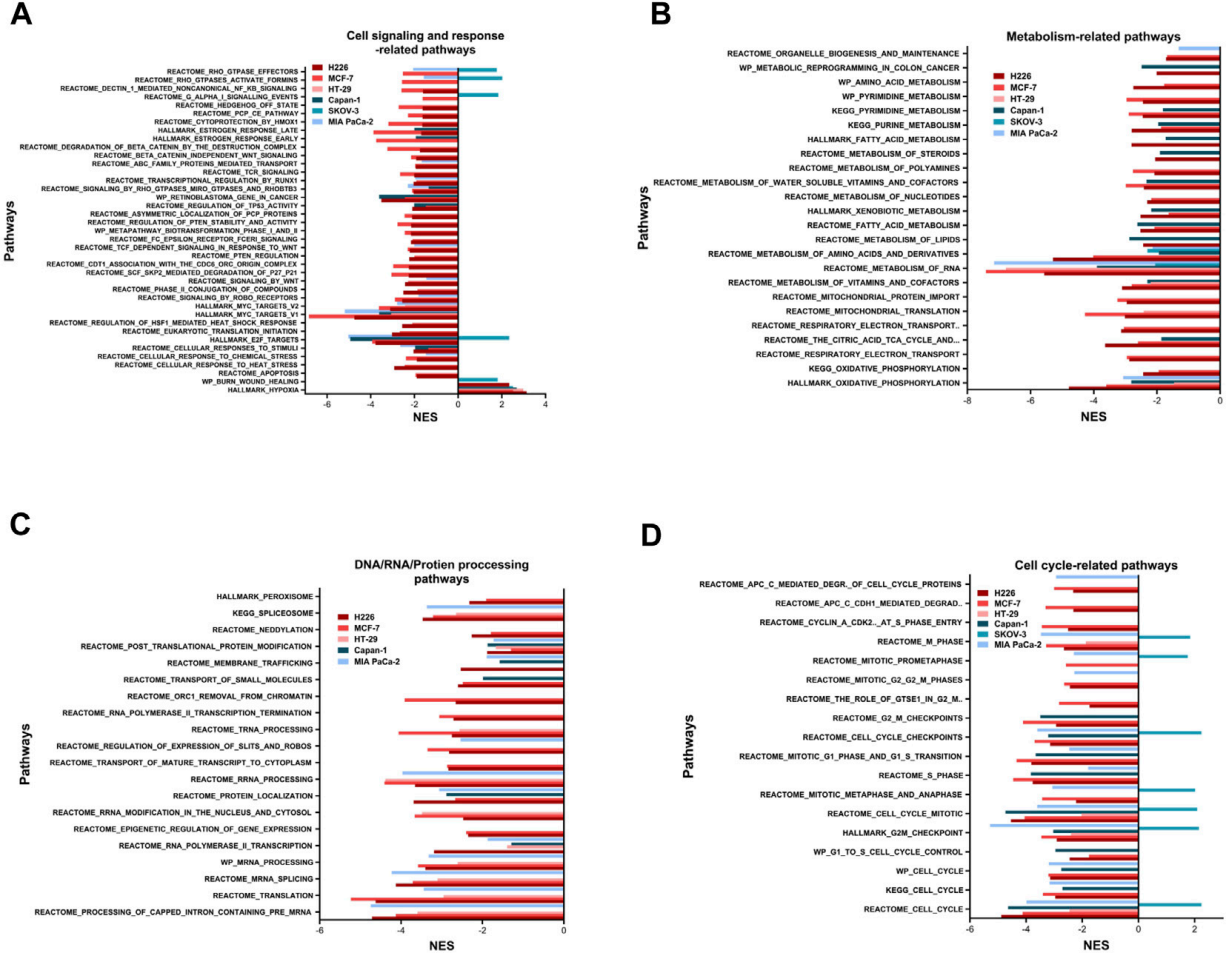
Pathways enriched following hypoxic stress. Cell signaling and response-related pathways **(A)**, metabolism-related pathways **(B)**, DNA, RNA, or protein processing-related pathways **(C)** and cell cycle-related pathways **(D)** significantly enriched in the hypoxic (NES>0) or the normoxic (NES<0) condition based on gene set enrichment analysis. Only pathways with FDR q-value ≤0.05 are included. NES: normalized enrichment score.

The enriched pathways could be grouped into six major subsets: cell signaling and response; metabolism; DNA/RNA/protein processing; cell cycle; DNA replication and repair; and immune response (Figures 3, 8, 9). The only pathway that was positively enriched in hypoxia across all 6 cell lines was hypoxia, and the highest normalized enrichment score (NES) for that was demonstrated in H226, followed by HT-29, Capan-1, MCF-7, SKOV-3 and finally MIA PaCa-2 (Figure 3A). In addition, EMT-related gene-set in the Burn-Wound healing wiki pathway was upregulated in H226 and SKOV-3 (Figure 3A). All cell lines showed significant hypoxia-dependent decrease in pathways of metabolism. This was especially evident in H226, MCF-7, and Capan-1. HT-29 and MIA PaCa-2 showed the highest negative enrichment for RNA metabolism, and all cell lines except for SKOV-3 exhibited a decrease in oxidative metabolism (Figure 3B). With respect to pathways involved in processing of DNA, RNA, and proteins, none were enriched in SKOV-3. The bulk of the effect was observed in H226, MCF-7, HT-29, and MIA PaCa-2 for RNA processing pathways. Capan-1 appeared to display negative enrichment mainly in pathways involved in protein processing (Figure 3C). In terms of cell cycle-related pathways, apart from SKOV-3, all cell lines demonstrated a decreased enrichment of the cell cycle, suggesting reduced division capacity in hypoxia (Figure 3D).

Other pathways positively enriched in hypoxia were seen in a cell line dependent manner (Supplementary Figure S4). H226 depicted a unique enrichment of inflammatory response and interferon-related response signaling pathways. In addition, this cell line was positively enriched for TNFα signaling *via* NFκB, among others. On the other hand, MCF-7 demonstrated hypoxia-specific enrichment of the P53 transcriptional network and pathway, while HT-29 was enriched for glycolysis. MIA PaCa-2 and Capan-1 did not show any specific increased enrichment in hypoxia, while SKOV-3 showed the highest number of increased pathways in hypoxia, including pathways of EMT, Extracellular matrix degradation and organization, apical junction, and focal adhesion among others. Indeed, this cell line had quite a unique response to hypoxia, while most of the cell-cycle, and cell-signaling pathways were downregulated in hypoxia for the cell lines, in SKOV-3, eukaryotic translation initiation, Dectin1-mediated non-canonical NFκB signaling, and RHO GTPase related signaling were upregulated; so were pathways of the cell-cycle and cell-cycle checkpoints and control.

Based on these results, it can be concluded that long passaging in hypoxia leads to cell cycle inhibition, decreased metabolic activity as well as nucleic acid and protein processes. Our data also indicate that cells seem to switch to an energy conserving and indolent state.

### 3.4 Higher sensitivity to hypoxia could be associated with increased mutability

To determine the relationship between the sensitivity of cancer cell lines to hypoxia with their ability to accumulate mutations, the three highest scoring cell lines, H226, MCF-7 and HT-29, and three lowest scoring cell lines, Capan-1, SKOV-3, and MIA PaCa-2 were cultured in chronic hypoxic conditions, indicated as twenty passages at 1% O_2_. Mutational landscape across the samples was analyzed after normalizing the variants called in the hypoxia-treated cancer cells against the normoxia cancer cells used as the control.

**FIGURE 4:**
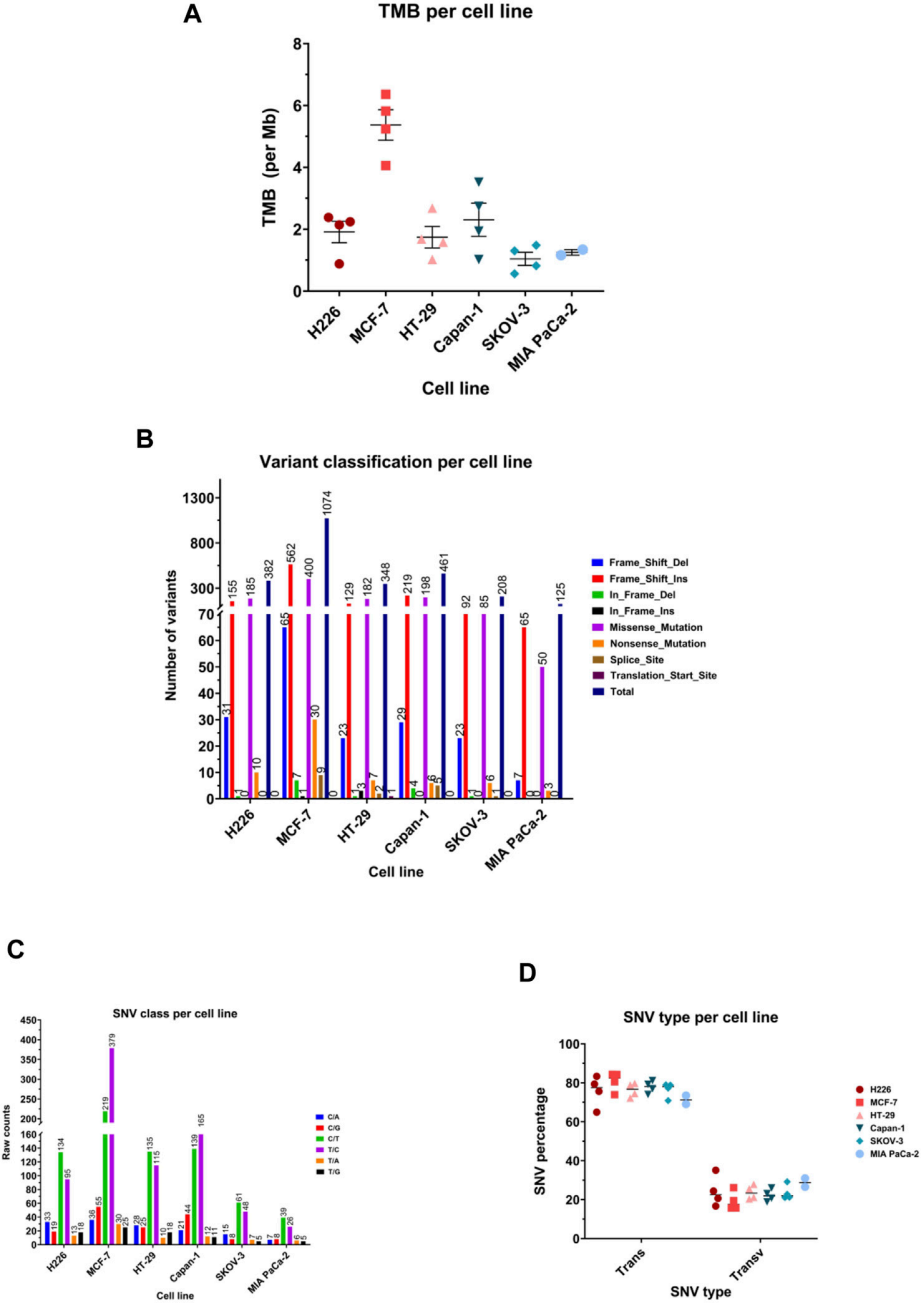
Mutational landscape of the highest and lowest scoring cancer cell lines. **(A)** Median tumor mutational burden (TMB) per megabase (Mb) of genome. Each symbol represents one sample. **(B)** Number of variants in the different variant classifications. **(C)** Number of single nucleotide variant (SNV) in each class. **(D)** Percentage of SNVs that are transitions (Trans) and Transversions (Transv) per cell line. Each symbol represents one sample.

TMB achieved across cell lines was variable, ranging from a mean of 1.040 mutations/Mb in SKOV-3 (95%CI: 0.3649–2.394) to 5.37 mutation/Mb (95%CI: 3.801–6.939) in MCF-7 (Figure 4A). All cell lines showed frame shift insertions as the most common variant type, followed by missense mutations, except for HT-29 and H226, which suffered more missense mutations than frameshift insertions (Figure 4B). Regarding the class of SNV, C>T transitions were the most occurring type in all except MCF-7 and Capan-1, in which T>C substitutions topped the list (Figure 4C). Indeed, the majority of the SNVs were transitions consistently in all cell lines (Figure 4D).

**FIGURE 5:**
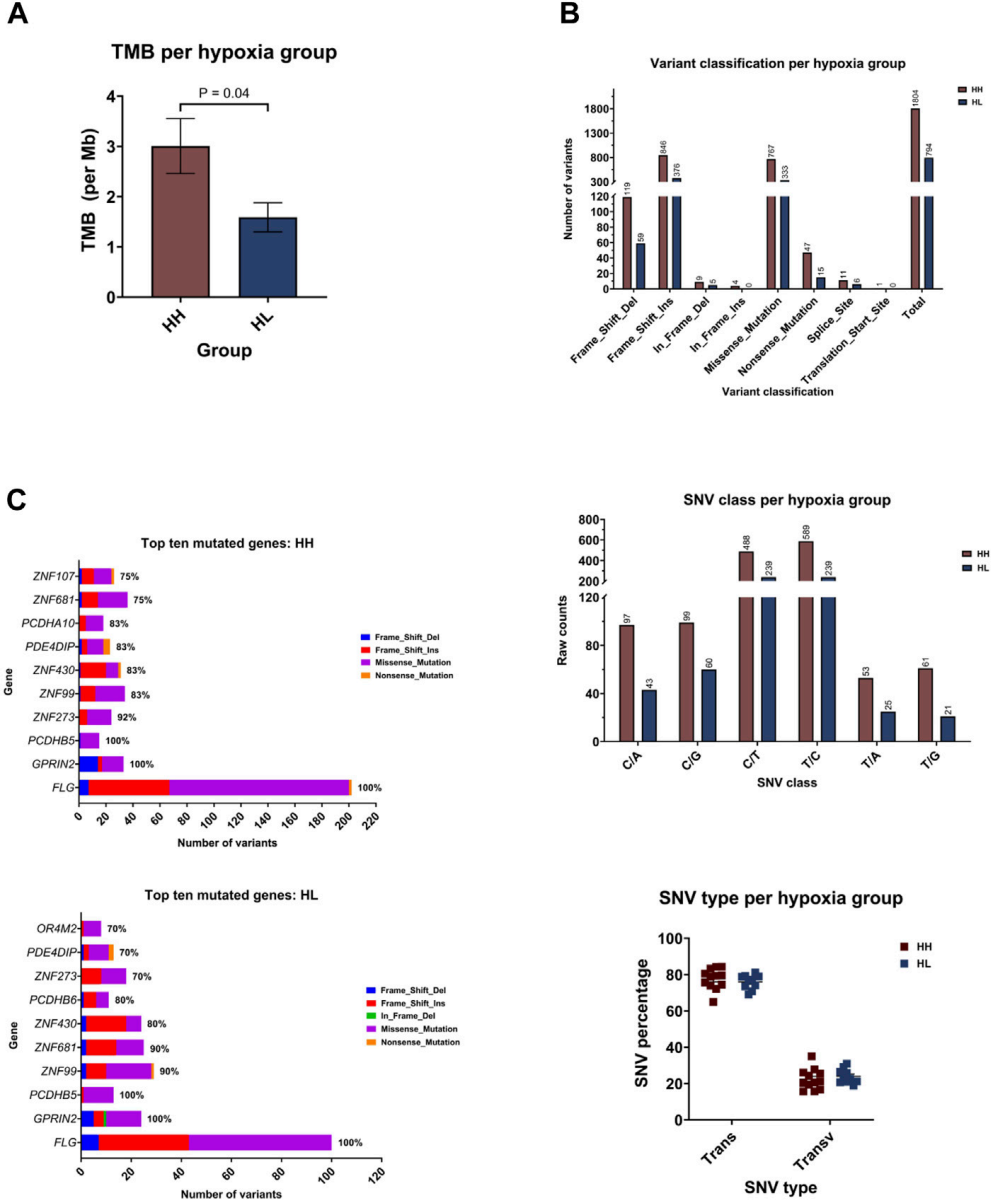
Mutational landscape of the hypoxia high (HH) versus hypoxia low (HL) groups. **(A)** Mean tumor mutational burden (TMB) per megabase (Mb) of genome for each group. **(B)** Number of variants in the different variant classifications; number of single nucleotide variant (SNV) in the different classes and the percentage of SNVs that are transitions (Trans) and Transversions (Transv) **(C)** The number and type of variants in the top ten mutated genes with the percentage of samples harboring mutations in each gene.

Comparing the hypoxia high (HH) to the hypoxia low (HL) groups, HH displayed a mean of 3.007 mutations per megabase of genome (95%CI: 1.805–4.209), while the HL group accumulated 1.588 mutations (95%CI: 0.9353–2.241) (Figure 5A). Indeed, there was a significantly higher TMB in the HH group (*p* = 0.03), suggesting that cells that are more sensitive to hypoxia are also more mutable. Looking into the classification of identified variants, the majority were frame-shift insertions, followed closely by missense mutations in both hypoxia groups with the most common form being C>T and T>C transitions (Figure 5B). Among the top ten mutated genes, *FLG*, *GPRIN2* and *PCDHB5* were mutated in all samples (Figure 5C).

**FIGURE 6:**
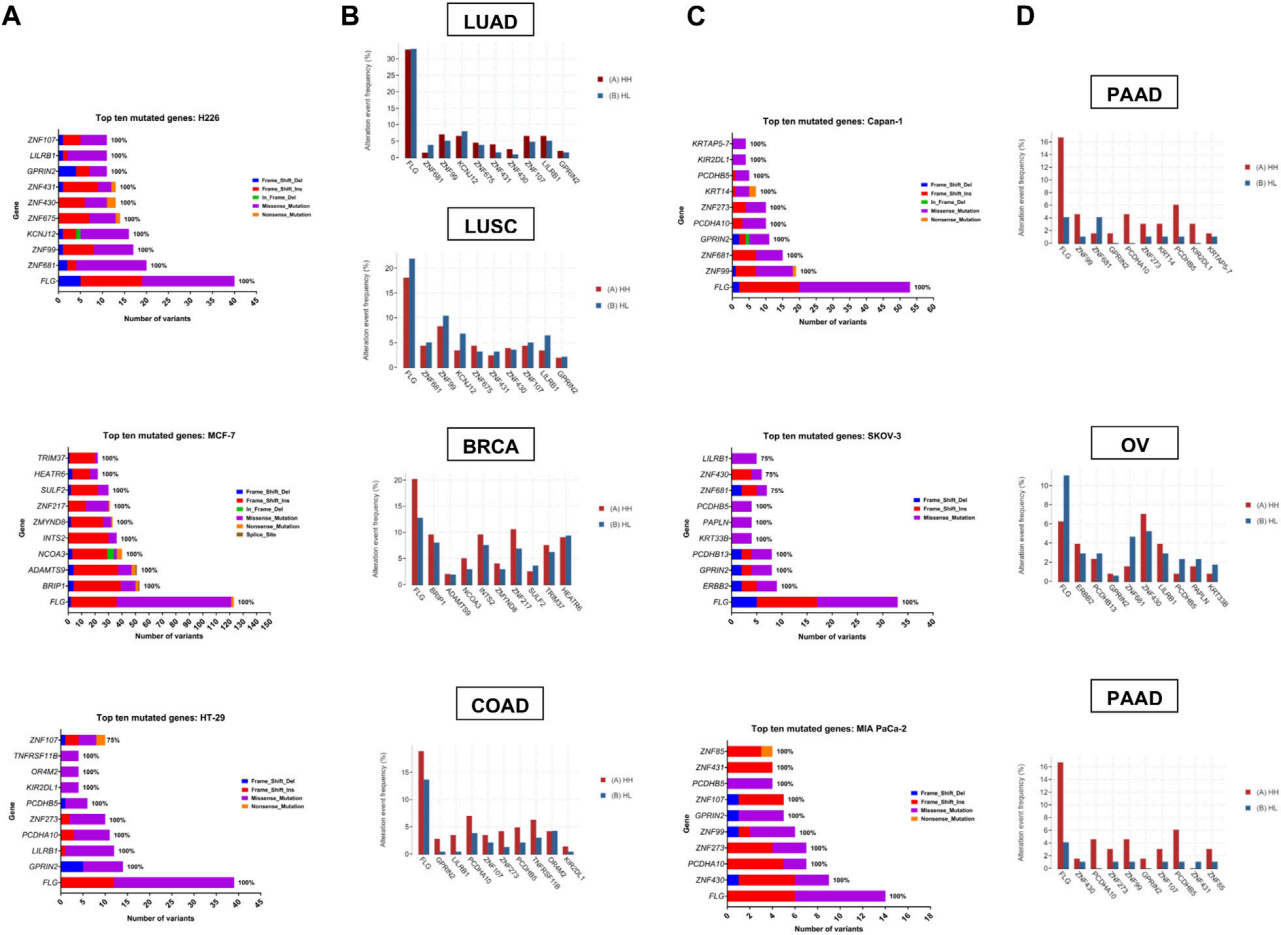
Top ten mutated genes in each cell line and corresponding tumor TCGA dataset. **(A, C)** Number and type of variants in the top ten mutated genes with the percentage of samples harboring mutations in each gene. Color code reflects the variant class. **(B, D)** The frequency (in percent) of alteration events of the top ten mutated genes in hypoxia high (HH) *versus* hypoxia low (HL) patient groups with lung adenocarcinoma (LUAD), lung squamous cell carcinoma (LUSC), breast invasive carcinoma (BRCA), colorectal adenocarcinoma (COAD), pancreatic ductal adenocarcinoma (PDAC) and ovarian serous cystadenocarcinoma (OV).

Zooming in on the expression patterns of the top ten mutated genes per cell line (Figure 6A, C), as well as the three genes mutated in all samples, *FLG*, *GPRIN2* and *PCDHB5*, no effects could be reported for most of the genes, however, in the case of MCF-7, seven genes were affected. One was differentially spliced (*BRIP1*), two were only differentially expressed (*HEATR6*, *ZNF217*) and four were both differentially expressed and spliced (*ADAMTS9*, *INTS2*, *SULF2*, *ZMYND8*) (Supplementary Tables S4, S5). With respect to the remaining cell lines, one gene (*ZNF431*) was differentially expressed in H226, and one (*ZNF273*) differentially spliced in MIA PaCa-2 (Supplementary Tables S4, S5). These results, however, do not negate the potential impact of the observed gene mutations on protein expression and function.

### 3.5 Validation of hypoxia-mutated genes in cancer patients

To determine whether the affected genes *in vitro* were also altered in patient samples, we applied our signature to stratify tumors of TCGA datasets based on their hypoxia score into high hypoxia and low hypoxia. For each cancer type, the percent alteration of the top ten *in vitro* mutated genes were compared within the two groups. Results showed a good concordance level between the genes that were reported as affected *in vitro* and their mutation in more hypoxic patient tumors (Figure 6). This was especially evident in pancreatic and colorectal cancer, where 9/10 of the top mutated genes *in vitro* showed higher alteration frequency in hypoxia. In addition, 8/10 genes and 7/10 genes were altered at a higher frequency in hypoxia, in the breast cancer and the lung adenocarcinoma datasets, respectively. On the other hand, OV and LUSC showed very little concordance with the *in vitro* results, with only 3/10 and 1/10 genes showing increased alteration events in hypoxia. These results underscore the relevance of sequencing results from *in vitro* investigation of tumor cells, given that data from patient tumors is usually not limited to tumor cells and is contaminated with sequencing results from other cells residing in the microenvironment, including immune and stromal cells.

### 3.6 Hypoxia is associated with increased chromosomal instability

**FIGURE 7:**
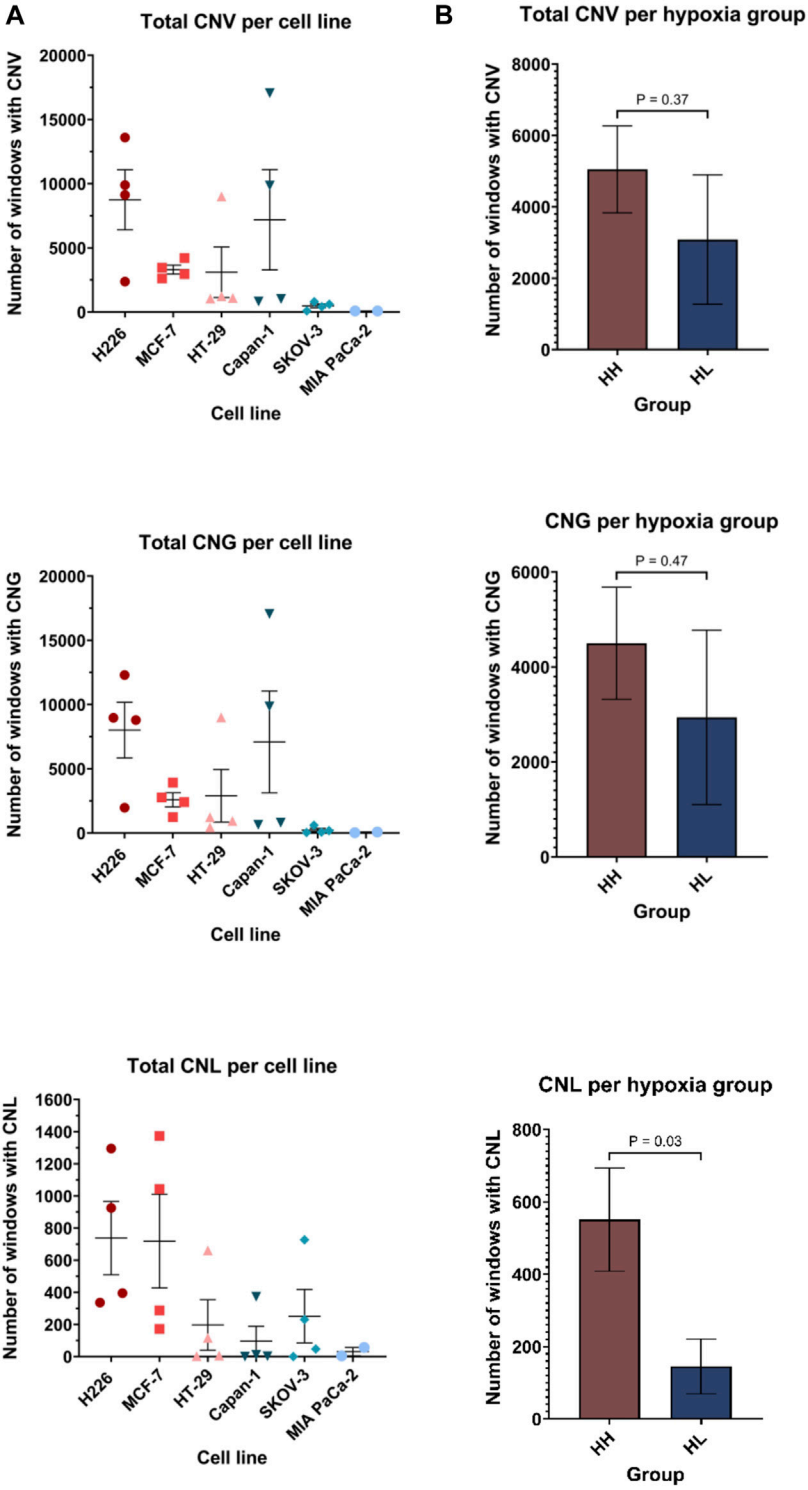
Copy number variation (CNV) profiles of cell lines in hypoxia. Number of windows with copy number variation (including both gains and losses), copy number gain (CNG) and copy number loss (CNL) per cell line **(A)** and per hypoxia group **(B)**. Each window is 50 kilo base pair (kbp). HH: hypoxia high; HL: hypoxia low. Statistical analysis based on unpaired two-tailed t-test with *p* ≤ 0.05 considered statistically significant.

The BAM files from whole exome sequencing were additionally run using Control-FREEK pipeline to delineate the total number of copy number variations (CNV), number of gains (CNG) and losses (CNL) for each cell line (Figure 7A). Regarding total CNV, both MIA PaCa-2 (84, 95%CI: 71.29–96.71) and SKOV-3 (480, 95%CI: −3.667-963.7) showed very low mean variation per a 50 kbp window, while H226 (8747, 95%CI: 1301–16192), MCF-7 (3306, 95%CI: 2202–4409) and Capan-1 (7188, 95%CI: 5244-19620) experienced higher mean CNV. Indeed, there was no significant difference between the total accumulated CNV in the HH *versus* HL groups (Figure 7B). Upon examination of CNG and CNL in specific, again a similar trend is observed for CNG, however, interestingly, in terms of CNL, there is a clear difference between the groups, where HH (551, 95%CI: 236.6–865.4) cell lines experienced on average significantly more CNL than the HL (145.3, 95%CI: −25.91-316.5) cell lines (*p* = 0.03). Therefore, while the effect of long passaging in hypoxia on chromosomal instability is cell-line dependent, cells that are more sensitive to hypoxia are more prone to experiencing copy number losses. These changes did not give rise to any discernable phenotypic changes, as cells presented with similar morphology when comparing early and late passages in hypoxia (Supplementary Figure S5).

### 3.7 Cell lines experiencing high genomic instability had enriched deregulation of DNA replication and repair

**FIGURE 8:**
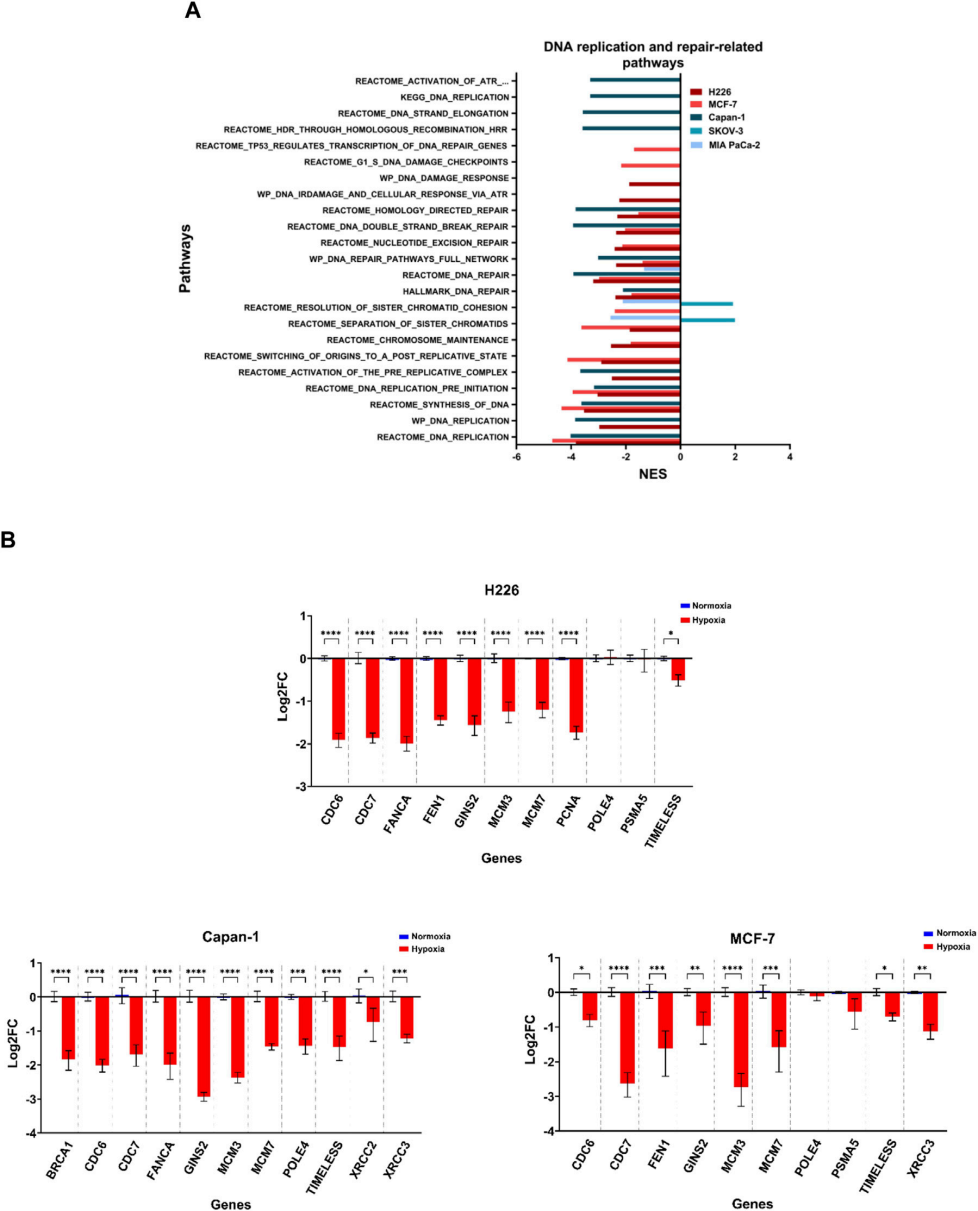
Downregulation of DNA replication and repair related pathways in hypoxia. **(A)** Significantly enriched pathways related to DNA replication or repair and chromosomal maintenance in the hypoxic (NES>0) or the normoxic (NES<0) condition based on gene set enrichment analysis. Only pathways with FDR q-value ≤0.05 are included. NES: normalized enrichment score. **(B)** Log2 fold change (FC) of genes related to the same pathways in H226, MCF-7 and Capan-1 in hypoxia *versus* normoxia. Statistical analysis based on ordinary one-way ANOVA with Sidak correction for multiple testing. *p* ≤ 0.05 considered statistically significant: *p = 0.032; **p = 0.0021; ***p = 0.0002; ****p < 0.0001.

Increased copy number variations and sequence variations could be a consequence of a reduction in DNA replication and repair, as well as aberrant chromosomal segregation. The 3 cell lines that showed the highest TMB levels, namely H226, MCF-7 and Capan-1, also displayed decreased enrichment in pathways related to DNA replication and repair in hypoxia (Figure 8; Supplementary File S3; Supplementary Tables S6, S7). In addition, a negative enrichment of pathways related to chromosome maintenance was noted for H226 and MCF-7 (Figure 8A; Supplementary File S3; Supplementary Table S8). Interestingly, H226, MCF-7 and Capan-1 also displayed cell-specific enrichment of DNA repair and replication related pathways (Figure 8A; Supplementary File S3; Supplementary Tables S9–S11). In particular, Wiki pathways of DNA IR damage and cellular response *via* ATR as well as DNA damage response were downregulated in H226; REACTOME pathways of G1-S DNA damage checkpoints and transcription coupled NER were downregulated in MCF-7; and REACTOME gene sets of HDR, activation of ATR in response to replication stress and DNA strand elongation, as well as KEGG DNA replication were downregulated in Capan-1. No effects on such pathways could be reported for HT-29 (Figure 8A; Supplementary File S3), while SKOV-3 showed a positive enrichment in hypoxia for sister chromatids separation and resolution-related gene sets (Figure 8A; Supplementary File S3;Supplementary Table S8). On the other hand, MIA PaCa-2 only displayed a negative enrichment in these pathways, as well as in one DNA repair-related gene set (Figure 8A; Supplementary File S3; Supplementary Table S6, S8). The inherent characteristics of the cell lines seems to be a stronger contributor to their response to hypoxia than their allotted hypoxia group, with the cells with the highest mutability concurrently displaying strongest enrichment for aberrant DNA damage response and repair, as well as replication stress.

### 3.8 Cell lines varied in their enrichment of immune-related pathways

**FIGURE 9:**
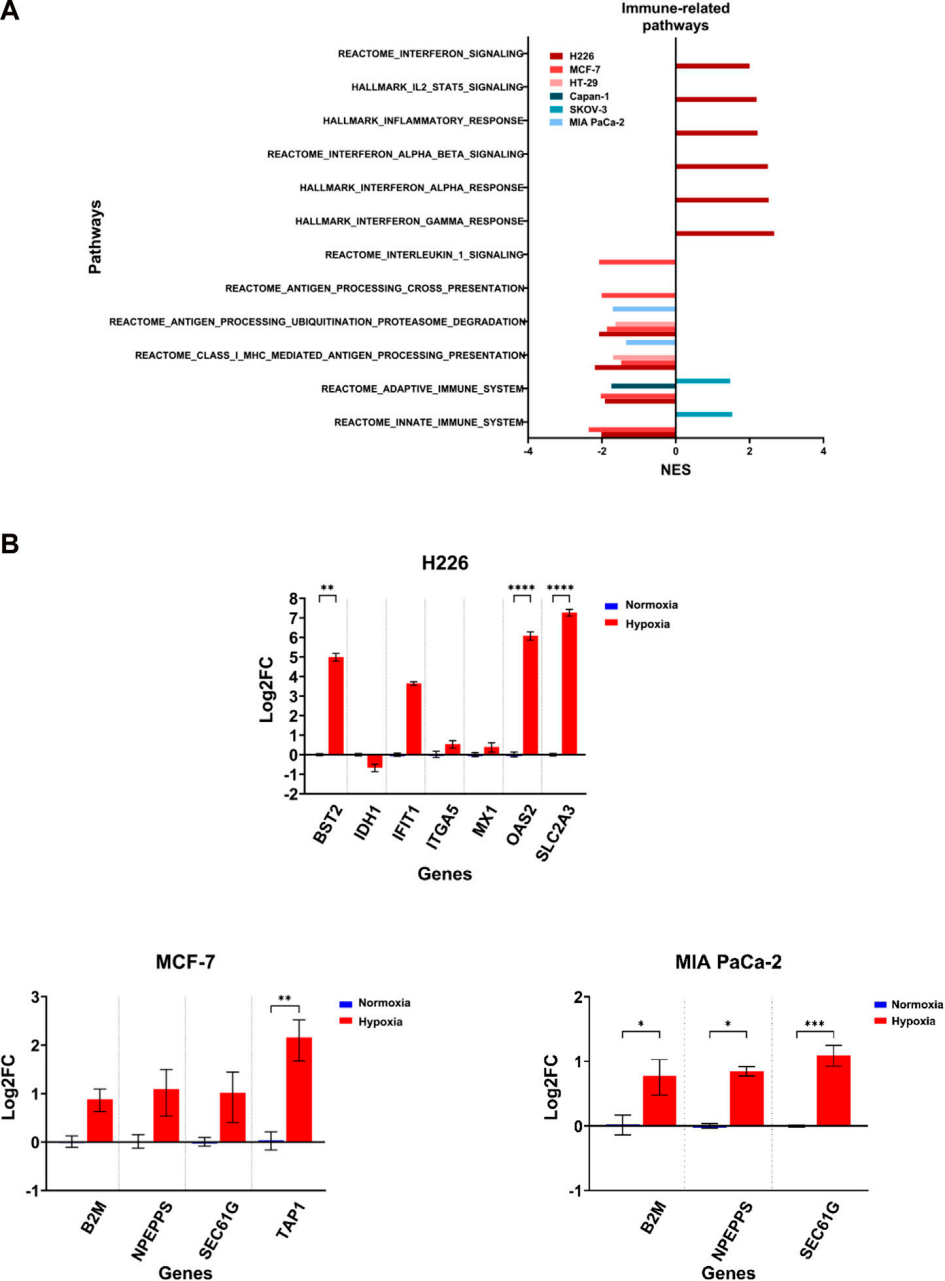
Differential enrichment of immune-related pathways. **(A)** Significantly enriched pathways related to immune response enriched in the hypoxic (NES>0) or the normoxic (NES<0) condition based on gene set enrichment analysis. Only pathways with FDR q-value ≤0.05 are included. NES: normalized enrichment score. **(B)** Log2 fold change of genes related to the same pathways in H226, MCF-7 and MIA PaCa-2 in hypoxia *versus* normoxia. Statistical analysis based on ordinary one-way ANOVA with Sidak correction for multiple testing. *p* ≤ 0.05 considered statistically significant: *p = 0.032; **p = 0.0021; ***p = 0.0002; ****p < 0.0001.

The induction of missense mutations, frameshift insertions and alternative splicing in hypoxia could give rise to proteins with altered amino acid sequences. The processing of such proteins by proteasomes could generate mutant peptides having higher affinity to the antigen presentation complexes and which could elicit a T-cell-mediated and tumor-specific response. Deregulation of any of these pathways involved in antigen presentation could influence the probability of eliciting a tumor-antagonizing immune response. By examining the enriched immune-related pathways, the downregulation of at least one pathway related to antigen processing and presentation or immune response could be observed across the cell lines in hypoxia, and which was especially evident in MCF-7 and H226 (Figure 9A). Furthermore, MCF-7 presented with additional downregulation of the Reactome Antigen Processing-Cross Presentation and Interleukin-1 Signaling pathways (Figure 9A; Supplementary Table S14). HT-29 and MIA PaCa-2 were also negatively enriched for pathways of antigen processing, ubiquitination and proteasome degradation as well as MHC class I-mediated antigen processing and presentation (Figure 9A). While several genes were upregulated (Figure 9B), looking closely at the affected genes in each pathway, it is apparent that a primary contributor to their negative enrichment is the downregulation of proteasomal subunit proteins (Supplementary Table S12). The exception to the negative enrichment was SKOV-3, which showed a positive enrichment of innate and adaptive immune pathways in hypoxia (Figure 9A). In addition, H226 displayed strong positive enrichment for pathways of inflammatory response (Figure 9; Supplementary Table S13). Therefore, chronic hypoxic stress can potentially impact immunogenicity of cancer cells in a cell-line dependent manner.

## 4 Discussion

Hypoxia is a common feature of solid tumors’ microenvironment, and multiple reports have established its role in driving various cancer hallmarks promoting malignancy (Vaupel and Mayer, 2014; Schito and Semenza, 2016; Abou Khouzam et al., 2020a; Abou Khouzam et al., 2020b; Abou Khouzam et al., 2022). Hypoxic levels are not constant throughout the tumor mass (Vaupel and Mayer, 2014; Bader et al., 2020; Hompland et al., 2021). In our study we subjected cancer cells to moderate levels of hypoxia (1% oxygen) in a chronic manner by maintaining them for twenty passages under hypoxic or normoxic (21% oxygen) conditions. By choosing a specific number of passages, as opposed to a defined time-period of incubation, we could level the field when dealing with different types of tumor cells having distinct doubling times. Despite biological relevance, how such hypoxic conditions affect tumor cells’ genomic and transcriptomic landscapes has not been investigated until now. Furthermore, inherent features of tumor cells could be impacting the sensitivity of their response to hypoxia. To delineate the effect of hypoxia on cells based on their inherent sensitivity to this condition, we first captured it using an eight-gene hypoxia signature that we previously derived from fourteen cancer cell lines representing six different tumor types (Abou Khouzam et al., 2021). The eight hypoxia-related genes were selected based on their consistent upregulation across all fourteen cell lines in hypoxia (Abou Khouzam et al., 2021). The fold change (FC) of expression for twelve tumor cell lines were included in this work considering 2 cell lines per solid cancer. The panel included breast, cervical, colon, lung, ovarian and pancreatic cancer cell lines, all of which are solid tumors with varying reported degrees of hypoxia (Bhandari et al., 2019). Two cell lines were chosen per cancer to prevent skewing of the results in favor of a specific tumor type, while keeping cell lines of diverse backgrounds to better represent the cancer subtypes. The FC values were then used to calculate a relative hypoxia score. From these scores, a group of more sensitive cells, characterized as hypoxia high (HH) could be distinguished from a group of more resistant cells, characterized as hypoxia low (HL). Subjecting these cells to chronic hypoxia followed by microarray transcriptome analysis and WES, revealed transcriptomic and genetic adaptations. This is the first comprehensive report on the impact of chronic hypoxia on tumor cells in which different cancer cell types were incorporated and grouped according to their hypoxic response.

In terms of the transcriptomic remodeling driven by hypoxia, there was a clear difference in the number of differentially expressed transcripts in the cell lines that were in the HH group, and thus more sensitive to hypoxia, than those in the HL group. In particular, there were 44 differentially expressed genes common to all 3 cell lines in the HH group, and only six in the HL group. Importantly among the 44 genes, 33 were consistently downregulated in hypoxia, and based on the functions of these genes in terms of mRNA or protein processing, synthesis, or transport (including *BOLA2*, *BOLA2B*, *DNAJC11*, *HARS1*, *HEATR3*), transcription or translation regulation (including *EIF4G2*, *MRPL12*, *POLR2K*), and biogenesis of ribosomes (*BYSL*, *EBNA1BP2*) and purines (*ATIC)*, it can be deduced that cells in the HH group mounted a bigger attenuation of key cellular processes when compared to those in the HL group. When considering results of pathway analysis at the cell-line level, the only commonly enriched pathway was that curated for hypoxia. This gene-set includes 200 genes reported to be upregulated in hypoxia and including HIF-1α targets. In addition, all the cell lines, except SKOV-3, showed significant downregulation of DNA/RNA/protein processing-, and cell cycle-related pathways. It seems like SKOV-3 showed bigger resistance to hypoxia than estimated based on the hypoxia score. Nonetheless, all the cell lines experienced a downregulation of pathways of metabolism. Interestingly, a common response to hypoxia could be determined that included sixteen genes. Three of these genes were downregulated, and among them two *IDH3A* and *COA7* are involved in metabolism. While IDH3A has been associated with stabilization and transactivation of HIF-1α (Zeng et al., 2015) this is the first report on the differential expression of *COA7* in hypoxia. The downregulation of these genes could be contributing to the decreased reliance of hypoxic cells on the oxidative respiratory chain performed through mitochondrial function. The remaining thirteen genes were consistently upregulated in all the cell lines, and included known HIF-1α activated genes, such as *PGK1* (Li et al., 1996) and *PDK1* (Kim et al., 2006) that play significant roles in the metabolic switch to glycolysis; *FUT11* (Cao et al., 2021; Ruan et al., 2021)and *KCTD11* (Leithner et al., 2014; Cao et al., 2021), involved in proliferation; *ADM* involved in invasion and angiogenesis (Keleg et al., 2007); *NDRG1* involved in apoptosis (Guo et al., 2020) and *P4HA1* playing a role in ECM remodeling (Gilkes et al., 2013). Of interest, the peptidylglycine alpha-amidating monooxygenase, *PAM*, has been reported as another oxygen sensing molecule that is activated in mild hypoxia and is involved in the C-terminal amidation of peptides that is necessary for their stability and biological function (Simpson et al., 2015). Herein we show that this gene is also activated in chronic hypoxia and could be involved in the downstream adaptation of cells to this condition as well. In addition, there was an upregulation of long non-coding RNA, including HIF1A antisense RNA 2 which has been implicated in mediating the transactivation of HIF-1α and glycolysis (Zheng et al., 2021). Therefore, we could report on both novel associations of genes with chronic hypoxia and highlight the implication of known hypoxia-activated genes that seem to be playing a crucial role in survival of the cells in such conditions and could present as important potential therapeutic targets to mitigate hypoxia.

The cellular adaptation to hypoxia has also been reported to direct the production of alternative transcripts by affecting alternative splicing processes in cancer cells, reportedly inducing thousands of alternative splicing events in breast (Han et al., 2017) liver (Sena et al., 2014) and head and neck (Brady et al., 2017) cancer cells. It has also been shown to promote splicing perturbations in both HIF-1-target and -nontarget genes (Sena et al., 2014). In our hypoxic conditions, we could again report a significant impact of hypoxia on alternative splicing, with the most common event being cassette exon. This is in accordance with a previous study reporting on lung and breast cancer cells exposed to chronic hypoxia, defined as 0.5% oxygen for 48 h (Fischer et al., 2020). Of interest, we could also identify four hypoxia-induced transcripts to be alternatively spliced in all cell lines, *PDK1*, *NDRG1*, *LDHA* and *SLC3A2*, however the expression of these alternate transcripts would need validation by RT-qPCR and how they could be contributing to cellular adaptation needs further assessment. From our data, we can conclude that alternative splicing is maintained in hypoxic cells even after long incubation periods, further underlining its importance in the cellular adaptation to this condition.

Thus far patient studies reporting on associations between hypoxia and genomic integrity have done so primarily using hypoxia gene signatures as surrogates for the degree of hypoxia in patient tumors (Bhandari et al., 2019; Bhandari et al., 2020; Hassan Venkatesh et al., 2021). The downside of such studies is that tumors are processed in bulk and analyzed sequencing data could be contaminated with data from non-tumor cells, such as immune and stromal cells. In addition, while they can provide strong correlation evidence between hypoxia and genomic instability they do not report on causation. *In vitro* studies on cancer cells offer a unique platform to directly analyze the impact of hypoxia on genomic instability, and while a number of studies have alluded to the involvement of hypoxia in mutation accumulation, they have done so through mutation reporter assays (reviewed in Luoto et al., 2013). In our previous work, we showed that hypoxia induces increased mutations in breast cancer cells (Hassan Venkatesh et al., 2020), herein we expanded the analysis onto a range of tumor cells deriving from cancers of the breast, lung, pancreas, ovary, and colon that portrayed high sensitivity or high resistance to hypoxia. We further escalated the hypoxic condition by subjecting the cells to long passaging in controlled hypoxic conditions of 1% oxygen. We could report that all investigated cells experienced an increase in TMB and there was a significantly higher mean TMB in HH cells compared to HL cells. One cell line in the HL subgroup, Capan-1, is known to have a *BRCA2* mutation and is homologous recombination deficient (HRD) (Abaji et al., 2005), which could explain its higher level of TMB when compared to other cells in the HL group. Hypoxia is a well-known factor that induces selection pressure in the TME (Graeber et al., 1996) and can induce mutability in genes (for example *TP53*). It is important to note that through our experiment, we cannot distinguish between the effect of passaging in hypoxia on accumulation of mutations and hypoxia alone as a genotoxic stress. This is relevant given the findings of Ben-David et al., which eloquently highlight the genetic heterogeneity and extensive clonal diversity that exists in cancer cell lines, wherein selection pressure could lead to genetic instability and genetic diversification (Ben-David et al., 2018). Regarding CNV, one study that looked at specific regions in chromosome 1 using FISH showed that hypoxia promoted site-specific copy number gains following 24 h at 1% oxygen in a non-transformed hTERT-immortalized retinal pigment epithelial 1 cell line, as well as other cell lines (Black et al., 2015). Nonetheless, despite *in vivo* evidence implicating hypoxia in chromosomal instability, the incorporation of whole exome sequencing to evaluate its effect on CNV *in vitro* has not been reported. In this work we find that cells within the HH group, as well as Capan-1, showed the highest mean number of 50 kbp windows with total CNV and copy number gains. Again, seeing this increase in Capan-1 could be due to its HRD status. Interestingly, copy number losses seemed to be favored in cells in the HH group, as well as the ovarian cancer cell line SKOV-3. Our results are in line with an *in vivo* study showing a significant positive association between the percentage of the genome copy-number gains and separately copy-number losses, with hypoxia (Bhandari et al., 2020). How these copy number changes are implicated in the adaptation of tumor cells to hypoxia requires further investigation.

Genomic instability could be due to replication stress and deregulation of DNA repair pathways, both of which have been reported by us (Hassan Venkatesh et al., 2020) and others (Bindra et al., 2005; Pires et al., 2010; Fanale et al., 2013; Chan et al., 2014; Lu et al., 2014; Scanlon and Glazer, 2014; Farias et al., 2018; Ng et al., 2018; Bhandari et al., 2020; Cowman et al., 2021) (reviewed in (Luoto et al., 2013; Begg and Tavassoli, 2020)), to be induced in hypoxia. The condition of hypoxia selected with respect to exposure time and the percentage of oxygen, plays a role when it came to the alteration of DNA repair pathways, namely MMR, HRR as well as non-homologous end joining (NHEJ) (Begg and Tavassoli, 2020); and while the deregulation of these pathways has been reported in multiple studies, the consideration of chronic hypoxia has been limited to a few days (Begg and Tavassoli, 2020). In our chronic hypoxia conditions and through gene set enrichment analysis of microarray data, a decrease in enrichment of pathways involved in DNA replication and repair as well as chromosomal stability was observed. These pathways were negatively enriched in the cells suffering from highest TMB and CNV, namely, H226, MCF-7 and Capan-1, which puts them forth as the potential mechanisms giving rise to genomic instability in these cells. Our results were strengthened by the significant downregulation of several DNA replication and repair genes by RT-qPCR. Among the downregulated pathways, were those of NER, double strand break-repair and homology-directed repair, and in Capan-1, HRR. This is in line with results from a recent study examining signatures of copy number variations in 8,006 TCGA tumors stratified based on gene-expression-derived hypoxia scores that showed hypoxia to be associated with HRD (Steele et al., 2022).

Through our analysis, three genes were found to be mutated in all analyzed samples, namely *FLG*, *GPRIN2* and *PCDHB5*. Based on literature findings (Wu and Maniatis, 1999; Duan et al., 2018; Khalilipour et al., 2018; Zhou et al., 2021; Chen et al., 2022), it is difficult to assess how these genes could be contributing to the adaptation to hypoxia and given that we could not observe changes in their expression levels as per the microarray data, it could be that their mutation is only a bystander effect. By checking the alteration frequency of the top ten mutated genes in each cell line with its matching tumor type in TCGA, we could report a very high concordance for all cell lines except SKOV-3. We suspect this to be due to a difference in the genetic background and histopathology of the tumor used to derive this cell lines (adenocarcinoma), compared to the tumor type present in TCGA (serous cystadenocarcinoma). *In vitro*, only a handful of the top ten mutated genes per cell line, were affected at the transcriptional level through alterations in expression levels or alternative splicing, or both. Among those, *BRIP1* was alternatively spliced in MCF-7 through intron retention, which could render this transcript inactive. *BRIP1* is involved in the Fanconi anemia DNA repair pathway (Fang et al., 2020) and its downregulation thus supports the increase in genomic instability observed for MCF-7. Another gene, *ADAMTS9*, a tumor suppressor (Lo et al., 2010), was found to be significantly downregulated and alternatively spliced through intron retention in the same cell line; while the tumor suppressor *ZMYND8*, and the oncogene *ZNF217* involved in tumor stemness and metastasis (Cohen et al., 2015; Li et al., 2016) were found to be downregulated and upregulated, respectively; a gene involved in the regulation of RNA processing (Federico et al., 2017), *INTS2*, was upregulated as well. How these genes could be contributing to pathogenesis in hypoxia has not yet been investigated and deserves further attention, especially since all these genes were additionally found to be more altered in more hypoxic breast cancers from the TCGA dataset compared to less hypoxic samples.

Hypoxia has been shown to hamper the immune response against tumor cells by supporting an acidic and nutrient deprived microenvironment that inhibits the cytolytic function of T-cells and natural killer (NK) cells, while promoting the inhibitory role of regulatory T-cells (Baginska et al., 2013; Berchem et al., 2016; Watson et al., 2021; Ziani et al., 2021). Furthermore, it has been reported that hypoxia can mediate immune escape by downregulating antigen presenting proteins (Sethumadhavan et al., 2017). We have previously shown that breast cancer cells cultured at 1% oxygen in intermittent (through oxygen cycling) and chronic (72 h) hypoxia showed increase in transcription of antigen processing and presentation genes. Similarly, herein we show that there is an increase in certain genes, such as *B2M* in MCF-7 and MIA PaCa-2, however the pathways themselves are negatively enriched in all cell lines, except SKOV-3. This general negative enrichment could be due to a strong downregulation of proteasome subunits. Indeed, in addition to being a protein degrader, the proteasome is also involved in producing antigenic peptides to be presented by MHC class I molecules (Tanaka, 2009). Of interest, H226 showed positive enrichment for various immune related signaling and response pathways; how these could be contributing to the overall immunogenicity of this cell line in chronic hypoxia requires deeper investigation.

This study provides a global view on the reactivity to hypoxia of different tumor cells with varying inherent sensitivity to the same condition. The choice of focusing on the three highest and three lowest scoring cell lines was to ensure the presence of more than 2 cell lines per group to carry out proper comparative analysis. It would be interesting to see how the findings vary if all the scored cell lines are analyzed. This step would be necessary to determine the robustness of the signature in classifying the cells as HH and HL, and validate the association seen for higher TMB in HH compared to HL. Furthermore, clonality analysis would be necessary for distinguishing between the consequence of high passaging in hypoxia on clonal selection *versus* hypoxia alone. One limitation of this work is that we did not directly compare the response of these cells in the given condition with other hypoxic conditions, such as acute or cycling hypoxia. This could have firmly underlined chronic hypoxia-specific processes. In addition, the issue faced with the sequencing of two MIA PaCa-2 samples meant we only had two hypoxia samples for this cell line instead of four, as with the other cell lines. Another limitation is that by not carrying out RNA sequencing, we are only capturing a fraction of the altered transcriptome in hypoxia. Functional assays implicating the roles of the reported genes in the adaptation to hypoxia would be required to confirm their relevance to chronic hypoxia. Finally, without neoantigen load data it is difficult to make a complete conclusion on the effect of hypoxia on immunogenicity across the investigated cells.

In conclusion we have shown that in chronic hypoxia cancer cells are reprogrammed to an indolent state, and yet they incur genomic instability in the form of high tumor mutational burden, mainly in the form of frameshift insertions, and copy number alterations. This could be due to the downregulation of pathways related to DNA damage response and repair, replication stress, as well as chromosomal maintenance. Of interest, cells that were ranked as being more sensitive to hypoxic stress accumulated a mean TMB that is significantly higher than the more resistant cells. They further experienced a higher rate of copy number loss. We also found that most genes altered *in vitro* in hypoxia were frequently mutated in patients with more hypoxic tumors, compared to those with less hypoxic ones, shedding light on their potential relevance to this condition. A global response to chronic hypoxia could also be observed, wherein there was an increase in alternative splicing of both HIF-1-target and non-target genes across cells, and we could report a common signature composed of 16 genes, suggesting the presence of a core response to chronic hypoxia. This core response included well-acknowledged hypoxia-response genes, such as *PGK1* and *NDRG1*, as well as much less investigated genes, including *COA7* and the oxygen sensing gene, *PAM*, whose roles in chronic hypoxia deserve further attention. Finally, our results also hint at the role of chronic hypoxia in depressing tumor cells’ inherent immunogenicity through the downregulation of antigen processing and presentation related pathways. Therefore, chronic hypoxia could influence the adaptation of tumor cells, enabling their survival by promoting metabolic reprogramming, modulating proliferation, and increasing genomic instability.

## Data Availability

The datasets presented in this study can be found in online repositories. The names of the repository/repositories and accession number(s) can be found below: https://www.ncbi.nlm.nih.gov/genbank/, PRJNA929085, https://www.ncbi.nlm.nih.gov/geo/, GSE222048.

## References

[B1] Abaji C. Cousineau I. Belmaaza A. (2005). BRCA2 regulates homologous recombination in response to DNA damage: Implications for genome stability and carcinogenesis. Cancer Res. 65 (10), 4117–4125. 10.1158/0008-5472.Can-04-3071 15899802

[B2] Abou Khouzam R. Brodaczewska K. Filipiak A. Zeinelabdin N. A. Buart S. Szczylik C. (2020a). Tumor hypoxia regulates immune escape/invasion: Influence on angiogenesis and potential impact of hypoxic biomarkers on cancer therapies. Front. Immunol. 11, 613114. 10.3389/fimmu.2020.613114 33552076 PMC7854546

[B3] Abou Khouzam R. Goutham H. V. Zaarour R. F. Chamseddine A. N. Francis A. Buart S. (2020b). Integrating tumor hypoxic stress in novel and more adaptable strategies for cancer immunotherapy. Semin. Cancer Biol. 65, 140–154. 10.1016/j.semcancer.2020.01.003 31927131

[B4] Abou Khouzam R. Rao S. P. Venkatesh G. H. Zeinelabdin N. A. Buart S. Meylan M. (2021). An eight-gene hypoxia signature predicts survival in pancreatic cancer and is associated with an immunosuppressed tumor microenvironment. Front. Immunol. 12, 680435. 10.3389/fimmu.2021.680435 34093582 PMC8173254

[B5] Abou Khouzam R. Zaarour R. F. Brodaczewska K. Azakir B. Venkatesh G. H. Thiery J. (2022). The effect of hypoxia and hypoxia-associated pathways in the regulation of antitumor response: Friends or foes? Front. Immunol. 13, 828875. 10.3389/fimmu.2022.828875 35211123 PMC8861358

[B6] Bader S. B. Dewhirst M. W. Hammond E. M. (2020). Cyclic hypoxia: An update on its characteristics, methods to measure it and biological implications in cancer. Cancers (Basel) 13 (1), 23. 10.3390/cancers13010023 33374581 PMC7793090

[B7] Baginska J. Viry E. Berchem G. Poli A. Noman M. Z. van Moer K. (2013). Granzyme B degradation by autophagy decreases tumor cell susceptibility to natural killer-mediated lysis under hypoxia. Proc. Natl. Acad. Sci. U. S. A. 110 (43), 17450–17455. 10.1073/pnas.1304790110 24101526 PMC3808626

[B8] Begg K. Tavassoli M. (2020). Inside the hypoxic tumour: Reprogramming of the DDR and radioresistance. Cell Death Discov. 6, 77. 10.1038/s41420-020-00311-0 32864165 PMC7434912

[B9] Ben-David U. Siranosian B. Ha G. Tang H. Oren Y. Hinohara K. (2018). Genetic and transcriptional evolution alters cancer cell line drug response. Nature 560 (7718), 325–330. 10.1038/s41586-018-0409-3 30089904 PMC6522222

[B10] Berchem G. Noman M. Z. Bosseler M. Paggetti J. Baconnais S. Le Cam E. (2016). Hypoxic tumor-derived microvesicles negatively regulate NK cell function by a mechanism involving TGF-β and miR23a transfer. Oncoimmunology 5 (4), e1062968. 10.1080/2162402x.2015.1062968 27141372 PMC4839360

[B11] Bhandari V. Hoey C. Liu L. Y. Lalonde E. Ray J. Livingstone J. (2019). Molecular landmarks of tumor hypoxia across cancer types. Nat. Genet. 51 (2), 308–318. 10.1038/s41588-018-0318-2 30643250

[B12] Bhandari V. Li C. H. Bristow R. G. Boutros P. C. Consortium P. (2020). Divergent mutational processes distinguish hypoxic and normoxic tumours. Nat. Commun. 11 (1), 737. 10.1038/s41467-019-14052-x 32024819 PMC7002770

[B13] Bindra R. S. Gibson S. L. Meng A. Westermark U. Jasin M. Pierce A. J. (2005). Hypoxia-induced down-regulation of BRCA1 expression by E2Fs. Cancer Res. 65 (24), 11597–11604. 10.1158/0008-5472.Can-05-2119 16357170

[B14] Black J. C. Atabakhsh E. Kim J. Biette K. M. Van Rechem C. Ladd B. (2015). Hypoxia drives transient site-specific copy gain and drug-resistant gene expression. Genes Dev. 29 (10), 1018–1031. 10.1101/gad.259796.115 25995187 PMC4441050

[B15] Brady L. K. Wang H. Radens C. M. Bi Y. Radovich M. Maity A. (2017). Transcriptome analysis of hypoxic cancer cells uncovers intron retention in EIF2B5 as a mechanism to inhibit translation. PLoS Biol. 15 (9), e2002623. 10.1371/journal.pbio.2002623 28961236 PMC5636171

[B16] Cao W. Zeng Z. Pan R. Wu H. Zhang X. Chen H. (2021). Hypoxia-related gene FUT11 promotes pancreatic cancer progression by maintaining the stability of PDK1. Front. Oncol. 11, 675991. 10.3389/fonc.2021.675991 34221996 PMC8247946

[B17] Chan N. Ali M. McCallum G. P. Kumareswaran R. Koritzinsky M. Wouters B. G. (2014). Hypoxia provokes base excision repair changes and a repair-deficient, mutator phenotype in colorectal cancer cells. Mol. Cancer Res. 12 (10), 1407–1415. 10.1158/1541-7786.Mcr-14-0246 25030372

[B18] Chen H. Zhao L. Liu J. Zhou H. Wang X. Fang X. (2022). Bioinformatic analyzes of the association between upregulated expression of *JUN* gene *via APOBEC*-induced *FLG* gene mutation and prognosis of cervical cancer. Front. Med. (Lausanne) 9, 815450. 10.3389/fmed.2022.815450 35510248 PMC9058067

[B19] Cohen P. A. Donini C. F. Nguyen N. T. Lincet H. Vendrell J. A. (2015). The dark side of ZNF217, a key regulator of tumorigenesis with powerful biomarker value. Oncotarget 6 (39), 41566–41581. 10.18632/oncotarget.5893 26431164 PMC4747174

[B20] Cowman S. Pizer B. Sée V. (2021). Downregulation of both mismatch repair and non-homologous end-joining pathways in hypoxic brain tumour cell lines. PeerJ 9, e11275. 10.7717/peerj.11275 33986995 PMC8092103

[B21] Duan C. Wang H. Chen Y. Chu P. Xing T. Gao C. (2018). Whole exome sequencing reveals novel somatic alterations in neuroblastoma patients with chemotherapy. Cancer Cell Int. 18, 21. 10.1186/s12935-018-0521-3 29467591 PMC5816515

[B22] Fanale D. Bazan V. Caruso S. Castiglia M. Bronte G. Rolfo C. (2013). Hypoxia and human genome stability: Downregulation of BRCA2 expression in breast cancer cell lines. Biomed. Res. Int. 2013, 746858. 10.1155/2013/746858 24171172 PMC3793298

[B23] Fang C. B. Wu H. T. Zhang M. L. Liu J. Zhang G. J. (2020). Fanconi anemia pathway: Mechanisms of breast cancer predisposition development and potential therapeutic targets. Front. Cell Dev. Biol. 8, 160. 10.3389/fcell.2020.00160 32300589 PMC7142266

[B24] Farias J. G. Zepeda A. Castillo R. Figueroa E. Ademoyero O. T. Pulgar V. M. (2018). Chronic hypobaric hypoxia diminishes the expression of base excision repair OGG1 enzymes in spermatozoa. Andrologia 50 (2), e12876. 10.1111/and.12876 28758699

[B25] Federico A. Rienzo M. Abbondanza C. Costa V. Ciccodicola A. Casamassimi A. (2017). Pan-cancer mutational and transcriptional analysis of the integrator complex. Int. J. Mol. Sci. 18 (5), 936. 10.3390/ijms18050936 28468258 PMC5454849

[B26] Fischer S. Di Liddo A. Taylor K. Gerhardus J. S. Sobczak K. Zarnack K. (2020). Muscleblind-like 2 controls the hypoxia response of cancer cells. RNA 26 (5), 648–663. 10.1261/rna.073353.119 32127384 PMC7161353

[B27] Germano G. Lamba S. Rospo G. Barault L. Magrì A. Maione F. (2017). Inactivation of DNA repair triggers neoantigen generation and impairs tumour growth. Nature 552 (7683), 116–120. 10.1038/nature24673 29186113

[B28] Gilkes D. M. Bajpai S. Chaturvedi P. Wirtz D. Semenza G. L. (2013). Hypoxia-inducible factor 1 (HIF-1) promotes extracellular matrix remodeling under hypoxic conditions by inducing P4HA1, P4HA2, and PLOD2 expression in fibroblasts. J. Biol. Chem. 288 (15), 10819–10829. 10.1074/jbc.M112.442939 23423382 PMC3624462

[B29] Graeber T. G. Osmanian C. Jacks T. Housman D. E. Koch C. J. Lowe S. W. (1996). Hypoxia-mediated selection of cells with diminished apoptotic potential in solid tumours. Nature 379 (6560), 88–91. 10.1038/379088a0 8538748

[B30] Guo D. D. Xie K. F. Luo X. J. (2020). Hypoxia-induced elevated NDRG1 mediates apoptosis through reprograming mitochondrial fission in HCC. Gene 741, 144552. 10.1016/j.gene.2020.144552 32165297

[B31] Han J. Li J. Ho J. C. Chia G. S. Kato H. Jha S. (2017). Hypoxia is a key driver of alternative splicing in human breast cancer cells. Sci. Rep. 7 (1), 4108. 10.1038/s41598-017-04333-0 28642487 PMC5481333

[B32] Hassan Venkatesh G. Abou Khouzam R. Shaaban Moustafa Elsayed W. Ahmed Zeinelabdin N. Terry S. Chouaib S. (2021). Tumor hypoxia: An important regulator of tumor progression or a potential modulator of tumor immunogenicity? Oncoimmunology 10 (1), 1974233. 10.1080/2162402x.2021.1974233 34595058 PMC8477925

[B33] Hassan Venkatesh G. Bravo P. Shaaban Moustafa Elsayed W. Amirtharaj F. Wojtas B. Abou Khouzam R. (2020). Hypoxia increases mutational load of breast cancer cells through frameshift mutations. Oncoimmunology 9 (1), 1750750. 10.1080/2162402x.2020.1750750 32363122 PMC7185205

[B34] Hompland T. Fjeldbo C. S. Lyng H. (2021). Tumor hypoxia as a barrier in cancer therapy: Why levels matter. Cancers (Basel) 13 (3), 499. 10.3390/cancers13030499 33525508 PMC7866096

[B35] Kaplan A. R. Glazer P. M. (2020). Impact of hypoxia on DNA repair and genome integrity. Mutagenesis 35 (1), 61–68. 10.1093/mutage/gez019 31282537 PMC7317153

[B36] Keleg S. Kayed H. Jiang X. Penzel R. Giese T. Büchler M. W. (2007). Adrenomedullin is induced by hypoxia and enhances pancreatic cancer cell invasion. Int. J. Cancer 121 (1), 21–32. 10.1002/ijc.22596 17290391

[B37] Khalilipour N. Baranova A. Jebelli A. Heravi-Moussavi A. Bruskin S. Abbaszadegan M. R. (2018). Familial Esophageal Squamous Cell Carcinoma with damaging rare/germline mutations in KCNJ12/KCNJ18 and GPRIN2 genes. Cancer Genet. 221, 46–52. 10.1016/j.cancergen.2017.11.011 29405996

[B38] Kim J. W. Tchernyshyov I. Semenza G. L. Dang C. V. (2006). HIF-1-mediated expression of pyruvate dehydrogenase kinase: A metabolic switch required for cellular adaptation to hypoxia. Cell Metab. 3 (3), 177–185. 10.1016/j.cmet.2006.02.002 16517405

[B39] Leithner K. Wohlkoenig C. Stacher E. Lindenmann J. Hofmann N. A. Gallé B. (2014). Hypoxia increases membrane metallo-endopeptidase expression in a novel lung cancer *ex vivo* model - role of tumor stroma cells. BMC Cancer 14, 40. 10.1186/1471-2407-14-40 24460801 PMC3905926

[B40] Li H. Ko H. P. Whitlock J. P. (1996). Induction of phosphoglycerate kinase 1 gene expression by hypoxia. Roles of Arnt and HIF1alpha. J. Biol. Chem. 271 (35), 21262–21267. 10.1074/jbc.271.35.21262 8702901

[B41] Li N. Li Y. Lv J. Zheng X. Wen H. Shen H. (2016). ZMYND8 reads the dual histone mark H3K4me1-H3K14ac to antagonize the expression of metastasis-linked genes. Mol. Cell 63 (3), 470–484. 10.1016/j.molcel.2016.06.035 27477906 PMC4975651

[B42] Livak K. J. Schmittgen T. D. (2001). Analysis of relative gene expression data using real-time quantitative PCR and the 2(-Delta Delta C(T)) Method. Methods 25 (4), 402–408. 10.1006/meth.2001.1262 11846609

[B43] Lo P. H. Lung H. L. Cheung A. K. Apte S. S. Chan K. W. Kwong F. M. (2010). Extracellular protease ADAMTS9 suppresses esophageal and nasopharyngeal carcinoma tumor formation by inhibiting angiogenesis. Cancer Res. 70 (13), 5567–5576. 10.1158/0008-5472.Can-09-4510 20551050 PMC2896444

[B44] Lu Y. Wajapeyee N. Turker M. S. Glazer P. M. (2014). Silencing of the DNA mismatch repair gene MLH1 induced by hypoxic stress in a pathway dependent on the histone demethylase LSD1. Cell Rep. 8 (2), 501–513. 10.1016/j.celrep.2014.06.035 25043185 PMC4111985

[B45] Luoto K. R. Kumareswaran R. Bristow R. G. (2013). Tumor hypoxia as a driving force in genetic instability. Genome Integr. 4 (1), 5. 10.1186/2041-9414-4-5 24152759 PMC4016142

[B46] Ng N. Purshouse K. Foskolou I. P. Olcina M. M. Hammond E. M. (2018). Challenges to DNA replication in hypoxic conditions. FEBS J. 285 (9), 1563–1571. 10.1111/febs.14377 29288533

[B47] Pires I. M. Bencokova Z. Milani M. Folkes L. K. Li J. L. Stratford M. R. (2010). Effects of acute versus chronic hypoxia on DNA damage responses and genomic instability. Cancer Res. 70 (3), 925–935. 10.1158/0008-5472.Can-09-2715 20103649 PMC2923514

[B48] Rospo G. Lorenzato A. Amirouchene-Angelozzi N. Magrì A. Cancelliere C. Corti G. (2019). Evolving neoantigen profiles in colorectal cancers with DNA repair defects. Genome Med. 11 (1), 42. 10.1186/s13073-019-0654-6 31253177 PMC6599263

[B49] Ruan W. Yang Y. Yu Q. Huang T. Wang Y. Hua L. (2021). FUT11 is a target gene of HIF1α that promotes the progression of hepatocellular carcinoma. Cell Biol. Int. 45 (11), 2275–2286. 10.1002/cbin.11675 34288238

[B50] Scanlon S. E. Glazer P. M. (2014). Hypoxic stress facilitates acute activation and chronic downregulation of fanconi anemia proteins. Mol. Cancer Res. 12 (7), 1016–1028. 10.1158/1541-7786.Mcr-13-0628 24688021 PMC4101147

[B51] Schito L. Semenza G. L. (2016). Hypoxia-inducible factors: Master regulators of cancer progression. Trends Cancer 2 (12), 758–770. 10.1016/j.trecan.2016.10.016 28741521

[B52] Schreiber R. D. Old L. J. Smyth M. J. (2011). Cancer immunoediting: Integrating immunity's roles in cancer suppression and promotion. Science 331 (6024), 1565–1570. 10.1126/science.1203486 21436444

[B53] Sena J. A. Wang L. Heasley L. E. Hu C. J. (2014). Hypoxia regulates alternative splicing of HIF and non-HIF target genes. Mol. Cancer Res. 12 (9), 1233–1243. 10.1158/1541-7786.Mcr-14-0149 24850901 PMC4163527

[B54] Sethumadhavan S. Silva M. Philbrook P. Nguyen T. Hatfield S. M. Ohta A. (2017). Hypoxia and hypoxia-inducible factor (HIF) downregulate antigen-presenting MHC class I molecules limiting tumor cell recognition by T cells. PLoS One 12 (11), e0187314. 10.1371/journal.pone.0187314 29155844 PMC5695785

[B55] Simpson P. D. Eipper B. A. Katz M. J. Gandara L. Wappner P. Fischer R. (2015). Striking oxygen sensitivity of the peptidylglycine α-amidating monooxygenase (PAM) in neuroendocrine cells. J. Biol. Chem. 290 (41), 24891–24901. 10.1074/jbc.M115.667246 26296884 PMC4598998

[B56] Steele C. D. Abbasi A. Islam S. M. A. Bowes A. L. Khandekar A. Haase K. (2022). Signatures of copy number alterations in human cancer. Nature 606 (7916), 984–991. 10.1038/s41586-022-04738-6 35705804 PMC9242861

[B57] Tanaka K. (2009). The proteasome: Overview of structure and functions. Proc. Jpn. Acad. Ser. B Phys. Biol. Sci. 85 (1), 12–36. 10.2183/pjab.85.12 PMC3524306 19145068

[B58] Tang M. Bolderson E. O'Byrne K. J. Richard D. J. (2021). Tumor hypoxia drives genomic instability. Front. Cell Dev. Biol. 9, 626229. 10.3389/fcell.2021.626229 33796526 PMC8007910

[B59] Vaupel P. Mayer A. (2014). Hypoxia in tumors: Pathogenesis-related classification, characterization of hypoxia subtypes, and associated biological and clinical implications. Adv. Exp. Med. Biol. 812, 19–24. 10.1007/978-1-4939-0620-8_3 24729210

[B60] Watson M. J. Vignali P. D. A. Mullett S. J. Overacre-Delgoffe A. E. Peralta R. M. Grebinoski S. (2021). Metabolic support of tumour-infiltrating regulatory T cells by lactic acid. Nature 591 (7851), 645–651. 10.1038/s41586-020-03045-2 33589820 PMC7990682

[B61] Wu Q. Maniatis T. (1999). A striking organization of a large family of human neural cadherin-like cell adhesion genes. Cell 97 (6), 779–790. 10.1016/s0092-8674(00)80789-8 10380929

[B62] Zaarour R. F. Sharda M. Azakir B. Hassan Venkatesh G. Abou Khouzam R. Rifath A. (2022). Genomic analysis of waterpipe smoke-induced lung tumor autophagy and plasticity. Int. J. Mol. Sci. 23 (12), 6848. 10.3390/ijms23126848 35743294 PMC9225041

[B63] Zeng L. Morinibu A. Kobayashi M. Zhu Y. Wang X. Goto Y. (2015). Aberrant IDH3α expression promotes malignant tumor growth by inducing HIF-1-mediated metabolic reprogramming and angiogenesis. Oncogene 34 (36), 4758–4766. 10.1038/onc.2014.411 25531325

[B64] Zheng F. Chen J. Zhang X. Wang Z. Lin X. Huang H. (2021). The HIF-1α antisense long non-coding RNA drives a positive feedback loop of HIF-1α mediated transactivation and glycolysis. Nat. Commun. 12 (1), 1341. 10.1038/s41467-021-21535-3 33637716 PMC7910558

[B65] Zhou W. Guan W. Zhou Y. Rao Y. Ji X. Li J. (2021). Weighted genes associated with the progression of retinoblastoma: Evidence from bioinformatic analysis. Exp. Eye Res. 211, 108730. 10.1016/j.exer.2021.108730 34419445

[B66] Ziani L. Buart S. Chouaib S. Thiery J. (2021). Hypoxia increases melanoma-associated fibroblasts immunosuppressive potential and inhibitory effect on T cell-mediated cytotoxicity. Oncoimmunology 10 (1), 1950953. 10.1080/2162402x.2021.1950953 34367731 PMC8312612

