# Supplementary material for: Chronic hypoxia is associated with transcriptomic reprogramming and increased genomic instability in cancer cells
Source: Front Cell Dev Biol. 2023 Mar 9;11:1095419. doi: 10.3389/fcell.2023.1095419 (PMC10033758; doi:10.3389/fcell.2023.1095419)

Supplementary Figure 1 The distribution of differentially expressed transcript clusters across the hypoxia groups and cell lines. TC: transcript cluster; HH: hypoxia high; HL: hypoxia low.

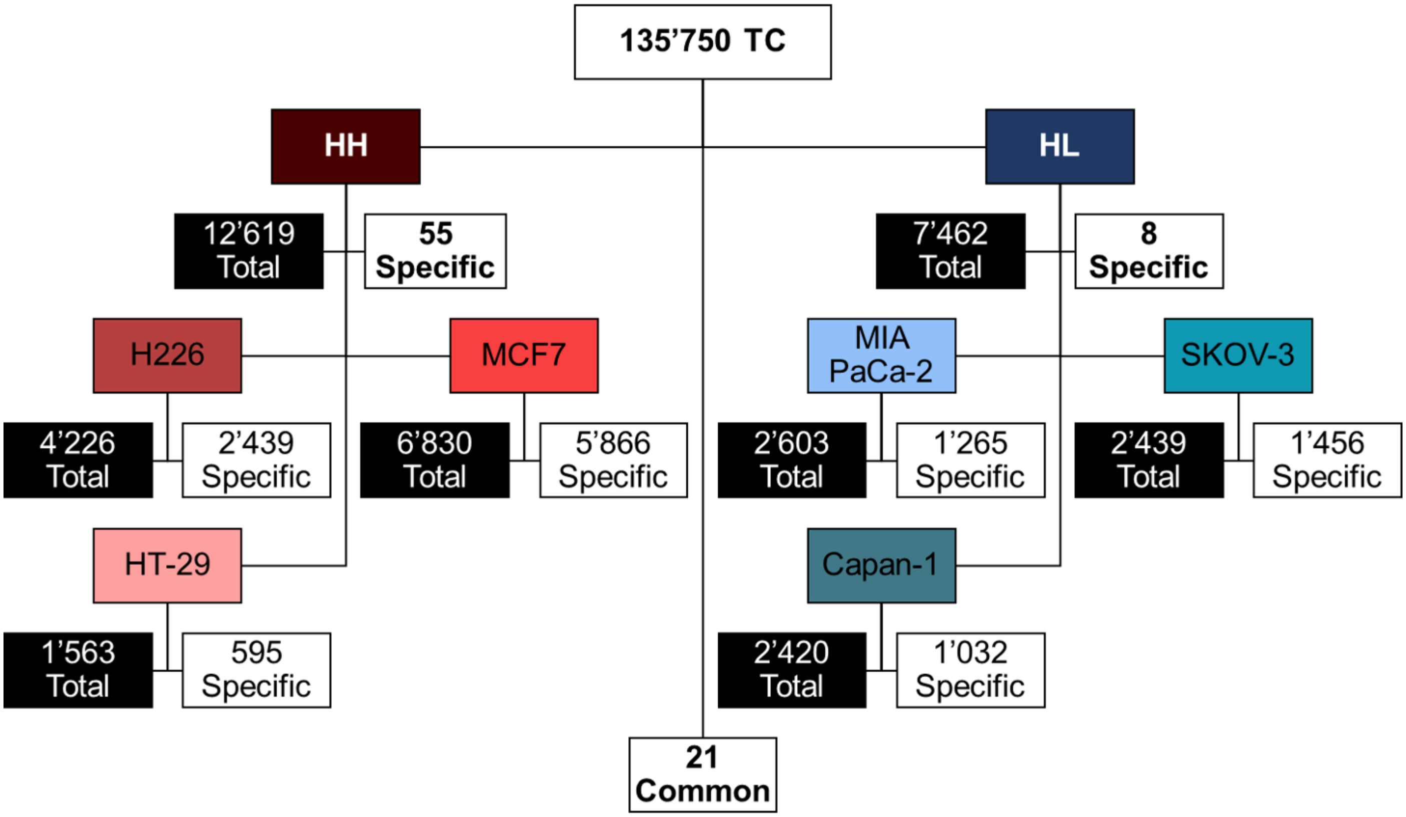

Supplementary Figure 2 Differentially expressed genes validated by RT-qPCR. Log2 fold change (FC) of genes in hypoxia (H) versus normoxia (N) of hypoxia high group-specific genes **(A)**; hypoxia low group-specific genes **(B)** and genes common to all cell lines **(C)**. Statistical analysis based on ordinary one-way ANOVA with Sidak correction for multiple testing. P-value ≤ 0.05 considered statistically significant: * P = 0.032; ** P = 0.0021; *** P = 0.0002; **** P < 0.0001 (A,B). HIF1-AS2 expression levels for HT-29 not shown in (C) since it was not expressed in normoxia (Cq values >> 30) therefore FC could not be calculated.

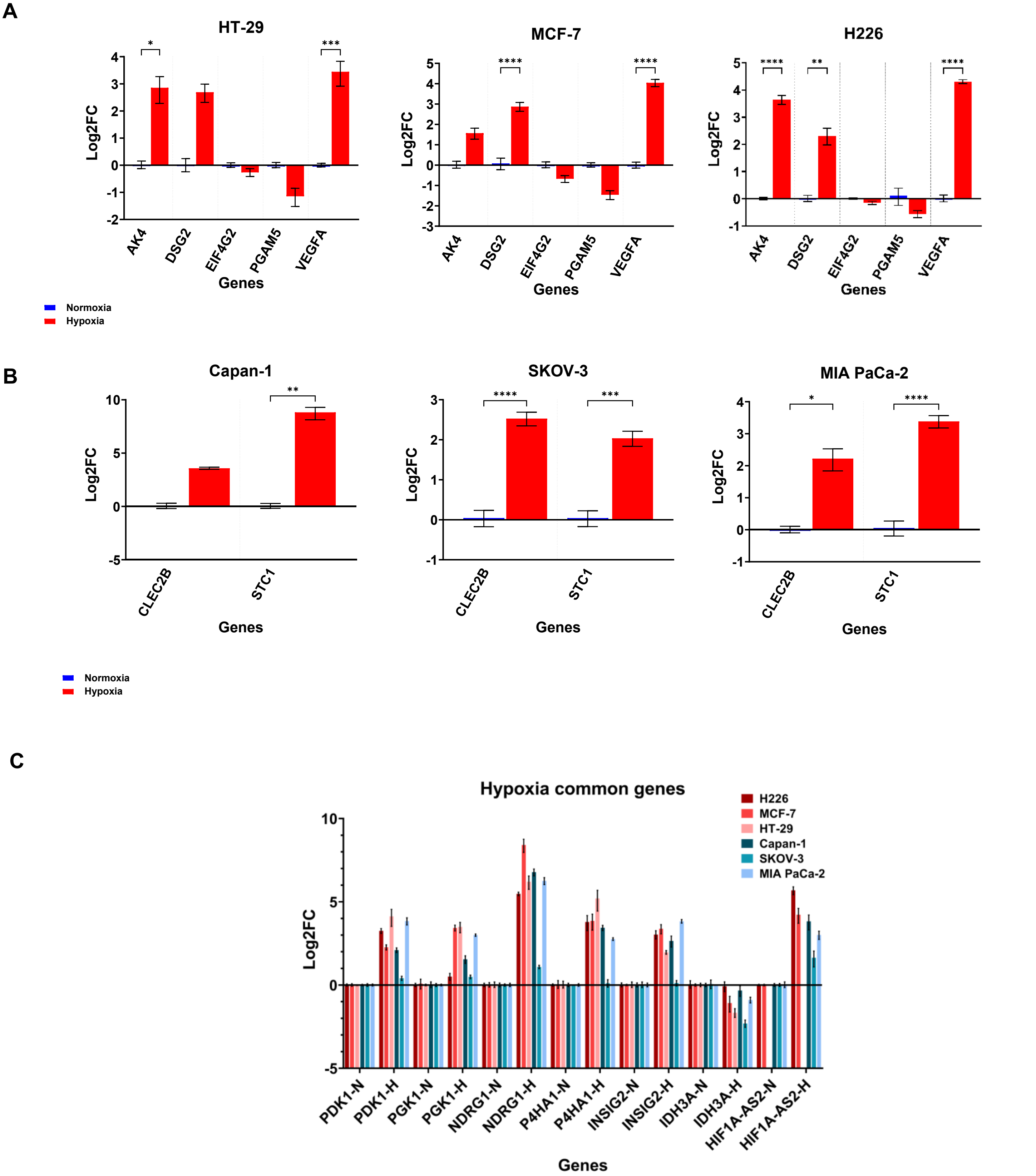

Supplementary Figure 3 Summary of alternatively spliced transcripts and events in hypoxia. Number of alternatively spliced transcript clusters (TCs) per cell line **(A)** and per hypoxia group **(B)**. **(C)** Total number of events and the number of each type of alternative splicing event occurring per cell line. Statistical analysis based on unpaired two-tailed t-test with P-value ≤ 0.05 considered statistically significant.

A

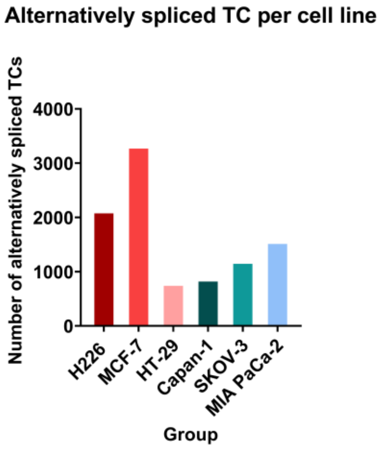

B

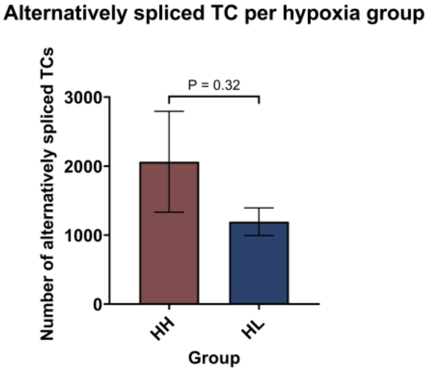

C

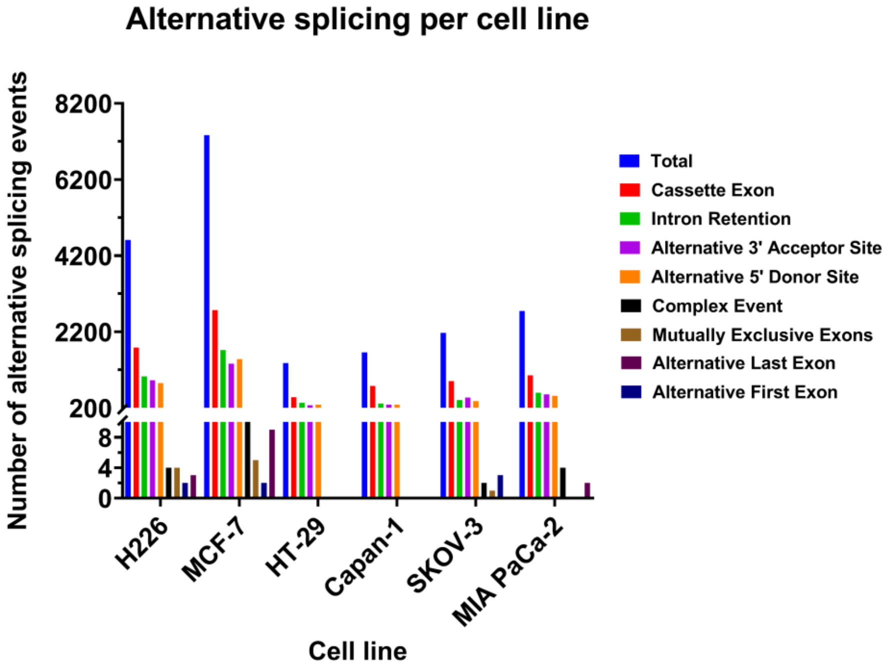

Supplementary Figure 4 Significantly enriched cell-line specific pathways. Enrichment in the hypoxic (NES>0) or the normoxic (NES<0) condition based on gene set enrichment analysis. Only pathways with FDR q-value ≤ 0.05 are included. NES: normalized enrichment score.

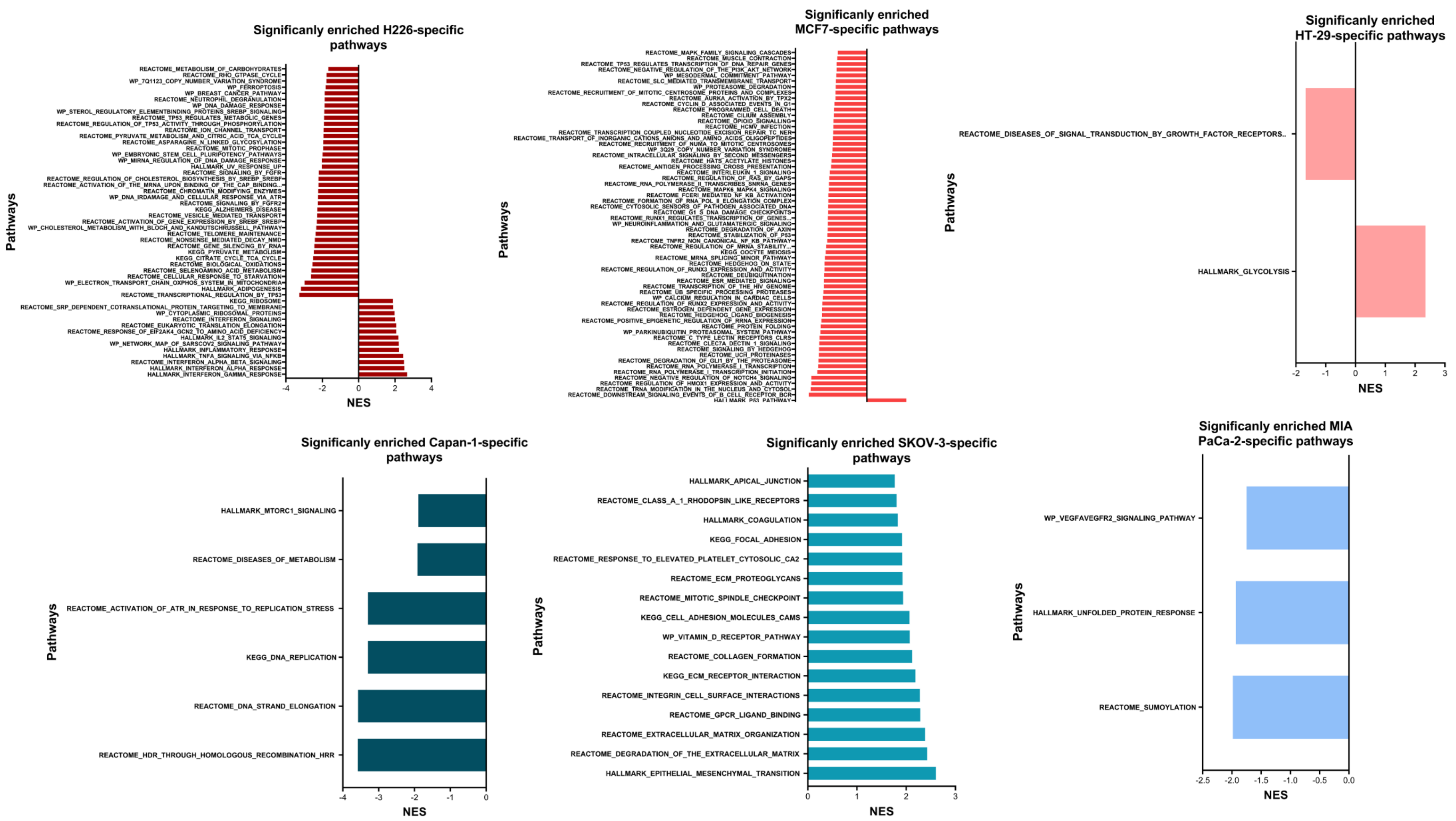

Supplementary Figure 5 Cellular morphology at early versus late passage in hypoxia. 10X magnification.

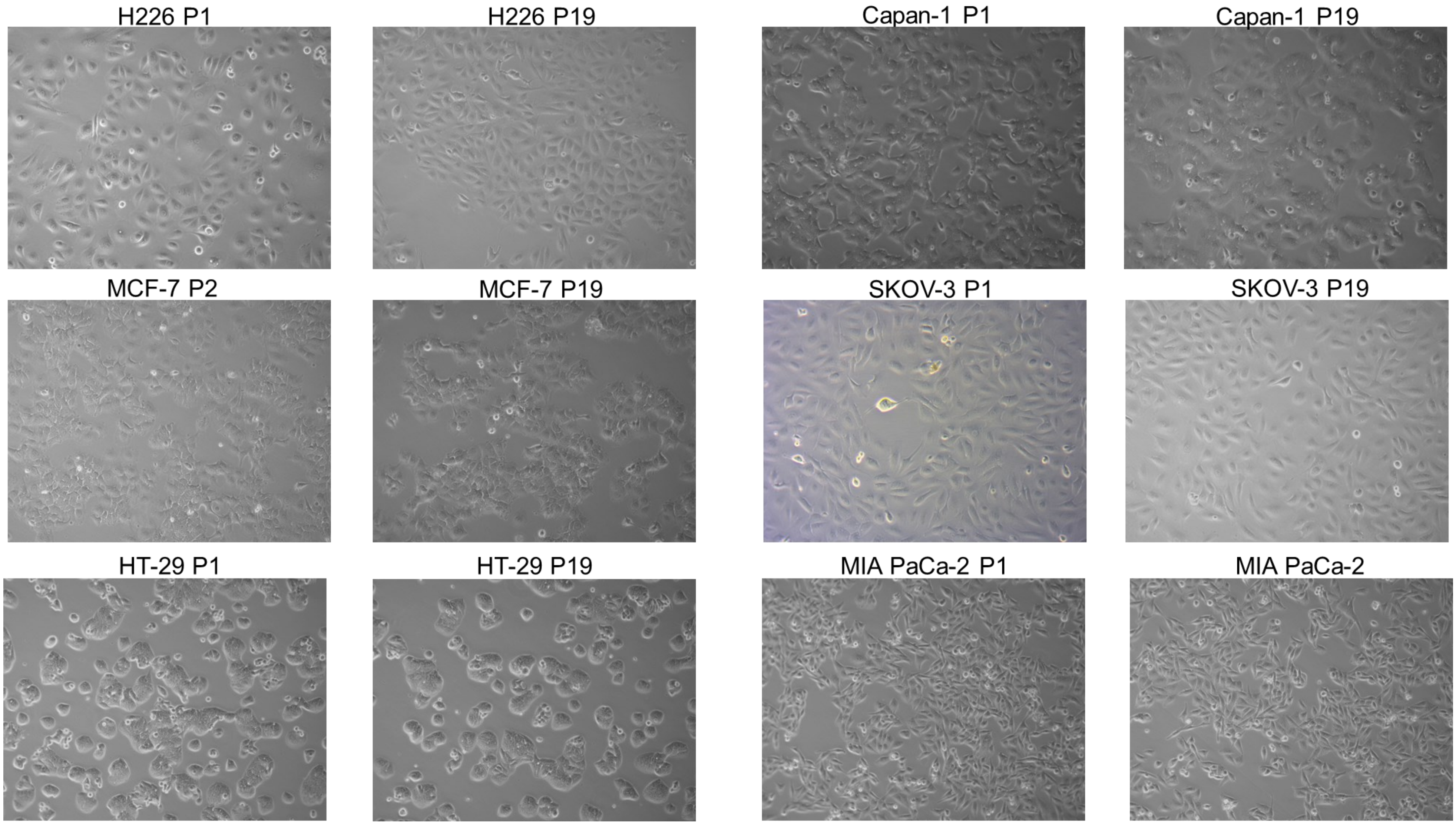

Supplement: Supplementary file 1 [file DataSheet2.PDF]
