# Supplementary material for: Chronic hypoxia is associated with transcriptomic reprogramming and increased genomic instability in cancer cells
Source: Front Cell Dev Biol. 2023 Mar 9;11:1095419. doi: 10.3389/fcell.2023.1095419 (PMC10033758; doi:10.3389/fcell.2023.1095419)
Supplement: Supplementary file 4 [file DataSheet1.PDF]

Supplementary Table 1. Whole exome sequencing sample information

S.n	Sample name	Type	Biological replicate number[a]	Technical replicate number[b]	Analysis status
1	Run20_CP1_Capan1normoxiaEXP1_33.vcf	normoxia	1	1	Yes
2	Run20_CP2_Capan1normoxiaEXP2_34.vcf	normoxia	2	1	Yes (processed: data cleaning)
3	Run21_CP1_Capan1normoxiaEXP1_39.vcf	normoxia	1	2	Yes (processed: data cleaning)
4	Run21_CP3_Capan1hypoxiaEXP1P20I_35.vcf	hypoxia	1	1	yes
5	Run22_CP2_Capan1normoxiaEXP2_40.vcf	normoxia	2	2	yes
6	Run22_CP4_Capan1hypoxiaEXP1P20II_36.vcf	hypoxia	1	2	Yes (Processed: data cleaning)
7	Run27_CP5_Capan1hypoxiaEXP2P20I_17.vcf	hypoxia	2	1	Yes (Processed: data cleaning)
8	Run27_CP6_Capan1hypoxiaEXP2P20II_18.vcf	hypoxia	2	2	Yes
9	Run12_H1_H226normoxiaEXP1_17.vcf	normoxia	1	1	Yes
10	Run12_H2_H226normoxiaEXP2_18.vcf	normoxia	2	1	Yes
11	Run13_H3_H226hypoxiaEXP1P20I_19.vcf	hypoxia	1	1	Yes
12	Run13_H5_H226hypoxiaEXP2P20I_21.vcf	hypoxia	2	1	Yes
13	Run14_H4_H226hypoxiaEXP1P20II_20.vcf	hypoxia	1	2	Yes
14	Run14_H6_H226hypoxiaEXP2P20II_22.vcf	hypoxia	2	2	Yes
15	Run15_H1_H226normoxiaEXP1_23.vcf	normoxia	1	2	Yes
16	Run15_H2_H226normoxiaEXP2_24.vcf	normoxia	2	2	Yes
17	Run23_HT1_HT29normoxiaEXP1_41.vcf	normoxia	1	1	Yes
18	Run23_HT3_HT29hypoxiaEXP1P20I_43.vcf	hypoxia	1	1	Yes
19	Run24_HT2_HT29normoxiaEXP2_42.vcf	normoxia	2	1	Yes
20	Run24_HT4_HT29hypoxiaEXP1P20II_44.vcf	hypoxia	1	2	Yes
21	Run25_HT1_HT29normoxiaEXP1_47.vcf	normoxia	1	2	Yes
22	Run25_HT5_HT29hypoxiaEXP2P20I_45.vcf	hypoxia	2	1	Yes
23	Run26_HT2_HT29normoxiaEXP2_48.vcf	normoxia	2	2	Yes
24	Run26_HT6_HT29hypoxiaEXP2P20II_46.vcf	hypoxia	2	2	Yes
25	Run16_MC1_MCF7normoxiaEXP1_25.vcf	normoxia	1	1	Yes
26	Run16_MC2_MCF7normoxiaEXP2_26.vcf	normoxia	2	1	Yes
27	Run17_MC3_MCF7hypoxiaEXP1P20I_27.vcf	hypoxia	1	1	Yes
28	Run17_MC5_MCF7hypoxiaEXP2P20I_29.vcf	hypoxia	1	2	Yes
29	Run18_MC4_MCF7hypoxiaEXP1P20II_28.vcf	hypoxia	2	1	Yes
30	Run18_MC6_MCF7hypoxiaEXP2P20II_30.vcf	hypoxia	2	2	Yes
31	Run19_MC1_MCF7normoxiaEXP1_31.vcf	normoxia	1	2	Yes
32	Run19_MC2_MCF7normoxiaEXP2_32.vcf	normoxia	2	2	Yes
33	Run3_MIA1_MiaPaca2normoxiaEXP1_17.vcf	normoxia	1	1	Yes
34	Run3_MIA3_MiaPaca2hypoxiaEXP1P20I_19.vcf	hypoxia	1	1	Yes
35	Run4_MIA2_MiaPaca2normoxiaEXP2_18.vcf	normoxia	2	1	Yes

36	Run5_MIA5_MiaPaca2hypoxiaEXP1P20I_21.vcf	hypoxia	2	1	Yes
37	Run10_SK2_SKOV3normoxiaEXP2_28.vcf	normoxia	2	1	Yes
38	Run10_SK3_SKOV3hypoxiaEXP1P20I_29.vcf	hypoxia	1	1	Yes
39	Run11_SK5_SKOV3hypoxiaEXP2P20I_31.vcf	hypoxia	2	1	Yes
40	Run11_SK6_SKOV3hypoxiaEXP2P20II_32.vcf	hypoxia	2	2	Yes
41	Run6_SK1_SKOV3normoxiaEXP1_23.vcf	normoxia	1	1	Yes
42	Run6_SK2_SKOV3normoxiaEXP2_24.vcf	normoxia	2	2	Yes
43	Run9_SK1_SKOV3normoxiaEXP1_27.vcf	normoxia	1	2	Yes
44	Run9_SK4_SKOV3hypoxiaEXP1P20II_30.vcf	hypoxia	1	2	Yes

[a] Biological replicate number indicates whether sample came from Experiment 1 or Experiment 2.

[b] Technical replicate number indicates replicate number from the same Experiment

Supplementary Table 2. Primer sequences used in RT-qPCR

SN	Gene Symbol	Forward Primer (5'-3' direction)	Reverse Primer (5'-3' direction)	Product Size (base pairs)
1	*ACTB*	TCCTTCCTGGGCATGGAGT	AGCACTGTGTTGGCGTACAG	104
2	*AK4*	TGGTCTCCAGCATCTCTCCA	TGCCATCTCACCAACTTCGG	76
3	*B2M*	GAGGCTATCCAGCGTACTCCA	CGGCAGGCATACTCATCTTTT	248
4	*BST2*	TCTCCTGCAACAAGAGCTGA	TCTTCTCAGTCGCTCCACCT	214
5	*BRCA1*	GAAACCGTGCCAAAAGACTTC	CCAAGGTTAGAGAGTTGGACAC	88
6	*CDC6*	AAAGCACTGGATGTTTGCAGGA	GACCAACCCTCTTGGGAATCA	127
7	*CDC7*	GTGCCCCAATCAAACTACAGCAA	GGCCAAAGCAGTTAAATCATCACT	115
8	*CLEC2B*	GTTCCACTCAACATGCCGAC	TGCCATCTTCAGTCCAATCCA	107
9	*DSG2*	GCACCACCTGAAGACAAGGT	CCCACCTTCCATCCATCTCG	172
10	*EIF4G2*	ATAACAGCAACTTCCCAAAAGGCA	TTGGGTTATATCTTCTTTCCAAGCC	110
11	*FANCA*	GCTGCTGACTGTGGAGCAGAG	GCCTTGTACGTGAAGATGCCA	165
12	*FEN1*	CCCGTGTATGTCTTTGATGG	TCACCAGCCGCTTAGTGAAT	154
13	*GINS2*	CCAATGCCCAGCCCTTACTAC	CTGCCTTCGGGATGTTGTCT	76
14	*IDH1*	TGTGGTAGAGATGCAAGGAGA	TTGGTGACTTGGTCGTTGGTG	147
15	*IDH3A*	ATTTTTGAGTCGGTTCATGGGAC	AGCAAAACACGCAGCCTCAA	147
16	*IFIT1*	CCTAATTTACAGCAACCATGAGGA	TGCTCCAGACTATCCTTGACCT	178
17	*INSIG2*	TAATGCGGTGTGTAGCAGTCT	GTCCAATGGATAGTGCAGCCA	108
18	*ITGA5*	GGCTTCAACTTAGACGCGGAG	TGGCTGGTATTAGCCTTGGGT	140
19	*MCM3*	GTGGAGTTGAGTGAATCCAGGT	AGGCGATTCATGCCGATTGA	95
20	*MCM7*	GGGCTCCAGATTCATCAAAT	ATACCAGTGACGCTGACGTG	153
21	*MX1*	GGAGGCACTGTCAGGAGTTG	TCCTGGTAACTGACCTTGCC	123
22	*NDRG1*	CGCCAGCACATTGTGAATGAC	TTTGAGTTGCACTCCACCACG	191
23	*NPEPPS*	GTGAGGCAGGCGACTAATCAG	GTTCCCGTACCTGTTTGCAG	173
24	*OAS2*	GTTGACTTCTCCCAACCTGGA	GTGAAGAGTGGTGCAGGCAG	72
25	*P4HA1*	ACCCCATTTTGACTTTGCACG	CCTGTCCCCAGCTCTTTGAAA	60
26	*PCNA*	GCGTGAACCTCACCAGTATGT	TCTTCGGCCCTTAGTGTAATGAT	79
27	*PDK1*	ACCAGGACAGCCAATACAAG	CCTCGGTCACTCATCTTCAC	183
28	*PGAM5*	GCTACATCGTGTGCAGAGC	GATCTTGTCGGGAGGCATGA	155
29	*PGK1*	GACCTAATGTCCAAAGCTGAGAA	CAGCAGGTATGCCAGAAGCC	127
30	*POLE4*	GGCAGATCCCGACGTGAC	CAGCTTCTATTGCATTATCCAAGTC	164
31	*PSMA5*	TGCCATGTCTCGTCCCTTTG	TTTGTTGCATTCAGCTTCTCCT	249
32	*SEC61G*	ATGGCAACAGCAATAGGAT	ACACTTGTTCACCAATCTCT	145
33	*SLC2A3*	ATCTATGCCACCATCGGCG	CTGCCCTTTCCACCAGAAATAGAGA	76
34	*STC1*	TGAAGCCATCACTGAGGTCG	TCGGACAAGTCTGTTATAGTATCTG	70
35	*TAP1*	GGTCCTCTCCTCTCTTGGGG	CGGCTGAGCCATCTTGTAGA	83
36	*TIMELESS*	ATCTTGCCAAATGGAGCGGA	GGAGCGGGATAGAAAAGCCT	196
37	*XRCC2*	TCACCTGTGCATGGTGATATT	TTCCAGGCCACCTTCTGATT	117
38	*XRCC3*	AGGCTGGAGCCGTCTACAT	ATCTGGCTGCCAAATCGGAG	136

Supplementary Table 3. Group specific and common differentially expressed genes

SN	Gene Symbol	Gene Description	Biological Function
**Hypoxia High-Specific Genes**			
1	*ACP6*	acid phosphatase 6, lysophosphatidic	Lipid metabolism
2	*AIMP2*	aminoacyl tRNA synthetase complex interacting multifunctional protein 2	Apoptosis
3	*AK4*	adenylate kinase 4	Stress response
4	*ANAPC15*	anaphase promoting complex subunit 15	Cell cycle
5	*ATIC*	5-aminoimidazole-4-carboxamide ribonucleotide formyltransferase/IMP cyclohydrolase	Purine biosynthesis
6	*ATP8B1*	ATPase phospholipid transporting 8B1	Lipid transport
7	*AURKAIP1*	aurora kinase A interacting protein 1	Ribonucleoprotein
8	*BOLA2*	bolA family member 2	Cytosolic iron-sulfur (Fe-S) cluster assembly factor
9	*BOLA2B*	bolA family member 2B	Cytosolic iron-sulfur (Fe-S) cluster assembly factor
10	*BYSL*	bystin like	Ribosome biogenesis
11	*C11orf98*	chromosome 11 open reading frame 98	Uncharacterized protein
12	*DNAJC11*	DnaJ heat shock protein family (Hsp40) member C11	Chaperone
13	*DSG2*	desmoglein 2	Cell adhesion
14	*EBNA1BP2*	EBNA1 binding protein 2	Ribosome biogenesis
15	*EIF4G2*	eukaryotic translation initiation factor 4 gamma 2	Translation regulation
16	*GAPDHP63*	glyceraldehyde 3 phosphate dehydrogenase pseudogene 63	Pseudogene
17	*HARS1*	histidyl-tRNA synthetase 1	Protein biosynthesis
18	*HEATR3*	HEAT repeat containing 3	Ribosomal protein transport
19	*HNRNPM*	heterogeneous nuclear ribonucleoprotein M	mRNA processing
20	*HSPH1*	heat shock protein family H (Hsp110) member 1	Stress response
21	*INSIG1*	insulin induced gene 1	Cholesterol metabolism
22	*MPHOSPH6*	M-phase phosphoprotein 6	RNA-binding
23	*MRPL12*	mitochondrial ribosomal protein L12	Mitochondrial translation
24	*MRPL4*	mitochondrial ribosomal protein L4	Ribonucleoprotein
25	*NDUFA4L2*	NDUFA4 mitochondrial complex associated like 2	Mitochondrial respiratory chain complex IV
26	*PAK1IP1*	PAK1 interacting protein 1	Signal transduction inhibitor
27	*PDE12*	phosphodiesterase 12	mRNA processing
28	*PGAM5*	PGAM family member 5, mitochondrial serine/threonine protein phosphatase	Necrosis
29	*PHF23*	PHD finger protein 23	Autophagy
30	*PIGM*	phosphatidylinositol glycan anchor biosynthesis class M	Glycosylphosphatidylinositol (GPI)-anchor biosynthesis
31	*PLD1*	phospholipase D1	Lipid metabolism
32	*POLR2K*	RNA polymerase II, I and III subunit K	Transcription
33	*PPP1R10*	protein phosphatase 1 regulatory subunit 10	Protein phosphatase inhibitor
34	*PRKAR1B*	protein kinase cAMP-dependent type I regulatory subunit beta	cAMP signaling
35	*PRSS8*	serine protease 8	Proteolytic enzyme

36	*PUS7*	pseudouridine synthase 7	tRNA processing
37	*RANP1*	RAN pseudogene 1	Pseudogene
38	*SEH1L*	SEH1 like nucleoporin	Cell cycle
39	*SLC25A19*	solute carrier family 25 member 19	Mitochondrial transporter
40	*SORL1*	sortilin related receptor 1	Endocytosis
41	*TOMM5*	translocase of outer mitochondrial membrane 5	Protein transport
42	*TRIM27*	tripartite motif containing 27	Transcription regulation
43	*TRMT6*	tRNA methyltransferase 6 non-catalytic subunit	tRNA processing
44	*VEGFA*	vascular endothelial growth factor A	Angiogenesis
**Hypoxia Low-Specific Genes**			
1	*APOBEC3F*	apolipoprotein B mRNA editing enzyme catalytic subunit 3F	Cytidine deaminase
2	*APOBEC3G*	apolipoprotein B mRNA editing enzyme catalytic subunit 3G	Cytidine deaminase
3	*CLEC2B*	C-type lectin domain family 2 member B	Cell adhesion
4	*LOC105372290*	long intergenic non-protein coding RNA 1764	Long noncoding RNA
5	*PSAT1P1*	phosphoserine aminotransferase 1 pseudogene 1	Pseudogene
6	*STC1*	stanniocalcin 1	Hormone
**Common Genes**			
1	*ADM*	adrenomedullin	Angiogenesis
2	*ARRDC3*	arrestin domain containing 3	Energy expenditure
3	*COA7*	Cytochrome C Oxidase Assembly Factor 7	Metabolism
4	*FUT11*	fucosyltransferase 11	Proliferation
5	*HIF1A-AS2*	HIF1A antisense RNA 2	Long non-coding
6	*IDH3A*	isocitrate dehydrogenase (NAD(+)) 3 catalytic subunit alpha	Metabolism
7	*INSIG2*	insulin induced gene 2	Fatty acid synthesis
8	*KCTD11*	potassium channel tetramerization domain containing 11	Proliferation
9	*LOC154761*	family with sequence similarity 115, member C pseudogene	Long non-coding
10	*NDRG1*	N-myc downstream regulated 1	Apoptosis
11	*P4HA1*	prolyl 4-hydroxylase subunit alpha 1	ECM remodeling
12	*PAM*	peptidylglycine alpha-amidating monooxygenase	Neuropeptide amidation
13	*PDK1*	pyruvate dehydrogenase kinase 1	Glycolysis
14	*PFKFB4*	6-phosphofructo-2-kinase/fructose-2,6-biphosphatase 4	Glycolysis
15	*PGK1*	phosphoglycerate kinase 1	Glycolysis
16	*SLC35F6*	Solute Carrier Family 35 Member F6	Transmembrane transporter

Supplementary Table 4. Top ten mutated genes that are significantly differentially expressed

Cell line	Probe ID	Gene Symbol	Gene Description	Fold Change	P-val	FDR P-val
**H226**						
	TC1900007512.hg.1	*ZNF431*	zinc finger protein 431; vomeronasal 1 receptor 82 pseudogene	-2.1	0.0017	0.0453
**MCF-7**						
	TC0300011391.hg.1	*ADAMTS9*	ADAM metallopeptidase with thrombospondin type 1 motif 9	-2.37	6.19E-05	0.0025
	TC1700011305.hg.1	*HEATR6*	HEAT repeat containing 6	2.16	0.0004	0.0097
	TC1700011366.hg.1	*INTS2*	integrator complex subunit 2	2.65	0.003	0.047
	TC2000007581.hg.1	*SULF2*	Memczak2013 ANTISENSE, coding, INTERNAL, intronic best transcript NM_001161841	3.5	0.0004	0.0111
	TC2000009292.hg.1	*ZMYND8*	zinc finger, MYND-type containing 8	-5.49	1.49E-07	1.93E-05
	TC2000009504.hg.1	*ZNF217*	zinc finger protein 217	2.37	1.63E-05	0.0009

Supplementary Table 5. Top ten mutated genes that are significantly alternatively spliced

Cell line	Probe ID	Gene Symbol	Gene Description	Exon Splicing Index	Exon P-val	Exon FDR P-val	Exon Event Name	Exon Event Score	Exon Splicing Index	Exon P-val
**MCF-7**										
	TC0300011391.hg.1	*ADAMTS9*	ADAM metallopeptidase with thrombospondin type 1 motif 9	6.04	0.0004	0.0142	Intron Retention	0.43	6.04	0.0004
	TC1700011364.hg.1	*BRIP1*	BRCA1 interacting protein C-terminal helicase 1	10.43	3.49E-05	0.0023	Intron Retention	0.48	10.43	3.49E-05
	TC1700011366.hg.1	*INTS2*	integrator complex subunit 2	3.35	0.0009	0.0236	Alternative 3' Acceptor Site	0.22	3.35	0.0009
	TC2000009317.hg.1	*SULF2*	sulfatase 2	6.08	1.42E-06	0.0002	Intron Retention	0.56	6.08	1.42E-06
	TC2000009292.hg.1	*ZMYND8*	zinc finger, MYND-type containing 8	6.64	0.0006	0.0178	Intron Retention	0.44	6.64	0.0006
**MIA PaCa-2**										
	TC0700013375.hg.1	*ZNF273*	zinc finger protein 273	5.19	0.0005	0.0265	Cassette Exon	0.24	5.19	0.0005

Supplementary Table 6. Fold change of DNA repair-related genes based on pathway analysis across cell lines

Pathways	Gene Symbol	Gene Description	H226	MCF-7	HT-29	Capan-1	SKOV-3	MIA PaCa-2
Hallmark DNA Repair	*ADRM1*	ADRM1 26S proteasome ubiquitin receptor	#N/A	-2.91	#N/A	#N/A	#N/A	#N/A
	AGO4	argonaute RISC component 4	#N/A	2.26	#N/A	#N/A	#N/A	#N/A
	AK1	adenylate kinase 1	#N/A	-3.39	#N/A	#N/A	-4.1	#N/A
	ALYREF	Aly/REF export factor	-3.16	-5.38	#N/A	#N/A	#N/A	#N/A
	APRT	adenine phosphoribosyltransferase	-3.64	-3.82	#N/A	-2.08	#N/A	#N/A
	ARL6IP1	ADP ribosylation factor like GTPase 6 interacting protein 1	#N/A	2.27	#N/A	#N/A	#N/A	-2.58
	BCAM	basal cell adhesion molecule (Lutheran blood group)	-5.29	2.39	#N/A	4.2	#N/A	#N/A
	BCAP31	B cell receptor associated protein 31	#N/A	-2.28	#N/A	#N/A	#N/A	#N/A
	BOLA2	bolA family member 2	-3.86	-4.04	-2.31	#N/A	#N/A	#N/A
	BRF2	BRF2 RNA polymerase III transcription initiation factor subunit	#N/A	4.31	#N/A	#N/A	#N/A	#N/A
	CDA	cytidine deaminase	#N/A	#N/A	18.17	14.09	#N/A	80.22
	CMPK2	cytidine/uridine monophosphate kinase 2	2.93	#N/A	#N/A	#N/A	#N/A	#N/A
	CSTF3	cleavage stimulation factor subunit 3	-2.01	-2.58	#N/A	#N/A	#N/A	#N/A
	DGCR8	DGCR8 microprocessor complex subunit	#N/A	2.06	#N/A	#N/A	#N/A	#N/A
	DUT	deoxyuridine triphosphatase	#N/A	-5.3	#N/A	-3.04	#N/A	#N/A
	EDF1	endothelial differentiation related factor 1	#N/A	-3.34	#N/A	-2.14	#N/A	#N/A
	GPX4	glutathione peroxidase 4	#N/A	-3.68	#N/A	#N/A	#N/A	#N/A
	GTF2A2	general transcription factor IIA subunit 2	#N/A	#N/A	-2.97	#N/A	#N/A	-2.05
	GUK1	guanylate kinase 1	#N/A	#N/A	#N/A	2.11	#N/A	2.37

HPRT1	hypoxanthine phosphoribosyltransferase 1	#N/A	-4.6	#N/A	#N/A	#N/A	#N/A
IMPDH2	inosine monophosphate dehydrogenase 2	-2.4	#N/A	#N/A	#N/A	#N/A	#N/A
ITPA	inosine triphosphatase	-2.24	#N/A	#N/A	#N/A	#N/A	#N/A
MRPL40	mitochondrial ribosomal protein L40	#N/A	#N/A	-2.41	#N/A	#N/A	#N/A
NCBP2	nuclear cap binding protein subunit 2	#N/A	#N/A	#N/A	#N/A	-3.35	#N/A
NELFCD	negative elongation factor complex member C/D	-2.86	-2.2	#N/A	#N/A	#N/A	#N/A
NME1	NME/NM23 nucleoside diphosphate kinase 1	-2.63	#N/A	#N/A	#N/A	#N/A	#N/A
NME4	NME/NM23 nucleoside diphosphate kinase 4	-2.49	#N/A	2.65	#N/A	#N/A	#N/A
NT5C	5', 3'-nucleotidase, cytosolic	#N/A	-2.19	#N/A	#N/A	#N/A	#N/A
NUDT9	nudix hydrolase 9	-2.57	#N/A	#N/A	#N/A	#N/A	#N/A
PNP	purine nucleoside phosphorylase	#N/A	-3.66	-3.08	#N/A	#N/A	-5.2
POLR1C	RNA polymerase I and III subunit C	-2.5	-2.57	-5.2	#N/A	#N/A	-2.46
POLR3C	RNA polymerase III subunit C	#N/A	-3.6	#N/A	-2.72	#N/A	#N/A
PRIM1	DNA primase subunit 1	-5.17	#N/A	#N/A	-4.76	#N/A	#N/A
RAE1	ribonucleic acid export 1	-2.11	#N/A	#N/A	#N/A	#N/A	#N/A
RALA	RAS like proto-oncogene A	#N/A	2.7	#N/A	#N/A	#N/A	#N/A
RNMT	RNA guanine-7 methyltransferase	2.57	7.62	#N/A	2.72	#N/A	5.1
RRM2B	ribonucleotide reductase regulatory TP53 inducible subunit M2B	#N/A	8.97	#N/A	#N/A	#N/A	#N/A
SDCBP	syndecan binding protein	-2.45	7.3	#N/A	#N/A	#N/A	4.08
SRSF6	serine and arginine rich splicing factor 6	-2.82	-3.32	-3.84	#N/A	-2.4	-11.31
SSRP1	structure specific recognition protein 1	-2.21	#N/A	#N/A	-2.81	#N/A	#N/A

	STX3	syntaxin 3	-3.63	#N/A	#N/A	#N/A	-4.05	#N/A
	SUPT5H	SPT5 homolog, DSIF elongation factor subunit	#N/A	2.56	#N/A	#N/A	#N/A	#N/A
	SURF1	SURF1 cytochrome c oxidase assembly factor	#N/A	-2.94	#N/A	#N/A	#N/A	#N/A
	TAF10	TATA-box binding protein associated factor 10	-2.24	#N/A	#N/A	#N/A	#N/A	#N/A
	UMPS	uridine monophosphate synthetase	-2.54	-3.99	#N/A	#N/A	#N/A	-2.48
	USP11	ubiquitin specific peptidase 11	#N/A	#N/A	#N/A	-2.93	#N/A	#N/A
	VPS28	VPS28 subunit of ESCRT-I	#N/A	-2.77	#N/A	#N/A	#N/A	#N/A
	VPS37B	VPS37B subunit of ESCRT-I	#N/A	#N/A	#N/A	-2.56	#N/A	#N/A
	ZWINT	ZW10 interacting kinetochore protein	#N/A	#N/A	#N/A	#N/A	6.49	#N/A
**WP DNA Repair Pathways Full Network**	*GTF2H2C*	GTF2H2 family member C	#N/A	-3.65	#N/A	#N/A	#N/A	#N/A
	RAP1A	RAP1A, member of RAS oncogene family	#N/A	-2.01	#N/A	#N/A	#N/A	#N/A
**Reactome DNA Repair**	*ALKBH2*	alkB homolog 2, alpha-ketoglutarate dependent dioxygenase	#N/A	-2.44	#N/A	#N/A	#N/A	#N/A
	ALKBH5	alkB homolog 5, RNA demethylase	#N/A	3.24	#N/A	#N/A	#N/A	3.62
	ASCC1	activating signal cointegrator 1 complex subunit 1	#N/A	#N/A	#N/A	#N/A	#N/A	2.57
	DCLRE1B	DNA cross-link repair 1B	#N/A	#N/A	#N/A	-2.67	2.2	#N/A
	DTL	denticleless E3 ubiquitin protein ligase homolog	#N/A	#N/A	#N/A	-12.91	#N/A	#N/A
	ISG15	ISG15 ubiquitin like modifier	#N/A	#N/A	#N/A	#N/A	#N/A	2.64
	POT1	protection of telomeres 1	#N/A	#N/A	#N/A	#N/A	#N/A	2.36
	RAD18	RAD18 E3 ubiquitin protein ligase	#N/A	2.31	#N/A	#N/A	#N/A	#N/A
	RCHY1	ring finger and CHY zinc finger domain containing 1	-2.48	#N/A	#N/A	#N/A	#N/A	#N/A
	SPRTN	SprT-like N-terminal domain	#N/A	#N/A	#N/A	#N/A	#N/A	-2.73

	TERF1	telomeric repeat binding factor 1	-4.09	#N/A	#N/A	#N/A	#N/A	#N/A
	TERF2IP	TERF2 interacting protein	#N/A	2.36	#N/A	#N/A	#N/A	#N/A
	UBE2L6	ubiquitin conjugating enzyme E2 L6	#N/A	#N/A	#N/A	#N/A	#N/A	3.66
	USP10	ubiquitin specific peptidase 10	#N/A	#N/A	#N/A	#N/A	#N/A	-2.39
	VCP	valosin containing protein	-2.15	#N/A	#N/A	#N/A	#N/A	-2.24
**Reactome DNA Repair and WP DNA Repair Pathways Full Network**	*APEX1*	apurinic/apyrimidinic endodeoxyribonuclease 1	#N/A	-2.75	#N/A	#N/A	#N/A	#N/A
	FANCA	FA complementation group A	-4.69	#N/A	#N/A	-12.69	#N/A	#N/A
	FANCC	FA complementation group C	#N/A	-2.07	#N/A	#N/A	#N/A	#N/A
	FANCD2	FA complementation group D2	-2.72	#N/A	#N/A	#N/A	#N/A	#N/A
	FANCF	FA complementation group F	#N/A	-4.02	#N/A	-2.39	#N/A	#N/A
	FANCG	FA complementation group G	#N/A	#N/A	#N/A	#N/A	2.79	#N/A
	MSH2	mutS homolog 2	-3.08	#N/A	#N/A	#N/A	#N/A	#N/A
	NEIL2	nei like DNA glycosylase 2	#N/A	-2.21	#N/A	#N/A	#N/A	#N/A
	NEIL3	nei like DNA glycosylase 3	#N/A	#N/A	#N/A	#N/A	3.9	#N/A
	SMUG1	single-strand-selective monofunctional uracil-DNA glycosylase 1	-3.73	#N/A	#N/A	#N/A	#N/A	#N/A
	TDG	thymine DNA glycosylase	-2.78	#N/A	-2.62	#N/A	#N/A	#N/A
	USP1	ubiquitin specific peptidase 1	#N/A	#N/A	#N/A	-3.25	#N/A	#N/A
**Reactome DNA Repair and WP DNA Repair Pathways Full Network and Hallmark DNA Repair**	*REV3L*	REV3 like, DNA directed polymerase zeta catalytic subunit	#N/A	#N/A	#N/A	#N/A	-5.27	#N/A

Reactome DNA Repair and Reactome Homology Directed Repair and Reactome DNA Double-Strand Break Repair	*CCNA1*	cyclin A1	#N/A	#N/A	#N/A	#N/A	-3.17	#N/A
	CCNA2	cyclin A2	#N/A	#N/A	#N/A	#N/A	11	-6.22
	CDK2	cyclin dependent kinase 2	-5.48	#N/A	#N/A	#N/A	5.4	#N/A
	CLSPN	claspin	-6.7	-7.53	#N/A	-7.42	#N/A	#N/A
	EME1	essential meiotic structure-specific endonuclease 1	#N/A	-2.17	#N/A	#N/A	#N/A	#N/A
	GEN1	GEN1 Holliday junction 5' flap endonuclease	#N/A	-3.15	#N/A	#N/A	#N/A	#N/A
	PPP4C	protein phosphatase 4 catalytic subunit	#N/A	-3.77	#N/A	#N/A	#N/A	#N/A
	RAD51B	RAD51 paralog B	#N/A	5.06	#N/A	#N/A	#N/A	#N/A
	RBBP8	RB binding protein 8, endonuclease	#N/A	-16.17	#N/A	-5.21	-5.47	#N/A
	RMI1	RecQ mediated genome instability 1	-2.24	#N/A	#N/A	#N/A	#N/A	#N/A
	RMI2	RecQ mediated genome instability 2	#N/A	#N/A	#N/A	-4	#N/A	#N/A
	SLX1A; SLX1B	SLX1 homolog A, structure-specific endonuclease subunit; SLX1 homolog B, structure-specific endonuclease subunit	-2.9	#N/A	#N/A	#N/A	#N/A	#N/A
	SLX4	SLX4 structure-specific endonuclease subunit	#N/A	2.01	#N/A	#N/A	2.61	#N/A
	SPIDR	scaffold protein involved in DNA repair	-2.94	#N/A	3.14	#N/A	#N/A	#N/A
	TIMELESS	timeless circadian regulator	-5.44	-2.94	#N/A	-4.48	#N/A	#N/A
	TIPIN	TIMELESS interacting protein	-2.3	-9.65	-4.28	-5.69	#N/A	#N/A
	XRCC2	X-ray repair cross complementing 2	#N/A	#N/A	#N/A	-8.75	#N/A	#N/A
	XRCC3	X-ray repair cross complementing 3	#N/A	-3.57	#N/A	-2.69	#N/A	-3.43
Reactome DNA Repair and Reactome DNA Double-Strand	*APBB1*	amyloid beta precursor protein binding family B member 1	#N/A	#N/A	#N/A	#N/A	#N/A	2.26
	EYA1	EYA transcriptional coactivator and phosphatase 1	#N/A	#N/A	#N/A	#N/A	5.58	#N/A

**Break Repair**	*EYA4*	EYA transcriptional coactivator and phosphatase 4	-2.08	#N/A	#N/A	#N/A	-4.64	#N/A
	KDM4A	lysine demethylase 4A	#N/A	#N/A	#N/A	-4.24	#N/A	#N/A
	KDM4B	lysine demethylase 4B	2.67	#N/A	2.96	#N/A	#N/A	3.41
	KPNA2	karyopherin subunit alpha 2	#N/A	#N/A	#N/A	#N/A	#N/A	-19.36
	PPP5C	protein phosphatase 5 catalytic subunit	-3.18	-2.63	#N/A	-2.59	#N/A	#N/A
	SMARCA5	SWI/SNF related, matrix associated, actin dependent regulator of chromatin, subfamily a, member 5	#N/A	-4.1	#N/A	#N/A	#N/A	#N/A
	TDP1	tyrosyl-DNA phosphodiesterase 1	#N/A	#N/A	#N/A	#N/A	2.03	#N/A
	TDP2	tyrosyl-DNA phosphodiesterase 2	#N/A	#N/A	#N/A	#N/A	-2.25	#N/A
**Reactome DNA Repair and WP DNA Repair Pathways Full Network and Reactome Homology Directed Repair and Reactome DNA Double-Strand Break Repair**	*ATM*	ATM serine/threonine kinase	#N/A	2.37	#N/A	#N/A	#N/A	#N/A
	ATR	ATR serine/threonine kinase	#N/A	-2.55	#N/A	#N/A	-3.1	#N/A
	BRCA1	BRCA1 DNA repair associated	#N/A	#N/A	#N/A	-8.73	#N/A	#N/A
	EXO1	exonuclease 1	#N/A	#N/A	#N/A	-12.97	#N/A	#N/A
	NBN	nibrin	-3.16	-2.67	#N/A	#N/A	#N/A	#N/A
	RAD51C	RAD51 paralog C	#N/A	#N/A	#N/A	-3.55	#N/A	#N/A
**Reactome DNA Repair and Hallmark DNA Repair and Reactome DNA Double-Strand**	*TP53*	tumor protein p53	#N/A	#N/A	#N/A	-4.37	#N/A	#N/A

**Break Repair**								
**Reactome DNA Repair and WP DNA Repair Pathways Full Network and Reactome DNA Double-Strand Break Repair**	*LIG4*	DNA ligase 4	#N/A	#N/A	#N/A	#N/A	-5.34	#N/A
	PRKDC	protein kinase, DNA-activated, catalytic subunit	-3.82	#N/A	#N/A	#N/A	#N/A	#N/A
	XRCC4	X-ray repair cross complementing 4	#N/A	#N/A	-2.73	#N/A	#N/A	#N/A
	XRCC5	X-ray repair cross complementing 5	-2.71	-2.32	#N/A	#N/A	#N/A	#N/A
	XRCC6	X-ray repair cross complementing 6	#N/A	-2.18	#N/A	#N/A	#N/A	#N/A
**Reactome DNA Repair and Reactome Homology Directed Repair and Reactome DNA Double-Strand Break Repair and Reactome Nucleotide Excision Repair**	*UBE2N*	ubiquitin conjugating enzyme E2 N	#N/A	-3.21	-3.99	#N/A	#N/A	-2.12
**Reactome DNA Repair and Reactome Nucleotide Excision Repair**	*ACTB*	actin beta	-2.36	#N/A	#N/A	#N/A	#N/A	#N/A
	ACTL6A	actin like 6A	#N/A	-2.27	#N/A	#N/A	#N/A	-2.54
	ACTR5	actin related protein 5	#N/A	#N/A	-2.39	#N/A	#N/A	#N/A
	AQR	aquarius intron-binding spliceosomal factor	#N/A	#N/A	#N/A	#N/A	-2.2	#N/A
	CHD1L	chromodomain helicase DNA binding protein 1 like	-2.55	#N/A	#N/A	#N/A	#N/A	#N/A
	COPS2	COP9 signalosome subunit 2	#N/A	2.69	#N/A	#N/A	#N/A	#N/A
	COPS3	COP9 signalosome subunit 3	#N/A	-2.49	#N/A	-3.15	#N/A	#N/A
	COPS8	COP9 signalosome subunit 8	#N/A	#N/A	-2.61	#N/A	#N/A	#N/A
	GPS1	G protein pathway suppressor 1	#N/A	-8.51	#N/A	-2.49	#N/A	#N/A
	INO80B	INO80 complex subunit B	#N/A	#N/A	-2.14	#N/A	#N/A	#N/A

	MCRS1	microspherule protein 1	-2.11	-4.35	#N/A	-2.38	#N/A	#N/A
	PIAS1	protein inhibitor of activated STAT 1	#N/A	3.22	3.02	#N/A	#N/A	#N/A
	PIAS3	protein inhibitor of activated STAT 3	-2.28	#N/A	#N/A	#N/A	#N/A	#N/A
	POLR2B	RNA polymerase II subunit B	-2.03	#N/A	#N/A	#N/A	#N/A	-2.58
	POLR2L	RNA polymerase II, I and III subunit L	-4.08	-8.5	#N/A	#N/A	#N/A	#N/A
	PRPF19	pre-mRNA processing factor 19	-2.91	-2.49	#N/A	#N/A	#N/A	#N/A
	RUVBL1	RuvB like AAA ATPase 1	#N/A	-9.55	#N/A	#N/A	#N/A	#N/A
	ZNF830	zinc finger protein 830	#N/A	#N/A	-4.44	#N/A	#N/A	#N/A
**Reactome DNA Repair and Hallmark DNA Repair and WP DNA Repair Pathways Full Network and Reactome Nucleotide Excision Repair**	*DDB2*	damage specific DNA binding protein 2	#N/A	17.29	4.05	#N/A	#N/A	3.03
	ERCC5	ERCC excision repair 5, endonuclease	#N/A	3.51	2.46	2.16	#N/A	#N/A
	ERCC6	ERCC excision repair 6, chromatin remodeling factor	#N/A	#N/A	#N/A	#N/A	#N/A	2.64
	GTF2H3	general transcription factor IIH subunit 3	#N/A	-3.69	#N/A	#N/A	#N/A	#N/A
	LIG1	DNA ligase 1	#N/A	#N/A	#N/A	#N/A	3.38	#N/A
**Reactome DNA Repair and WP DNA Repair Pathways Full Network and Reactome Homology Directed Repair and Reactome DNA Double-Strand Break Repair and Reactome Nucleotide Excision Repair**	*PARP1*	poly(ADP-ribose) polymerase 1	-2.79	-2.11	#N/A	-3.63	#N/A	#N/A
	POLD2	DNA polymerase delta 2, accessory subunit	#N/A	-5.02	#N/A	-5.77	#N/A	#N/A
	POLE	DNA polymerase epsilon, catalytic subunit	#N/A	#N/A	#N/A	-2.73	#N/A	#N/A
	POLE2	DNA polymerase epsilon 2, accessory subunit	#N/A	#N/A	#N/A	-6.3	#N/A	#N/A
	POLE3	DNA polymerase epsilon 3, accessory subunit	-2.15	#N/A	#N/A	#N/A	#N/A	#N/A
	RFC1	replication factor C subunit 1	#N/A	3.75	#N/A	#N/A	#N/A	#N/A
	RPA1	replication protein A1	-2.25	#N/A	#N/A	#N/A	#N/A	#N/A

**Reactome DNA Repair and WP DNA Repair Pathways Full Network and Reactome Nucleotide Excision Repair**	*CDK7*	cyclin dependent kinase 7	#N/A	2.83	#N/A	#N/A	#N/A	#N/A
	CUL4B	cullin 4B	#N/A	4.09	#N/A	#N/A	#N/A	4.89
	ERCC8	ERCC excision repair 8, CSA ubiquitin ligase complex subunit	#N/A	-2.75	#N/A	#N/A	#N/A	#N/A
	GTF2H2	general transcription factor IIH subunit 2	#N/A	-3.85	#N/A	#N/A	#N/A	#N/A
	RAD23A	RAD23 homolog A, nucleotide excision repair protein	#N/A	#N/A	#N/A	-2.18	#N/A	-2.28
**Reactome DNA Repair and Hallmark DNA repair and Reactome Nucleotide Excision Repair**	*POLR2A*	RNA polymerase II subunit A	#N/A	#N/A	-2.25	#N/A	#N/A	#N/A
	POLR2C	RNA polymerase II subunit C	#N/A	2.11	#N/A	#N/A	#N/A	#N/A
	POLR2E	RNA polymerase II, I and III subunit E	-2.84	-2.59	#N/A	#N/A	#N/A	#N/A
	POLR2H	RNA polymerase II, I and III subunit H	#N/A	#N/A	#N/A	#N/A	2.18	#N/A
	POLR2K	RNA polymerase II, I and III subunit K	-2.66	-2.38	-2.15	#N/A	#N/A	#N/A
**Reactome DNA Repair and Hallmark DNA repair and WP DNA Repair Pathways Full Network and Reactome Homology Directed Repair and Reactome DNA Double-Strand Break Repair and Reactome Nucleotide Excision Repair**	*ERCC4*	ERCC excision repair 4, endonuclease catalytic subunit	#N/A	#N/A	#N/A	#N/A	-2.93	#N/A
	PCNA	proliferating cell nuclear antigen	-8.2	#N/A	#N/A	-11.04	#N/A	#N/A
	POLE4	DNA polymerase epsilon 4, accessory subunit	-2.25	-2.31	#N/A	-5.02	2.79	#N/A
	RFC2	replication factor C subunit 2	#N/A	#N/A	#N/A	-7.02	#N/A	#N/A
	RFC3	replication factor C subunit 3	-2.13	#N/A	#N/A	-6.33	#N/A	#N/A
	RFC4	replication factor C subunit 4	-2.15	#N/A	#N/A	-2.96	#N/A	#N/A
	RPA2	replication protein A2	-2.2	#N/A	#N/A	#N/A	#N/A	#N/A
**Reactome DNA Repair and**	*FEN1*	flap structure-specific endonuclease 1	-5.14	-8.8	#N/A	-6.24	4.63	#N/A

**Hallmark DNA repair and WP DNA Repair Pathways Full Network and Reactome Homology Directed Repair and Reactome DNA Double-Strand Break Repair**	*POLH*	DNA polymerase eta	-3.21	7.09	4.72	#N/A	9.75	#N/A
	RAD51	RAD51 recombinase	#N/A	#N/A	#N/A	-3.92	#N/A	#N/A

Supplementary Table 7. Fold change of DNA replication-related genes based on pathway analysis across cell lines

Pathways	Gene Symbol	Gene Description	H226	MCF-7	HT-29	Capan-1	SKOV-3	MIA PaCa-2
**Reactome DNA Replication and Reactome Synthesis of DNA**	*FEN1*	flap structure-specific endonuclease 1	-5.14	-8.8	#N/A	-6.24	4.63	#N/A
	GINS1	GINS complex subunit 1	#N/A	-6.72	#N/A	-3.78	9.19	#N/A
	GINS2	GINS complex subunit 2	-5.44	-7.96	#N/A	-12.59	#N/A	#N/A
	GINS3	GINS complex subunit 3	-2.08	#N/A	#N/A	-3.27	#N/A	#N/A
	GINS4	GINS complex subunit 4	#N/A	-2.92	#N/A	-3.32	2.54	#N/A
	LIG1	DNA ligase 1	#N/A	#N/A	#N/A	#N/A	3.38	#N/A
**Reactome DNA Replication and Reactome Synthesis of DNA and Reactome DNA Replication, Preinitiation and Reactome Activation of Prereplicative Complex**	*MCM8*	minichromosome maintenance 8 homologous recombination repair factor	-3.82	#N/A	#N/A	#N/A	#N/A	#N/A
	POLE	DNA polymerase epsilon, catalytic subunit	#N/A	#N/A	#N/A	-2.73	#N/A	#N/A
	POLE3	DNA polymerase epsilon 3, accessory subunit	-2.15	#N/A	#N/A	#N/A	#N/A	#N/A
	POLE4	DNA polymerase epsilon 4, accessory subunit	-2.25	-2.31	#N/A	-5.02	2.79	#N/A
	PRIM1	DNA primase subunit 1	-5.17	#N/A	#N/A	-4.76	#N/A	#N/A
	PRIM2	DNA primase subunit 2	#N/A	#N/A	#N/A	#N/A	2.68	#N/A
**Reactome DNA Replication and Reactome synthesis of DNA and Reactome DNA Replication, Preinitiation and Reactome Switching of Origins to Post Replicative State**	*PSMA3*	proteasome 20S subunit alpha 3	#N/A	#N/A	#N/A	#N/A	#N/A	-2.99
	PSMA5	proteasome 20S subunit alpha 5	-2.35	-2.28	-2.27	#N/A	#N/A	-3.03
	PSMA7	proteasome 20S subunit alpha 7	#N/A	-3.63	#N/A	#N/A	#N/A	#N/A
	PSMB2	proteasome 20S subunit beta 2	-3.03	#N/A	#N/A	#N/A	#N/A	-2.23
	PSMB3	proteasome 20S subunit beta 3	-2.35	-2.34	#N/A	#N/A	#N/A	#N/A
	PSMB5	proteasome 20S subunit beta 5	-3.12	#N/A	#N/A	#N/A	#N/A	#N/A
	PSMB7	proteasome 20S subunit beta 7	-2.25	-3.2	#N/A	#N/A	#N/A	#N/A
	PSMB8	proteasome 20S subunit beta 8	-3.56	#N/A	#N/A	#N/A	#N/A	3.1
	PSMB9	proteasome 20S subunit beta 9	#N/A	#N/A	#N/A	#N/A	#N/A	4.81
	PSMC3	proteasome 26S subunit, ATPase 3	#N/A	-3.58	#N/A	-2.27	#N/A	#N/A

	PSMC4	proteasome 26S subunit, ATPase 4	-4.42	-4.15	#N/A	#N/A	#N/A	#N/A
	PSMC5	proteasome 26S subunit, ATPase 5	#N/A	-2.99	#N/A	#N/A	#N/A	#N/A
	PSMD1	proteasome 26S subunit, non-ATPase 1	-2.19	-3.17	#N/A	#N/A	#N/A	#N/A
	PSMD11	proteasome 26S subunit, non-ATPase 11	#N/A	-2.51	#N/A	#N/A	#N/A	#N/A
	PSMD12	proteasome 26S subunit, non-ATPase 12	-2.51	#N/A	-3.31	#N/A	#N/A	-4.51
	PSMD14	proteasome 26S subunit, non-ATPase 14	#N/A	-3.99	#N/A	#N/A	#N/A	#N/A
	PSMD2	proteasome 26S subunit ubiquitin receptor, non-ATPase 2	#N/A	-2.79	#N/A	#N/A	#N/A	#N/A
	PSMD3	proteasome 26S subunit, non-ATPase 3	-2.15	-3.45	#N/A	#N/A	#N/A	#N/A
	PSMD5	proteasome 26S subunit, non-ATPase 5	#N/A	#N/A	-2.66	#N/A	#N/A	-2.31
	PSMD8	proteasome 26S subunit, non-ATPase 8	-2.51	#N/A	#N/A	#N/A	#N/A	#N/A
	PSME2	proteasome activator subunit 2	#N/A	-3.4	#N/A	#N/A	#N/A	#N/A
	PSME3	proteasome activator subunit 3	-2.85	-13.18	-5.73	-2.97	#N/A	-5.57
**Reactome DNA Replication and Reactome Synthesis of DNA and Reactome Switching of Origins to Post Replicative State**	*ANAPC15*	anaphase promoting complex subunit 15	-4.57	-4.9	-4.11	#N/A	#N/A	#N/A
	ANAPC5	anaphase promoting complex subunit 5	-3.55	-3.06	#N/A	#N/A	#N/A	#N/A
	ANAPC7	anaphase promoting complex subunit 7	-2.99	-6.91	-3.63	#N/A	#N/A	-3.58
	CCNA1	cyclin A1	#N/A	#N/A	#N/A	#N/A	-3.17	#N/A
	CCNA2	cyclin A2	#N/A	#N/A	#N/A	#N/A	11	-6.22
	CCNE2	cyclin E2	-6.15	-16.51	#N/A	#N/A	7.12	#N/A
	CDC16	cell division cycle 16	#N/A	#N/A	#N/A	#N/A	#N/A	-2.12
	CDC26	cell division cycle 26	-2.02	#N/A	#N/A	#N/A	#N/A	#N/A
	CUL1	cullin 1	#N/A	-2.02	#N/A	#N/A	#N/A	#N/A
	SKP1	S-phase kinase associated protein 1	#N/A	-2.63	#N/A	#N/A	#N/A	#N/A
	SKP2	S-phase kinase associated protein 2	-8.8	-7.94	#N/A	#N/A	#N/A	#N/A
	UBE2C	ubiquitin conjugating enzyme E2 C	#N/A	#N/A	#N/A	#N/A	27.98	#N/A
	UBE2D1	ubiquitin conjugating enzyme E2 D1	#N/A	#N/A	#N/A	2.82	#N/A	#N/A

	UBE2S	ubiquitin conjugating enzyme E2 S	#N/A	#N/A	-4.03	#N/A	#N/A	-6.48
**Reactome DNA Replication and Reactome DNA Replication Preinitiation**	*E2F1*	E2F transcription factor 1	#N/A	-5.27	#N/A	-3.43	#N/A	#N/A
	E2F3	E2F transcription factor 3	-3.27	#N/A	-2.7	#N/A	#N/A	#N/A
**Reactome DNA Replication and Reactome DNA Replication Preinitiation and Reactome Activation of Prereplicative Complex**	*DBF4*	DBF4 zinc finger	#N/A	#N/A	#N/A	#N/A	#N/A	-2.62
	MCM10	minichromosome maintenance 10 replication initiation factor	#N/A	-2.29	#N/A	-2.5	#N/A	#N/A
**Reactome DNA Replication and WP DNA Replication and Reactome Synthesis of DNA**	*PCNA*	proliferating cell nuclear antigen	-8.2	#N/A	#N/A	-11.04	#N/A	#N/A
	POLD2	DNA polymerase delta 2, accessory subunit	#N/A	-5.02	#N/A	-5.77	#N/A	#N/A
	RFC1	replication factor C subunit 1	#N/A	3.75	#N/A	#N/A	#N/A	#N/A
	RFC2	replication factor C subunit 2	#N/A	#N/A	#N/A	-7.02	#N/A	#N/A
	RFC3	replication factor C subunit 3	-2.13	#N/A	#N/A	-6.33	#N/A	#N/A
	RFC4	replication factor C subunit 4	-2.15	#N/A	#N/A	-2.96	#N/A	#N/A
**Reactome DNA Replication and WP DNA Replication and Reactome Synthesis of DNA and Reactome DNA Replication Preinitiation and Reactome Activation of Prereplicative Complex**	*CDC45*	cell division cycle 45	#N/A	-7.73	#N/A	-9.42	#N/A	#N/A
	POLE2	DNA polymerase epsilon 2, accessory subunit	#N/A	#N/A	#N/A	-6.3	#N/A	#N/A
	RPA1	replication protein A1	-2.25	#N/A	#N/A	#N/A	#N/A	#N/A
	RPA2	replication protein A2	-2.2	#N/A	#N/A	#N/A	#N/A	#N/A
	CDC6	cell division cycle 6	-11.03	-3.72	#N/A	-12.35	#N/A	#N/A
	CDK2	cyclin dependent kinase 2	-5.48	#N/A	#N/A	#N/A	5.4	#N/A
	CDT1	chromatin licensing and DNA replication factor 1	-3.42	#N/A	#N/A	-3.83	#N/A	#N/A
	KPNA1	karyopherin subunit alpha 1	#N/A	#N/A	-2.35	#N/A	#N/A	#N/A
	KPNB1	karyopherin subunit beta 1	-2.06	#N/A	#N/A	#N/A	#N/A	#N/A
	MCM2	minichromosome maintenance complex component 2	#N/A	-8.26	#N/A	-11.27	#N/A	#N/A
	MCM3	minichromosome maintenance complex component 3	-9.81	-12.53	#N/A	-12.73	#N/A	#N/A

	MCM4	minichromosome maintenance complex component 4	-5.39	#N/A	#N/A	-11.9	#N/A	#N/A
	MCM5	minichromosome maintenance complex component 5	#N/A	-4.96	#N/A	-18.01	#N/A	#N/A
	MCM6	minichromosome maintenance complex component 6	-8.07	-6.76	#N/A	-8.84	#N/A	#N/A
	MCM7	minichromosome maintenance complex component 7	-5.51	-8.78	#N/A	-4.25	#N/A	#N/A
	ORC1	origin recognition complex subunit 1	-3.95	#N/A	#N/A	#N/A	#N/A	#N/A
	ORC5	origin recognition complex subunit 5	#N/A	-2.95	-3.14	#N/A	#N/A	#N/A
**Reactome DNA Replication and WP DNA Replication and Reactome DNA Replication Preinitiation and Reactome Activation of Prereplicative Complex**	*CDC7*	cell division cycle 7	-5.85	-3.96	#N/A	-5.12	#N/A	#N/A
	GMNN	geminin DNA replication inhibitor	-11.09	#N/A	#N/A	#N/A	#N/A	#N/A

Supplementary Table 8. Fold change of chromosomal stability-related genes based on pathway analysis across cell lines

Pathways	Gene Symbol	Gene Description	H226	MCF-7	HT-29	Capan-1	SKOV-3	MIA PaCa-2
**Reactome Chromosome Maintenance**	*CCNA1*	cyclin A1	#N/A	#N/A	#N/A	#N/A	-3.17	#N/A
	CCNA2	cyclin A2	#N/A	#N/A	#N/A	#N/A	11	-6.22
	CDK2	cyclin dependent kinase 2	-5.48	#N/A	#N/A	#N/A	5.4	#N/A
	CENPW	centromere protein W	#N/A	#N/A	#N/A	#N/A	7.6	#N/A
	CHTF8	chromosome transmission fidelity factor 8	#N/A	-3.35	#N/A	#N/A	#N/A	#N/A
	DKC1	dyskerin pseudouridine synthase 1	-3.02	-3.32	-4.67	-3.03	#N/A	-3.66
	FEN1	flap structure-specific endonuclease 1	-5.14	-8.8	#N/A	-6.24	4.63	#N/A
	GAR1	GAR1 ribonucleoprotein	-2.8	#N/A	-2.84	#N/A	#N/A	#N/A
	HJURP	Holliday junction recognition protein	#N/A	#N/A	#N/A	#N/A	9.32	#N/A
	LIG1	DNA ligase 1	#N/A	#N/A	#N/A	#N/A	3.38	#N/A
	NHP2	NHP2 ribonucleoprotein	#N/A	-2.76	#N/A	#N/A	#N/A	#N/A
	NPM1	nucleophosmin 1	#N/A	#N/A	#N/A	#N/A	#N/A	-2.72
	OIP5	Opa interacting protein 5	#N/A	#N/A	#N/A	#N/A	2.95	#N/A
	PCNA	proliferating cell nuclear antigen	-8.2	#N/A	#N/A	-11.04	#N/A	#N/A
	POLD2	DNA polymerase delta 2, accessory subunit	#N/A	-5.02	#N/A	-5.77	#N/A	#N/A
	POLR2A	RNA polymerase II subunit A	#N/A	#N/A	-2.25	#N/A	#N/A	#N/A
	POLR2B	RNA polymerase II subunit B	-2.03	#N/A	#N/A	#N/A	#N/A	-2.58
	POLR2C	RNA polymerase II subunit C	#N/A	2.11	#N/A	#N/A	#N/A	#N/A
	POLR2E	RNA polymerase II, I and III subunit E	-2.84	-2.59	#N/A	#N/A	#N/A	#N/A
	POLR2H	RNA polymerase II, I and III subunit H	#N/A	#N/A	#N/A	#N/A	2.18	#N/A
	POLR2K	RNA polymerase II, I and III subunit K	-2.66	-2.38	-2.15	#N/A	#N/A	#N/A
	POLR2L	RNA polymerase II, I and III subunit L	-4.08	-8.5	#N/A	#N/A	#N/A	#N/A

	POT1	protection of telomeres 1	#N/A	#N/A	#N/A	#N/A	#N/A	2.36
	PPP6C	protein phosphatase 6 catalytic subunit	#N/A	#N/A	#N/A	#N/A	#N/A	-2.56
	PPP6R3	protein phosphatase 6 regulatory subunit 3	#N/A	4.12	#N/A	#N/A	#N/A	#N/A
	PRIM1	DNA primase subunit 1	-5.17	#N/A	#N/A	-4.76	#N/A	#N/A
	PRIM2	DNA primase subunit 2	#N/A	#N/A	#N/A	#N/A	2.68	#N/A
	RBBP4	RB binding protein 4, chromatin remodeling factor	-3.57	-2.5	#N/A	-5.44	#N/A	#N/A
	RBBP7	RB binding protein 7, chromatin remodeling factor	-2.99	-3.78	#N/A	#N/A	#N/A	#N/A
	RFC1	replication factor C subunit 1	#N/A	3.75	#N/A	#N/A	#N/A	#N/A
	RFC2	replication factor C subunit 2	#N/A	#N/A	#N/A	-7.02	#N/A	#N/A
	RFC3	replication factor C subunit 3	-2.13	#N/A	#N/A	-6.33	#N/A	#N/A
	RFC4	replication factor C subunit 4	-2.15	#N/A	#N/A	-2.96	#N/A	#N/A
	RPA1	replication protein A1	-2.25	#N/A	#N/A	#N/A	#N/A	#N/A
	RPA2	replication protein A2	-2.2	#N/A	#N/A	#N/A	#N/A	#N/A
	RSF1	remodeling and spacing factor 1	#N/A	3.95	#N/A	#N/A	#N/A	#N/A
	RUVBL1	RuvB like AAA ATPase 1	#N/A	-9.55	#N/A	#N/A	#N/A	#N/A
	RUVBL2	RuvB like AAA ATPase 2	-2.43	-2.83	#N/A	#N/A	#N/A	#N/A
	SMARCA5	SWI/SNF related, matrix associated, actin dependent regulator of chromatin, subfamily a, member 5	#N/A	-4.1	#N/A	#N/A	#N/A	#N/A
	TERF1	telomeric repeat binding factor 1	-4.09	#N/A	#N/A	#N/A	#N/A	#N/A
	TERF2IP	TERF2 interacting protein	#N/A	2.36	#N/A	#N/A	#N/A	#N/A
**Resolution of Sister Chromatid Cohesion**	*CCNB1*	cyclin B1	#N/A	#N/A	#N/A	#N/A	#N/A	-9.8
	CCNB2	cyclin B2	#N/A	#N/A	#N/A	#N/A	#N/A	-5.7
	CDK1	cyclin dependent kinase 1	#N/A	#N/A	#N/A	#N/A	12.92	#N/A

	ANAPC15	anaphase promoting complex subunit 15	-4.57	-4.9	-4.11	#N/A	#N/A	#N/A
	ANAPC5	anaphase promoting complex subunit 5	-3.55	-3.06	#N/A	#N/A	#N/A	#N/A
	ANAPC7	anaphase promoting complex subunit 7	-2.99	-6.91	-3.63	#N/A	#N/A	-3.58
	CDC16	cell division cycle 16	#N/A	#N/A	#N/A	#N/A	#N/A	-2.12
	CDC26	cell division cycle 26	-2.02	#N/A	#N/A	#N/A	#N/A	#N/A
	ESPL1	extra spindle pole bodies like 1, separase	#N/A	#N/A	#N/A	#N/A	3.49	#N/A
	PSMA3	proteasome 20S subunit alpha 3	#N/A	#N/A	#N/A	#N/A	#N/A	-2.99
	PSMA5	proteasome 20S subunit alpha 5	-2.35	-2.28	-2.27	#N/A	#N/A	-3.03
	PSMA7	proteasome 20S subunit alpha 7	#N/A	-3.63	#N/A	#N/A	#N/A	#N/A
	PSMB2	proteasome 20S subunit beta 2	-3.03	#N/A	#N/A	#N/A	#N/A	-2.23
	PSMB3	proteasome 20S subunit beta 3	-2.35	-2.34	#N/A	#N/A	#N/A	#N/A
**Reactome Separation of Sister Chromatids**	*PSMB5*	proteasome 20S subunit beta 5	-3.12	#N/A	#N/A	#N/A	#N/A	#N/A
	PSMB7	proteasome 20S subunit beta 7	-2.25	-3.2	#N/A	#N/A	#N/A	#N/A
	PSMB8	proteasome 20S subunit beta 8	-3.56	#N/A	#N/A	#N/A	#N/A	3.1
	PSMB9	proteasome 20S subunit beta 9	#N/A	#N/A	#N/A	#N/A	#N/A	4.81
	PSMC3	proteasome 26S subunit, ATPase 3	#N/A	-3.58	#N/A	-2.27	#N/A	#N/A
	PSMC4	proteasome 26S subunit, ATPase 4	-4.42	-4.15	#N/A	#N/A	#N/A	#N/A
	PSMC5	proteasome 26S subunit, ATPase 5	#N/A	-2.99	#N/A	#N/A	#N/A	#N/A
	PSMD1	proteasome 26S subunit, non-ATPase 1	-2.19	-3.17	#N/A	#N/A	#N/A	#N/A
	PSMD11	proteasome 26S subunit, non-ATPase 11	#N/A	-2.51	#N/A	#N/A	#N/A	#N/A
	PSMD12	proteasome 26S subunit, non-ATPase 12	-2.51	#N/A	-3.31	#N/A	#N/A	-4.51
	PSMD14	proteasome 26S subunit, non-ATPase 14	#N/A	-3.99	#N/A	#N/A	#N/A	#N/A
	PSMD2	proteasome 26S subunit ubiquitin	#N/A	-2.79	#N/A	#N/A	#N/A	#N/A

		receptor, non-ATPase 2						
	PSMD3	proteasome 26S subunit, non-ATPase 3	-2.15	-3.45	#N/A	#N/A	#N/A	#N/A
	PSMD5	proteasome 26S subunit, non-ATPase 5	#N/A	#N/A	-2.66	#N/A	#N/A	-2.31
	PSMD8	proteasome 26S subunit, non-ATPase 8	-2.51	#N/A	#N/A	#N/A	#N/A	#N/A
	PSME2	proteasome activator subunit 2	#N/A	-3.4	#N/A	#N/A	#N/A	#N/A
	PSME3	proteasome activator subunit 3	-2.85	-13.18	-5.73	-2.97	#N/A	-5.57
	PTTG1	PTTG1 regulator of sister chromatid separation, securin	#N/A	#N/A	#N/A	#N/A	14.16	#N/A
	UBE2C	ubiquitin conjugating enzyme E2 C	#N/A	#N/A	#N/A	#N/A	27.98	#N/A
	UBE2D1	ubiquitin conjugating enzyme E2 D1	#N/A	#N/A	#N/A	2.82	#N/A	#N/A
	UBE2S	ubiquitin conjugating enzyme E2 S	#N/A	#N/A	-4.03	#N/A	#N/A	-6.48
**Reactome Separation of Sister Chromatids and Reactome Resolution of Sister Chromatid Cohesion**	*AURKB*	aurora kinase B	#N/A	#N/A	#N/A	#N/A	12.12	#N/A
	BIRC5	baculoviral IAP repeat containing 5	#N/A	#N/A	#N/A	#N/A	4.56	#N/A
	BUB3	BUB3 mitotic checkpoint protein	#N/A	#N/A	#N/A	#N/A	#N/A	-2.35
	CDC20	cell division cycle 20	#N/A	#N/A	#N/A	#N/A	#N/A	-9.18
	CDCA5	cell division cycle associated 5	#N/A	#N/A	#N/A	#N/A	3.25	#N/A
	CDCA8	cell division cycle associated 8	#N/A	#N/A	#N/A	#N/A	8.07	-6.83
	CENPE	centromere protein E	#N/A	#N/A	#N/A	#N/A	#N/A	-6.34
	NUP98	nucleoporin 98 and 96 precursor	#N/A	#N/A	#N/A	#N/A	#N/A	-2.53
	CKAP5	cytoskeleton associated protein 5	#N/A	4.4	#N/A	#N/A	#N/A	#N/A
	DYNC1H1	dynein cytoplasmic 1 heavy chain 1	#N/A	2.5	#N/A	#N/A	#N/A	#N/A
	DYNC1I2	dynein cytoplasmic 1 intermediate chain 2	#N/A	2.1	#N/A	#N/A	#N/A	#N/A
	DYNLL1	dynein light chain LC8-type 1	#N/A	#N/A	-3.39	#N/A	#N/A	#N/A

	KIF2C	kinesin family member 2C	#N/A	#N/A	#N/A	#N/A	7.47	#N/A
	MAD2L1	mitotic arrest deficient 2 like 1	#N/A	#N/A	#N/A	#N/A	8.43	#N/A
	MAPRE1	microtubule associated protein RP/EB family member 1	#N/A	-3.26	#N/A	#N/A	#N/A	#N/A
	NDC80	NDC80 kinetochore complex component	#N/A	#N/A	#N/A	#N/A	10.69	#N/A
	NDE1	nudE neurodevelopment protein 1	#N/A	2.92	#N/A	#N/A	#N/A	#N/A
	NDEL1	nudE neurodevelopment protein 1 like 1	3.15	3.98	#N/A	3.13	#N/A	#N/A
	NUP160	nucleoporin 160	#N/A	#N/A	-2.1	#N/A	#N/A	#N/A
	NUP43	nucleoporin 43	-3.32	#N/A	#N/A	#N/A	#N/A	#N/A
	PAFAH1B1	platelet activating factor acetylhydrolase 1b regulatory subunit 1	#N/A	2.6	#N/A	#N/A	#N/A	#N/A
	PDS5A	PDS5 cohesin associated factor A	#N/A	#N/A	#N/A	#N/A	-3.41	#N/A
	PLK1	polo like kinase 1	#N/A	#N/A	#N/A	#N/A	#N/A	-11.98
	PMF1	polyamine modulated factor 1	-2.67	#N/A	#N/A	-3.39	#N/A	#N/A
	PPP2CA	protein phosphatase 2 catalytic subunit alpha	-2.09	-2.1	#N/A	#N/A	#N/A	-4.97
	PPP2R1A	protein phosphatase 2 scaffold subunit Aalpha	-3.56	#N/A	#N/A	#N/A	#N/A	#N/A
	PPP2R1B	protein phosphatase 2 scaffold subunit Abeta	-2.97	#N/A	#N/A	#N/A	#N/A	#N/A
	PPP2R5B	protein phosphatase 2 regulatory subunit B'beta	2.83	#N/A	#N/A	#N/A	#N/A	2.28
	PPP2R5D	protein phosphatase 2 regulatory subunit B'delta	#N/A	2.16	#N/A	#N/A	#N/A	#N/A
	PPP2R5E	protein phosphatase 2 regulatory subunit B'epsilon	#N/A	#N/A	#N/A	#N/A	-2.52	#N/A

	RANBP2	RAN binding protein 2	#N/A	#N/A	#N/A	#N/A	-5.82	#N/A
	RANGAP1	Ran GTPase activating protein 1	#N/A	#N/A	#N/A	#N/A	2.55	-2.71
	RCC2	regulator of chromosome condensation 2	-3.78	-4.97	#N/A	-5.13	#N/A	-3.93
	SEC13	SEC13 homolog, nuclear pore and COPII coat complex component	#N/A	3.46	#N/A	#N/A	#N/A	#N/A
	SEH1L	SEH1 like nucleoporin	-2.37	-3.56	-4.43	#N/A	#N/A	#N/A
	SKA1	spindle and kinetochore associated complex subunit 1	#N/A	#N/A	#N/A	#N/A	5.36	#N/A
	SMC1A	structural maintenance of chromosomes 1A	#N/A	-2.52	#N/A	#N/A	#N/A	#N/A
	SPC24	SPC24 component of NDC80 kinetochore complex	#N/A	#N/A	#N/A	#N/A	9.17	#N/A
	SPC25	SPC25 component of NDC80 kinetochore complex	#N/A	#N/A	#N/A	-5.15	#N/A	#N/A
	SPDL1	spindle apparatus coiled-coil protein 1	#N/A	#N/A	#N/A	#N/A	8.74	#N/A
	STAG2	stromal antigen 2	#N/A	3.15	#N/A	#N/A	#N/A	4.77
	TAOK1	TAO kinase 1	#N/A	#N/A	#N/A	#N/A	#N/A	2.65
	TUBA3E	tubulin alpha 3e	#N/A	#N/A	#N/A	#N/A	2.87	#N/A
	TUBA4A	tubulin alpha 4a	3.86	#N/A	#N/A	#N/A	13.58	#N/A
	TUBB2B	tubulin beta 2B class Iib	#N/A	#N/A	#N/A	#N/A	#N/A	7.45
	TUBB3	tubulin beta 3 class III	#N/A	#N/A	#N/A	#N/A	3.38	#N/A
	TUBB4B	tubulin beta 4B class Ivb	#N/A	-3.34	#N/A	#N/A	3.58	-4.01
	TUBB8	tubulin beta 8 class VIII	#N/A	-2.15	#N/A	#N/A	3.91	#N/A
	WAPL	WAPL cohesin release factor	#N/A	#N/A	#N/A	#N/A	-3.3	#N/A
	XPO1	exportin 1	#N/A	#N/A	#N/A	#N/A	#N/A	2.63
	ZWILCH	zwilch kinetochore protein	#N/A	-2.32	#N/A	#N/A	#N/A	#N/A
	ZWINT	ZW10 interacting kinetochore protein	#N/A	#N/A	#N/A	#N/A	6.49	#N/A

**Reactome Separation of Sister Chromatids and Reactome Resolution of Sister Chromatid Cohesion and Reactome Chromosome Maintenance**	*CENPH*	centromere protein H	#N/A	#N/A	#N/A	#N/A	4.79	#N/A
	CENPI	centromere protein I	#N/A	#N/A	#N/A	#N/A	9.38	#N/A
	CENPN	centromere protein N	#N/A	#N/A	#N/A	#N/A	5.37	#N/A
	ITGB3BP	integrin subunit beta 3 binding protein	#N/A	#N/A	#N/A	#N/A	3.14	#N/A

Supplementary Table 9. Fold change of H226-specific DNA repair-related genes based on pathway analysis

Pathways	Gene Symbol	Gene Description	Fold Change
**Common WP DNA IR Damage and Cellular Response via ATR and WP DNA Damage Response**	*CDK2*	cyclin-dependent kinase 2	-5.48
	FANCD2	Fanconi anemia complementation group D2	-2.72
	NBN	nibrin	-3.16
	PML	promyelocytic leukemia	3.39
	PRKDC	protein kinase, DNA-activated, catalytic polypeptide	-3.82
	RPA2	replication protein A2	-2.2
**WP DNA Damage Response**	*BID*	BH3 interacting domain death agonist	-2.22
	CASP3	caspase 3	-3.73
	CCND1	cyclin D1	-4.81
	CCND2	cyclin D2	-3.77
	CCND3	cyclin D3	-3.01
	CCNE2	cyclin E2	-6.15
	CDK4	cyclin-dependent kinase 4	-4.42
	CDK5	cyclin-dependent kinase 5	-2.22
	CYCS	cytochrome c, somatic	-3.45
**WP DNA IR Damage and Cellular Response via ATR**	*CLK2*	CDC like kinase 2	-2.05
	CLSPN	claspin	-6.7
	EEF1E1	eukaryotic translation elongation factor 1 epsilon 1	-2.35
	FANCA	Fanconi anemia complementation group A	-4.69
	FEN1	flap structure-specific endonuclease 1	-5.14
	MSH2	mutS homolog 2	-3.08
	PARP1	poly(ADP-ribose) polymerase 1	-2.79
	PCNA	proliferating cell nuclear antigen	-8.2
	PPM1D	protein phosphatase, Mg2+/Mn2+ dependent, 1D	-2.9
	RFWD3	ring finger and WD repeat domain 3	-2.93
	RMI1	RecQ mediated genome instability 1	-2.24

	RPA1	replication protein A1	-2.25
	TRIM28	tripartite motif containing 28	-2.76
	XRCC5	X-ray repair complementing defective repair in Chinese hamster cells 5 (double-strand-break rejoining)	-2.71

Supplementary Table 10. Fold change of MCF-7-specific DNA repair-related genes based on pathway analysis

Pathways	Gene Symbol	Gene Description	Fold Change
**Reactome G1 S DNA Damage Checkpoints and Reactome TP53 Regulates Transcription of DNA Repair Genes**	*ATM*	ATM serine/threonine kinase	2.37
**Reactome G1 S DNA Damage Checkpoints**	*CCNE2*	cyclin E2	-16.51
	CDC25A	cell division cycle 25A	-10.01
	CDKN1A	cyclin-dependent kinase inhibitor 1A (p21, Cip1)	8.91
	MDM2	MDM2 proto-oncogene, E3 ubiquitin protein ligase	5.26
	PSMA5	proteasome subunit alpha 5	-2.28
	PSMA7	proteasome subunit alpha 7	-3.63
	PSMB3	proteasome subunit beta 3	-2.34
	PSMB7	proteasome subunit beta 7	-3.2
	PSMC3	proteasome 26S subunit, ATPase 3	-3.58
	PSMC4	proteasome 26S subunit, ATPase 4 [Source:HGNC Symbol;Acc:HGNC:9551]	-4.15
	PSMC4	proteasome 26S subunit, ATPase 4	-4.03
	PSMC5	proteasome 26S subunit, ATPase 5	-2.99
	PSMD1	proteasome 26S subunit, non-ATPase 1	-3.17
	PSMD11	proteasome 26S subunit, non-ATPase 11	-2.51
	PSMD14	proteasome 26S subunit, non-ATPase 14	-3.99
	PSMD2	proteasome 26S subunit, non-ATPase 2	-2.79
	PSMD3	proteasome 26S subunit, non-ATPase 3	-3.45
	PSME2	proteasome activator subunit 2; microRNA 7703	-3.4
	PSME3	proteasome activator subunit 3	-13.18
**Reactome TP53 Regulates Transcription of DNA Repair Genes**	*ATF2*	activating transcription factor 2	2.9
	ATR	ATR serine/threonine kinase	-2.55
	CCNK	cyclin K	2.5
	CDK9	cyclin-dependent kinase 9	-2.45
	DDB2	damage-specific DNA binding protein 2	17.29
	FANCC	Fanconi anemia complementation group C	-2.07
	JUN	jun proto-oncogene	3.84
	NELFCD	negative elongation factor complex member C/D	-2.2
	SUPT5H	SPT5 homolog, DSIF elongation factor subunit	2.56

**Reactome Transcription Coupled Nucleotide Excision Repair TC-NER**	*COPS2*	COP9 signalosome subunit 2	2.69
	COPS3	COP9 signalosome subunit 3	-2.49
	CUL4B	cullin 4B	4.09
	ERCC5	excision repair cross-complementation group 5	3.51
	ERCC8	excision repair cross-complementation group 8	-2.75
	GPS1	G protein pathway suppressor 1	-8.51
	POLD2	polymerase (DNA directed), delta 2, accessory subunit	-5.02
	POLE4	polymerase (DNA-directed), epsilon 4, accessory subunit	-2.31
	PRPF19	pre-mRNA processing factor 19	-2.49
	RFC1	replication factor C subunit 1	3.75
**Reactome Transcription Coupled Nucleotide Excision Repair TC-NER and Reactome TP53 Regulates Transcription of DNA Repair Genes**	*CDK7*	cyclin-dependent kinase 7	2.83
	GTF2H2	general transcription factor IIH subunit 2	-3.85
	GTF2H3	general transcription factor IIH subunit 3	-3.69
	POLR2C	polymerase (RNA) II (DNA directed) polypeptide C, 33kDa	2.11
	POLR2E	polymerase (RNA) II (DNA directed) polypeptide E, 25kDa	-2.59
	POLR2K	polymerase (RNA) II (DNA directed) polypeptide K, 7.0kDa	-2.38
	POLR2L	polymerase (RNA) II (DNA directed) polypeptide L, 7.6kDa	-8.5

Supplementary Table 11. Fold change of Capan-1-specific DNA replication- and repair-related genes based on pathway analysis

Pathways	Gene Symbol	Gene Description	Fold Change
**Reactome Activation of ATR in Response to Replication Stress**	*CDC25A*	cell division cycle 25A	-8.34
	CDC6	cell division cycle 6	-12.35
	CDC7	cell division cycle 7	-5.12
	CLSPN	claspin	-7.42
	MCM10	minichromosome maintenance 10 replication initiation factor	-2.5
**Reactome HDR through Homologous Recombination (HRR)**	*BRCA1*	breast cancer 1, early onset	-8.73
	EXO1	exonuclease 1	-12.97
	RAD51	RAD51 recombinase	-3.92
	RAD51C	RAD51 paralog C	-3.55
	RBBP8	retinoblastoma binding protein 8; microRNA 4741	-5.21
	RMI2	RecQ mediated genome instability 2	-4
	XRCC2	X-ray repair complementing defective repair in Chinese hamster cells 2	-8.75
	XRCC3	X-ray repair complementing defective repair in Chinese hamster cells 3	-2.69
**Reactome HDR through Homologous Recombination (HRR) and Kegg DNA Replication**	*POLE*	polymerase (DNA directed), epsilon, catalytic subunit	-2.73
	POLE2	polymerase (DNA directed), epsilon 2, accessory subunit	-6.3
	POLE4	polymerase (DNA-directed), epsilon 4, accessory subunit	-5.02
**Kegg DNA Replication and Reactome DNA Strand Elongation and Reactome Activation of ATR in Response to Replication Stress**	*MCM2*	minichromosome maintenance complex component 2	-11.27
	MCM3	minichromosome maintenance complex component 3	-12.73
	MCM4	minichromosome maintenance complex component 4	-11.9
	MCM5	minichromosome maintenance complex component 5	-18.01

	MCM6	minichromosome maintenance complex component 6	-8.84
	MCM7	minichromosome maintenance complex component 7	-4.25
**Kegg DNA Replication and Reactome DNA Strand Elongation and Reactome HDR through Homologous Recombination (HRR)**	*PCNA*	proliferating cell nuclear antigen	-11.04
	POLD2	polymerase (DNA directed), delta 2, accessory subunit	-5.77
**Kegg DNA Replication and Reactome DNA Strand Elongation and Reactome HDR through Homologous Recombination (HRR) and Reactome Activation of ATR in Response to Replication Stress**	*RFC2*	replication factor C subunit 2	-7.02
	RFC3	replication factor C subunit 3	-6.33
	RFC4	replication factor C subunit 4	-2.96
**Reactome DNA Strand Elongation**	*GINS1*	GINS complex subunit 1 (Psf1 homolog)	-3.78
	GINS2	GINS complex subunit 2 (Psf2 homolog)	-12.59
	GINS3	GINS complex subunit 3 (Psf3 homolog)	-3.27
	GINS4	GINS complex subunit 4 (Sld5 homolog)	-3.32
**Reactome DNA Strand Elongation and Reactome Activation of ATR in Response to Replication Stress**	*CDC45*	cell division cycle 45	-9.42
**Reactome DNA Strand Elongation and Kegg DNA Replication**	*FEN1*	flap structure-specific endonuclease 1	-6.24
	PRIM1	primase, DNA, polypeptide 1 (49kDa)	-4.76

Supplementary Table 12. Fold change of immune-related genes based on pathway analysis across cell lines

Pathways	Gene Symbol	Gene Description	H226	MCF -7	HT -29	Capa n-1	SKO V-3	MIA PaCa -2
**Class I MHC Mediated Antigen Processing & Presentation**	*HLA-E*	major histocompatibility complex, class I, E	N/A	N/A	N/A	N/A	N/A	3.17
	HLA-F	major histocompatibility complex, class I, F	N/A	N/A	N/A	N/A	N/A	3.61
	HSPA5	heat shock protein family A (Hsp70) member 5	N/A	2.18	N/A	N/A	N/A	N/A
	SAR1B	secretion associated Ras related GTPase 1B	N/A	N/A	-2.03	N/A	N/A	N/A
	SEC13	SEC13 homolog, nuclear pore and COPII coat complex component	N/A	3.46	N/A	N/A	N/A	N/A
	SEC22B	SEC22 homolog B, vesicle trafficking protein	N/A	2.14	N/A	N/A	N/A	N/A
	SEC23A	SEC23 homolog A, COPII coat complex component	N/A	6.82	N/A	N/A	N/A	N/A
	SEC24A	SEC24 homolog A, COPII coat complex component	N/A	7.27	N/A	N/A	N/A	N/A
	SEC24D	SEC24 homolog D, COPII coat complex component	N/A	2.44	N/A	N/A	N/A	6.86
	SEC31A	SEC31 homolog A, COPII coat complex component	N/A	5.28	N/A	N/A	N/A	N/A
	SEC61G	SEC61 translocon subunit gamma	N/A	2.76	N/A	N/A	N/A	4.83
	VAMP3	vesicle associated membrane protein 3	N/A	2.12	N/A	N/A	N/A	N/A
**Class I MHC Mediated Antigen Processing & Presentation and Antigen Processing Ubiquitination Proteosome Degradation**	*ANAPC13*	anaphase promoting complex subunit 13	-2.49	-2.45	N/A	N/A	N/A	-2.07
	ANAPC5	anaphase promoting complex subunit 5	-3.55	-3.06	N/A	N/A	N/A	N/A
	ANAPC7	anaphase promoting complex subunit 7	-2.99	-6.91	-3.63	N/A	N/A	-3.58
	ASB3	ankyrin repeat and SOCS box containing 3	N/A	4.32	N/A	N/A	N/A	N/A
	ASB9	ankyrin repeat and SOCS box containing 9	N/A	N/A	N/A	N/A	N/A	4.21
	CDC16	cell division cycle 16	N/A	N/A	N/A	N/A	N/A	-2.12
	CDC20	cell division cycle 20	N/A	N/A	N/A	N/A	N/A	-9.18
	CDC26	cell division cycle 26	-2.02	N/A	N/A	N/A	N/A	N/A
	CUL3	cullin 3	N/A	N/A	N/A	N/A	N/A	-6.47
	CUL7	cullin 7	N/A	4.04	N/A	N/A	N/A	5.29

FBXL3	F-box and leucine rich repeat protein 3	N/A	N/A	-3.29	N/A	N/A	N/A
FBXO15	F-box protein 15	N/A	-2.1	N/A	N/A	N/A	N/A
FBXO32	F-box protein 32	N/A	3.69	4.39	N/A	N/A	N/A
FBXO9	F-box protein 9	-3.27	N/A	N/A	N/A	N/A	N/A
GAN	gigaxonin	N/A	2.47	N/A	N/A	N/A	N/A
HACE1	HECT domain and ankyrin repeat containing E3 ubiquitin protein ligase 1	N/A	2.09	N/A	N/A	N/A	2.15
HERC3	HECT and RLD domain containing E3 ubiquitin protein ligase 3	N/A	2.68	N/A	N/A	N/A	N/A
KCTD7	potassium channel tetramerization domain containing 7	N/A	2.22	N/A	N/A	N/A	3.44
MKRN1	makorin ring finger protein 1	N/A	2.45	3.09	N/A	N/A	N/A
NEDD4L	NEDD4 like E3 ubiquitin protein ligase	N/A	N/A	N/A	N/A	N/A	36.55
NPEPPS	aminopeptidase puromycin sensitive	N/A	2.07	3.21	N/A	N/A	3.48
PSMA3	proteasome 20S subunit alpha 3	N/A	N/A	N/A	N/A	N/A	-2.99
PSMB9	proteasome 20S subunit beta 9	N/A	N/A	N/A	N/A	N/A	4.81
PSMD5	proteasome 26S subunit, non-ATPase 5	N/A	N/A	-2.66	N/A	N/A	-2.31
RBBP6	RB binding protein 6, ubiquitin ligase	N/A	3.31	N/A	N/A	N/A	N/A
RLIM	ring finger protein, LIM domain interacting	N/A	2.46	N/A	N/A	N/A	N/A
RNF144B	ring finger protein 144B	2.99	N/A	N/A	N/A	N/A	N/A
RNF25	ring finger protein 25	N/A	N/A	-3.16	N/A	N/A	N/A
SKP2	S-phase kinase associated protein 2	-8.8	-7.94	N/A	N/A	N/A	N/A
SOCS3	suppressor of cytokine signaling 3	-2.08	N/A	N/A	N/A	N/A	N/A
TRIM9	tripartite motif containing 9	N/A	2.44	N/A	N/A	N/A	N/A
UBA1	ubiquitin like modifier activating enzyme 1	-3.42	N/A	N/A	N/A	N/A	N/A
UBE2D4	ubiquitin conjugating enzyme E2 D4 (putative)	N/A	-7.53	N/A	N/A	N/A	N/A
UBE2F	ubiquitin conjugating enzyme E2 F (putative)	3.06	N/A	-2.6	N/A	N/A	N/A

	UBE2G2	ubiquitin conjugating enzyme E2 G2	-3.58	-4.05	N/A	N/A	N/A	-2.45
	UBE2L6	ubiquitin conjugating enzyme E2 L6	N/A	N/A	N/A	N/A	N/A	3.66
	UBE2Q2	ubiquitin conjugating enzyme E2 Q2	3.37	N/A	N/A	N/A	N/A	N/A
	UBE2S	ubiquitin conjugating enzyme E2 S	N/A	N/A	-4.03	N/A	N/A	-6.48
	UBR1	ubiquitin protein ligase E3 component n-recognin 1	N/A	N/A	N/A	N/A	N/A	2.01
	WWP1	WW domain containing E3 ubiquitin protein ligase 1	N/A	2.99	N/A	N/A	N/A	N/A
**Class I MHC Mediated Antigen Processing & Presentation and Innate Immune Response**	*B2M*	beta-2-microglobulin	N/A	2.85	N/A	N/A	N/A	4.08
	CTSV	cathepsin V	N/A	5.51	N/A	N/A	N/A	N/A
	LY96	lymphocyte antigen 96	-2.04	N/A	N/A	N/A	N/A	N/A
	S100A8	S100 calcium binding protein A8	N/A	-3.93	N/A	N/A	N/A	N/A
**Class I MHC Mediated Antigen Processing & Presentation and Adaptive Immune Response**	*BCAP31*	B cell receptor associated protein 31	N/A	-2.28	N/A	N/A	N/A	N/A
	CALR	calreticulin	N/A	2.17	N/A	N/A	N/A	N/A
	SEC24B	SEC24 homolog B, COPII coat complex component	N/A	2.04	N/A	N/A	N/A	N/A
	SEC61B	SEC61 translocon subunit beta	N/A	-5.33	N/A	N/A	N/A	N/A
	STX4	syntaxin 4	N/A	2.17	N/A	N/A	N/A	2.09
	TAP1	transporter 1, ATP binding cassette subfamily B member	N/A	2.96	N/A	N/A	N/A	N/A
	TAP2	transporter 2, ATP binding cassette subfamily B member	-2.57	N/A	N/A	N/A	N/A	N/A
**Class I MHC Mediated Antigen Processing & Presentation and Antigen Processing Ubiquitination Proteosome Degradation and Innate Immune Response**	*BTRC*	beta-transducin repeat containing E3 ubiquitin protein ligase	N/A	2.16	N/A	N/A	N/A	N/A
	ITCH	itchy E3 ubiquitin protein ligase	N/A	2.96	N/A	N/A	N/A	N/A
	PSMA5	proteasome 20S subunit alpha 5	-2.35	-2.28	-2.27	N/A	N/A	-3.03
	PSMB3	proteasome 20S subunit beta 3	-2.35	-2.34	N/A	N/A	N/A	N/A
	PSMB5	proteasome 20S subunit beta 5	-3.12	N/A	N/A	N/A	N/A	N/A
	PSMB7	proteasome 20S subunit beta 7	-2.25	-3.2	N/A	N/A	N/A	N/A
	PSMD1	proteasome 26S subunit, non-ATPase 1	-2.19	-3.17	N/A	N/A	N/A	N/A
	PSMD11	proteasome 26S subunit, non-ATPase 11	N/A	-2.51	N/A	N/A	N/A	N/A

	PSMD2	proteasome 26S subunit ubiquitin receptor, non-ATPase 2	N/A	-2.79	N/A	N/A	N/A	N/A
	PSMD8	proteasome 26S subunit, non-ATPase 8	-2.51	N/A	N/A	N/A	N/A	N/A
	PSME2	proteasome activator subunit 2	N/A	-3.4	N/A	N/A	N/A	N/A
	SKP1	S-phase kinase associated protein 1	N/A	-2.63	N/A	N/A	N/A	N/A
	UBR4	ubiquitin protein ligase E3 component n-recognin 4	N/A	2.1	N/A	N/A	N/A	N/A
**Class I MHC Mediated Antigen Processing & Presentation and Antigen Processing Ubiquitination Proteosome Degradation and Innate Immune Response and Adaptive Immune Response**	*CUL1*	cullin 1	N/A	-2.02	N/A	N/A	N/A	N/A
	FBXW11	F-box and WD repeat domain containing 11	-2.27	N/A	N/A	N/A	N/A	-2.13
	PSMA7	proteasome 20S subunit alpha 7	N/A	-3.63	N/A	N/A	N/A	N/A
	PSMB2	proteasome 20S subunit beta 2	-3.03	N/A	N/A	N/A	N/A	-2.23
	PSMB8	proteasome 20S subunit beta 8	-3.56	N/A	N/A	N/A	N/A	3.1
	PSMC3	proteasome 26S subunit, ATPase 3	N/A	-3.58	N/A	N/A	N/A	N/A
	PSMC4	proteasome 26S subunit, ATPase 4	-4.42	-4.15	N/A	N/A	N/A	N/A
	PSMC5	proteasome 26S subunit, ATPase 5	N/A	-2.99	N/A	N/A	N/A	N/A
	PSMD12	proteasome 26S subunit, non-ATPase 12	-2.51	-2.45	-3.31	N/A	N/A	-4.51
	PSMD14	proteasome 26S subunit, non-ATPase 14	N/A	-3.99	N/A	N/A	N/A	N/A
	PSMD3	proteasome 26S subunit, non-ATPase 3	-2.15	-3.45	N/A	N/A	N/A	N/A
	PSME3	proteasome activator subunit 3	-2.85	-13.18	-5.73	N/A	N/A	-5.57
	UBE2N	ubiquitin conjugating enzyme E2 N	N/A	-3.21	-3.99	N/A	N/A	-2.12
**Class I MHC Mediated Antigen Processing & Presentation and Antigen Processing Ubiquitination Proteosome Degradation and Adaptive**	*ASB8*	ankyrin repeat and SOCS box containing 8	N/A	2.41	N/A	N/A	N/A	N/A
	CBLB	Cbl proto-oncogene B	N/A	4.71	N/A	N/A	N/A	N/A
	CCNF	cyclin F	N/A	N/A	N/A	N/A	N/A	-10.71
	FBXL14	F-box and leucine rich repeat protein 14	N/A	-2.58	-2.48	N/A	N/A	N/A
	FBXL20	F-box and leucine rich repeat protein 20	N/A	3.51	2.78	N/A	N/A	N/A
	FBXL5	F-box and leucine rich repeat protein 5	-2.59	N/A	N/A	N/A	N/A	N/A
	FBXO2	F-box protein 2	-2.92	N/A	N/A	N/A	N/A	N/A

**Immune Response**	*FBXW2*	F-box and WD repeat domain containing 2	N/A	-2.33	N/A	N/A	N/A	N/A
	GLMN	glomulin, FKBP associated protein	-3.42	N/A	N/A	N/A	N/A	N/A
	LMO7	LIM domain 7	N/A	3.4	N/A	N/A	N/A	N/A
	LRR1	leucine rich repeat protein 1	N/A	-2.6	N/A	N/A	N/A	-2.32
	LTN1	listerin E3 ubiquitin protein ligase 1	N/A	3.21	N/A	N/A	N/A	N/A
	PJA2	praja ring finger ubiquitin ligase 2	N/A	2.87	N/A	N/A	N/A	3.18
	RCHY1	ring finger and CHY zinc finger domain containing 1	-2.48	N/A	N/A	N/A	N/A	N/A
	RNF123	ring finger protein 123	-2.98	N/A	N/A	N/A	N/A	N/A
	RNF14	ring finger protein 14	N/A	-2.09	N/A	N/A	N/A	N/A
	RNF19A	ring finger protein 19A, RBR E3 ubiquitin protein ligase	N/A	3.4	N/A	N/A	N/A	N/A
	RNF19B	ring finger protein 19B	2.97	3.83	N/A	N/A	N/A	N/A
	RNF213	ring finger protein 213	2.98	4.81	N/A	N/A	N/A	2.85
	RNF220	ring finger protein 220	N/A	-5.67	N/A	N/A	N/A	N/A
	SMURF1	SMAD specific E3 ubiquitin protein ligase 1	N/A	4.54	N/A	N/A	N/A	N/A
	STUB1	STIP1 homology and U-box containing protein 1	N/A	-2.4	N/A	N/A	N/A	N/A
	TRAF7	TNF receptor associated factor 7	-2.5	-3.29	N/A	N/A	N/A	N/A
	UBA6	ubiquitin like modifier activating enzyme 6	3.93	N/A	N/A	N/A	N/A	N/A
	UBE2H	ubiquitin conjugating enzyme E2 H	N/A	N/A	N/A	N/A	N/A	4.95
	UBE2S	ubiquitin conjugating enzyme E2 S	N/A	N/A	N/A	N/A	N/A	-3.45
	UBR2	ubiquitin protein ligase E3 component n-recognin 2	N/A	2.22	N/A	N/A	N/A	N/A
	UFL1	UFM1 specific ligase 1	N/A	2.26	N/A	N/A	N/A	N/A
	WSB1	WD repeat and SOCS box containing 1	N/A	30.82	N/A	N/A	N/A	5.5
**Innate Immune Response**	*AAMP*	angio associated migratory cell protein	-2.22	N/A	N/A	N/A	N/A	N/A
	ACLY	ATP citrate lyase	-6.34	-2.59	N/A	N/A	N/A	N/A
	ACTB	actin beta	-2.36	N/A	N/A	N/A	N/A	N/A
	ADAM10	ADAM metallopeptidase domain 10	N/A	N/A	N/A	N/A	-3.19	N/A
	ADGRE5	adhesion G protein-coupled receptor E5	3.37	N/A	N/A	N/A	N/A	N/A

ADGRG3	adhesion G protein-coupled receptor G3	2.61	N/A	N/A	N/A	N/A	N/A
AGL	amylo-alpha-1, 6-glucosidase, 4-alpha-glucanotransferase	-3.99	-4.21	N/A	N/A	N/A	N/A
AHCYL1	adenosylhomocysteinase like 1	-3.08	N/A	N/A	N/A	-2.34	N/A
ALAD	aminolevulinate dehydratase	-2.42	N/A	N/A	N/A	N/A	N/A
ALDH3B1	aldehyde dehydrogenase 3 family member B1	-3.23	N/A	N/A	N/A	-2.83	N/A
ALDOA	aldolase, fructose-bisphosphate A	N/A	3.72	N/A	N/A	N/A	N/A
ALDOC	aldolase, fructose-bisphosphate C	N/A	102.52	N/A	N/A	6.9	N/A
ALOX5	arachidonate 5-lipoxygenase	N/A	N/A	N/A	N/A	-2.06	N/A
ANO6	anoctamin 6	N/A	3.88	N/A	N/A	N/A	N/A
ANPEP	alanyl aminopeptidase, membrane	N/A	N/A	N/A	N/A	6.03	N/A
ANXA2	annexin A2	N/A	2.39	N/A	N/A	N/A	N/A
APAF1	apoptotic peptidase activating factor 1	N/A	8.62	N/A	N/A	N/A	N/A
APEH	acylaminoacyl-peptide hydrolase	-3.41	-4.64	N/A	N/A	N/A	N/A
APRT	adenine phosphoribosyltransferase	-3.64	-3.82	N/A	N/A	N/A	N/A
ARL8A	ADP ribosylation factor like GTPase 8A	N/A	2.91	N/A	N/A	N/A	N/A
ARPC1B	actin related protein 2/3 complex subunit 1B	-3.29	N/A	N/A	N/A	N/A	N/A
ARPC3	actin related protein 2/3 complex subunit 3	N/A	2.71	N/A	N/A	N/A	N/A
ARPC5	actin related protein 2/3 complex subunit 5	-2.02	N/A	N/A	N/A	N/A	N/A
ARSB	arylsulfatase B	-2.17	-4.03	N/A	N/A	N/A	N/A
ASAH1	N-acylsphingosine amidohydrolase 1	N/A	N/A	N/A	N/A	-2.2	N/A
ATF2	activating transcription factor 2	N/A	2.9	N/A	N/A	N/A	N/A
ATP6V0A1	ATPase H+ transporting V0 subunit a1	-5.02	N/A	N/A	N/A	N/A	N/A
ATP6V0A2	ATPase H+ transporting V0 subunit a2	N/A	-5.69	N/A	N/A	-4.23	N/A
ATP6V0B	ATPase H+ transporting V0 subunit b	-3.68	N/A	N/A	N/A	N/A	N/A
ATP6V0D1	ATPase H+ transporting V0 subunit d1	-2.73	-3.83	N/A	N/A	N/A	N/A
ATP6V1A	ATPase H+ transporting V1 subunit A	N/A	2.24	N/A	N/A	-2.04	N/A
ATP6V1B1	ATPase H+ transporting V1 subunit B1	-2.57	N/A	N/A	N/A	N/A	N/A

ATP6V1C1	ATPase H+ transporting V1 subunit C1	N/A	2.98	N/A	N/A	N/A	N/A
ATP6V1G1	ATPase H+ transporting V1 subunit G1	N/A	N/A	N/A	N/A	-2.29	N/A
ATP6V1H	ATPase H+ transporting V1 subunit H	-2.28	N/A	N/A	N/A	N/A	N/A
BAIAP2	BAR/IMD domain containing adaptor protein 2	-3.4	N/A	N/A	N/A	N/A	N/A
BCL10	BCL10 immune signaling adaptor	N/A	2.29	N/A	N/A	N/A	N/A
BCL2	BCL2 apoptosis regulator	N/A	-15.81	N/A	N/A	N/A	N/A
BRI3	brain protein I3	N/A	2.07	N/A	N/A	N/A	N/A
BST1	bone marrow stromal cell antigen 1	N/A	N/A	N/A	N/A	4.32	N/A
BST2	bone marrow stromal cell antigen 2	59.63	N/A	N/A	N/A	N/A	N/A
C4A	complement C4A (Rodgers blood group)	-2.97	N/A	N/A	N/A	N/A	N/A
C4B	complement C4B (Chido blood group)	-2.97	N/A	N/A	N/A	N/A	N/A
C5	complement C5	N/A	-2.55	N/A	N/A	N/A	N/A
C5AR1	complement C5a receptor 1	-2.47	N/A	N/A	N/A	N/A	N/A
C5AR2	complement C5a receptor 2	N/A	-2.34	N/A	N/A	N/A	N/A
CASP10	caspase 10	-2.44	N/A	N/A	N/A	N/A	N/A
CASP2	caspase 2	N/A	-3.9	N/A	N/A	N/A	N/A
CAT	catalase	N/A	2.43	N/A	N/A	N/A	N/A
CCT2	chaperonin containing TCP1 subunit 2	N/A	-2.86	N/A	N/A	N/A	N/A
CCT8	chaperonin containing TCP1 subunit 8	N/A	-2.25	N/A	N/A	N/A	N/A
CD44	CD44 molecule (Indian blood group)	N/A	N/A	N/A	N/A	3.49	N/A
CD55	CD55 molecule (Cromer blood group)	N/A	5.68	N/A	N/A	N/A	N/A
CD68	CD68 molecule	13.29	32.62	N/A	N/A	N/A	N/A
CEACAM6	CEA cell adhesion molecule 6	N/A	3.92	N/A	N/A	N/A	N/A
CEP290	centrosomal protein 290	N/A	3.03	N/A	N/A	N/A	N/A
TIMP2	TIMP metallopeptidase inhibitor 2	N/A	13.67	N/A	N/A	N/A	N/A
CFH	complement factor H	-5.58	N/A	N/A	N/A	N/A	N/A
CFI	complement factor I	-14.78	N/A	N/A	N/A	N/A	N/A

CFL1	cofilin 1	N/A	-2.38	N/A	N/A	N/A	N/A
CHI3L1	chitinase 3 like 1	-140.93	N/A	N/A	N/A	N/A	N/A
CLU	clusterin	-5.32	N/A	N/A	N/A	N/A	N/A
CMTM6	CKLF like MARVEL transmembrane domain containing 6	N/A	-2.49	N/A	N/A	N/A	N/A
CNN2	calponin 2	N/A	4.77	N/A	N/A	N/A	N/A
CNPY3	canopy FGF signaling regulator 3	N/A	-2.92	N/A	N/A	N/A	N/A
COMMD3	COMM domain containing 3	-2.62	-2.88	N/A	N/A	N/A	N/A
COPB1	COPI coat complex subunit beta 1	N/A	3.86	N/A	N/A	N/A	N/A
CPNE3	copine 3	N/A	2.68	N/A	N/A	N/A	N/A
CRCP	CGRP receptor component	-2.74	N/A	N/A	N/A	N/A	N/A
CREG1	cellular repressor of E1A stimulated genes 1	-6.43	N/A	N/A	N/A	N/A	N/A
CTNNB1	catenin beta 1	-2.46	N/A	N/A	N/A	N/A	N/A
CXCL1	C-X-C motif chemokine ligand 1	N/A	N/A	N/A	N/A	-3.42	N/A
CYLD	CYLD lysine 63 deubiquitinase	2.37	N/A	N/A	N/A	N/A	N/A
CYSTM1	cysteine rich transmembrane module containing 1	-2.82	N/A	N/A	N/A	-2.75	N/A
DBNL	drebrin like	N/A	-4.88	N/A	N/A	N/A	N/A
DDOST	dolichyl-diphosphooligosaccharide--protein glycosyltransferase non-catalytic subunit	-3.19	N/A	N/A	N/A	N/A	N/A
DDX3X	DEAD-box helicase 3 X-linked	-3.56	N/A	N/A	N/A	N/A	N/A
DDX58	DExD/H-box helicase 58	4.73	-4.02	N/A	N/A	N/A	N/A
DEGS1	delta 4-desaturase, sphingolipid 1	N/A	-9.53	N/A	N/A	N/A	N/A
DERA	deoxyribose-phosphate aldolase	N/A	N/A	N/A	N/A	3.24	N/A
DGAT1	diacylglycerol O-acyltransferase 1	-2.99	N/A	N/A	N/A	N/A	N/A
DHX36	DEAH-box helicase 36	N/A	N/A	N/A	N/A	-3.69	N/A
DHX9	DExH-box helicase 9	-3.17	-7.42	N/A	N/A	N/A	N/A
DIAPH1	diaphanous related formin 1	N/A	-2.87	N/A	N/A	N/A	N/A

DOCK1	dedicator of cytokinesis 1	N/A	-2.02	N/A	N/A	N/A	N/A
DOCK2	dedicator of cytokinesis 2	N/A	N/A	N/A	N/A	6.6	N/A
DSP	desmoplakin	N/A	2.41	N/A	N/A	N/A	N/A
DTX4	deltex E3 ubiquitin ligase 4	-3.43	N/A	N/A	N/A	N/A	N/A
DUSP4	dual specificity phosphatase 4	4.83	N/A	N/A	N/A	N/A	N/A
DYNC1H1	dynein cytoplasmic 1 heavy chain 1	N/A	2.5	N/A	N/A	N/A	N/A
DYNLT1	dynein light chain Tctex-type 1	N/A	N/A	N/A	N/A	3.68	N/A
EEA1	early endosome antigen 1	N/A	N/A	N/A	N/A	-4.11	N/A
EEF2	eukaryotic translation elongation factor 2	-3.11	N/A	N/A	N/A	N/A	N/A
ELMO1	engulfment and cell motility 1	N/A	N/A	N/A	N/A	2.08	N/A
ENPP4	ectonucleotide pyrophosphatase/phosphodiesterase 4	-2.07	2.7	N/A	N/A	3.8	N/A
FABP5	fatty acid binding protein 5	-4.86	N/A	N/A	N/A	3.01	N/A
FRK	fyn related Src family tyrosine kinase	N/A	-4	N/A	N/A	N/A	N/A
FTL	ferritin light chain	-2.15	7.12	N/A	N/A	-2.89	N/A
GAB2	GRB2 associated binding protein 2	-3.64	-2.16	N/A	N/A	N/A	N/A
GLIPR1	GLI pathogenesis related 1	N/A	N/A	N/A	N/A	65.38	N/A
GM2A	GM2 ganglioside activator	N/A	2.21	N/A	N/A	N/A	N/A
GPI	glucose-6-phosphate isomerase	N/A	2.63	N/A	N/A	N/A	N/A
GYG1	glycogenin 1	N/A	-3.6	N/A	N/A	N/A	N/A
HCK	HCK proto-oncogene, Src family tyrosine kinase	2.74	N/A	N/A	N/A	N/A	N/A
HMOX2	heme oxygenase 2	N/A	-2.75	N/A	N/A	N/A	N/A
HP	haptoglobin	-4.37	N/A	N/A	N/A	N/A	N/A
HSP90AA1	heat shock protein 90 alpha family class A member 1	-4.61	N/A	N/A	N/A	N/A	N/A
HSP90AB1	heat shock protein 90 alpha family class B member 1	-2.49	N/A	N/A	N/A	N/A	N/A
HSP90B1	heat shock protein 90 beta family member 1	N/A	4.6	N/A	N/A	N/A	N/A
HSPA1A	heat shock protein family A (Hsp70) member 1A	-4.4	N/A	N/A	N/A	N/A	N/A
HSPA1B	heat shock protein family A (Hsp70) member 1B	-3.52	N/A	N/A	N/A	N/A	N/A

	HSPA8	heat shock protein family A (Hsp70) member 8	-4.7	-2.13	N/A	N/A	N/A	N/A
	IDH1	isocitrate dehydrogenase (NADP(+)) 1	-10.09	N/A	N/A	N/A	-10.65	N/A
	IFIH1	interferon induced with helicase C domain 1	6.11	N/A	N/A	N/A	N/A	N/A
	IL1B	interleukin 1 beta	49.82	N/A	N/A	N/A	N/A	N/A
	ILF2	interleukin enhancer binding factor 2	-2.31	-2.64	N/A	N/A	N/A	N/A
	IMPDH1	inosine monophosphate dehydrogenase 1	N/A	-2.56	N/A	N/A	N/A	N/A
	IMPDH2	inosine monophosphate dehydrogenase 2	-2.4	N/A	N/A	N/A	N/A	N/A
	IQGAP1	IQ motif containing GTPase activating protein 1	N/A	3.25	N/A	N/A	N/A	N/A
	IQGAP2	IQ motif containing GTPase activating protein 2	-9.14	N/A	N/A	N/A	N/A	N/A
	IRAK1	interleukin 1 receptor associated kinase 1	N/A	-2.54	N/A	N/A	N/A	N/A
	IRAK2	interleukin 1 receptor associated kinase 2	3.11	N/A	N/A	N/A	N/A	N/A
	IRF7	interferon regulatory factor 7	2.37	N/A	N/A	N/A	N/A	N/A
	IST1	IST1 factor associated with ESCRT-III	N/A	2.17	N/A	N/A	N/A	N/A
	ITLN1	intelectin 1	-114.45	N/A	N/A	N/A	N/A	N/A
	JUN	Jun proto-oncogene, AP-1 transcription factor subunit	N/A	3.84	N/A	N/A	4.58	N/A
	KCMF1	potassium channel modulatory factor 1	2.17	N/A	N/A	N/A	N/A	N/A
	KCNAB2	potassium voltage-gated channel subfamily A regulatory beta subunit 2	-6.25	N/A	N/A	N/A	N/A	N/A
	KPNB1	karyopherin subunit beta 1	-2.06	N/A	N/A	N/A	N/A	N/A
	LAMTOR1	late endosomal/lysosomal adaptor, MAPK and MTOR activator 1	-3.13	-2.67	N/A	N/A	N/A	N/A
	LAMTOR3	late endosomal/lysosomal adaptor, MAPK and MTOR activator 3	N/A	2.07	N/A	N/A	N/A	N/A
	LCN2	lipocalin 2	-51.88	N/A	N/A	N/A	N/A	N/A
	LGALS3	galectin 3	2.32	N/A	N/A	N/A	-2.82	N/A
	LIMK1	LIM domain kinase 1	N/A	-2.32	N/A	N/A	N/A	N/A
	LPCAT1	lysophosphatidylcholine acyltransferase 1	N/A	4.52	N/A	N/A	5.21	N/A
	LRG1	leucine rich alpha-2-glycoprotein 1	N/A	-2.18	N/A	N/A	N/A	N/A

	LTA4H	leukotriene A4 hydrolase	N/A	2.77	N/A	N/A	N/A	N/A
	LYN	LYN proto-oncogene, Src family tyrosine kinase	N/A	5.97	N/A	N/A	N/A	N/A
	MANBA	mannosidase beta	N/A	5.87	N/A	N/A	N/A	N/A
	MAP2K1	mitogen-activated protein kinase kinase 1	N/A	2.91	N/A	N/A	N/A	N/A
	MAP2K6	mitogen-activated protein kinase kinase 6	N/A	N/A	N/A	N/A	-4.55	N/A
	MAPKAPK2	MAPK activated protein kinase 2	N/A	-2.58	N/A	N/A	N/A	N/A
	MAPKAPK3	MAPK activated protein kinase 3	N/A	-2.24	N/A	N/A	N/A	N/A
	MEF2A	myocyte enhancer factor 2A	3.95	2.21	N/A	N/A	N/A	N/A
	METTL7A	methyltransferase like 7A	N/A	-6.1	N/A	N/A	N/A	N/A
	MGST1	microsomal glutathione S-transferase 1	N/A	-4.93	N/A	N/A	N/A	N/A
	MIF	macrophage migration inhibitory factor	N/A	N/A	N/A	N/A	3.82	N/A
	MLEC	malectin	-2.29	N/A	N/A	N/A	N/A	N/A
	MOSPD2	motile sperm domain containing 2	N/A	2.44	N/A	N/A	N/A	N/A
	MUC1	mucin 1, cell surface associated	5.15	4.24	N/A	N/A	N/A	N/A
	MUCL1	mucin like 1	N/A	-6.63	N/A	N/A	N/A	N/A
	MVP	major vault protein	N/A	2.43	N/A	N/A	N/A	N/A
	MYH9	myosin heavy chain 9	N/A	N/A	N/A	N/A	3.27	N/A
	MYO10	myosin X	10.86	N/A	N/A	N/A	N/A	N/A
	NAPRT	nicotinate phosphoribosyltransferase	-3.65	N/A	N/A	N/A	N/A	N/A
	NDUFC2	NADH:ubiquinone oxidoreductase subunit C2	-3.69	-3.4	N/A	N/A	N/A	N/A
	NEU1	neuraminidase 1	N/A	5.36	N/A	N/A	N/A	N/A
	NF2	neurofibromin 2	N/A	-3.23	N/A	N/A	N/A	N/A
	NFASC	neurofascin	N/A	N/A	N/A	N/A	6.01	N/A
	NFKB2	nuclear factor kappa B subunit 2	2.33	2.68	N/A	N/A	N/A	N/A
	NHLRC3	NHL repeat containing 3	N/A	2.29	N/A	N/A	N/A	N/A
	NIT2	nitrilase family member 2	N/A	-2.3	N/A	N/A	N/A	N/A
	NLRP1	NLR family pyrin domain containing 1	3.43	N/A	N/A	N/A	N/A	N/A

NLRP3	NLR family pyrin domain containing 3	2.58	N/A	N/A	N/A	N/A	N/A
NME2	NME/NM23 nucleoside diphosphate kinase 2	-2.63	N/A	N/A	N/A	N/A	N/A
NPC2	NPC intracellular cholesterol transporter 2	N/A	6.85	N/A	N/A	N/A	N/A
OLR1	oxidized low density lipoprotein receptor 1	-5.78	N/A	N/A	N/A	N/A	N/A
ORMDL3	ORMDL sphingolipid biosynthesis regulator 3	N/A	2.3	N/A	N/A	N/A	N/A
P2RX7	purinergic receptor P2X 7	5.18	N/A	N/A	N/A	N/A	N/A
PA2G4	proliferation-associated 2G4	-2.13	N/A	N/A	N/A	N/A	N/A
PADI2	peptidyl arginine deiminase 2	N/A	-3.8	N/A	N/A	3.38	N/A
PAK3	p21 (RAC1) activated kinase 3	N/A	N/A	N/A	N/A	3.71	N/A
PANX1	pannexin 1	2.85	-3.03	N/A	N/A	N/A	N/A
PDZD11	PDZ domain containing 11	N/A	N/A	N/A	N/A	2.47	N/A
PELI2	pellino E3 ubiquitin protein ligase family member 2	N/A	N/A	N/A	N/A	-2.58	N/A
PGM1	phosphoglucomutase 1	N/A	5.31	N/A	N/A	N/A	N/A
PGM2	phosphoglucomutase 2	-4.1	N/A	N/A	N/A	N/A	N/A
PI3	peptidase inhibitor 3	-13.67	N/A	N/A	N/A	N/A	N/A
PIK3R1	phosphoinositide-3-kinase regulatory subunit 1	N/A	-7.33	N/A	N/A	N/A	N/A
PLAC8	placenta associated 8	N/A	N/A	N/A	N/A	7.17	N/A
PLAUR	plasminogen activator, urokinase receptor	N/A	N/A	N/A	N/A	5.55	N/A
PLCG2	phospholipase C gamma 2	N/A	N/A	N/A	N/A	-3.53	N/A
PLD1	phospholipase D1	5.45	2.95	N/A	N/A	N/A	N/A
PLPP4	phospholipid phosphatase 4	N/A	N/A	N/A	N/A	20.03	N/A
PNP	purine nucleoside phosphorylase	N/A	-3.66	N/A	N/A	N/A	N/A
POLR1C	RNA polymerase I and III subunit C	-2.5	-2.57	N/A	N/A	N/A	N/A
POLR2E	RNA polymerase II, I and III subunit E	-2.84	-2.59	N/A	N/A	N/A	N/A
POLR2H	RNA polymerase II, I and III subunit H	N/A	N/A	N/A	N/A	2.18	N/A
POLR2K	RNA polymerase II, I and III subunit K	-2.66	-2.38	N/A	N/A	N/A	N/A
POLR2L	RNA polymerase II, I and III subunit L	-4.08	-8.5	N/A	N/A	N/A	N/A

POLR3B	RNA polymerase III subunit B	-4.03	-4.86	N/A	N/A	-3.15	N/A
POLR3C	RNA polymerase III subunit C	N/A	-3.6	N/A	N/A	N/A	N/A
POLR3D	RNA polymerase III subunit D	N/A	3.76	N/A	N/A	N/A	N/A
POLR3E	RNA polymerase III subunit E	N/A	N/A	N/A	N/A	-3.04	N/A
POLR3F	RNA polymerase III subunit F	N/A	N/A	N/A	N/A	-2.1	N/A
POLR3G	RNA polymerase III subunit G	N/A	-3.16	N/A	N/A	N/A	N/A
POLR3H	RNA polymerase III subunit H	-2.7	-4.24	N/A	N/A	N/A	N/A
POLR3K	RNA polymerase III subunit K	N/A	-3.43	N/A	N/A	N/A	N/A
PPP2CA	protein phosphatase 2 catalytic subunit alpha	-2.09	-2.1	N/A	N/A	N/A	N/A
PPP2R5D	protein phosphatase 2 regulatory subunit B'delta	N/A	2.16	N/A	N/A	N/A	N/A
PPP3CA	protein phosphatase 3 catalytic subunit alpha	N/A	-3.16	N/A	N/A	N/A	N/A
PRCP	prolylcarboxypeptidase	N/A	2.86	N/A	N/A	N/A	N/A
PRDX4	peroxiredoxin 4	N/A	N/A	N/A	N/A	3.16	N/A
PRDX6	peroxiredoxin 6	-5.07	N/A	N/A	N/A	N/A	N/A
PRKCE	protein kinase C epsilon	N/A	-2.84	N/A	N/A	N/A	N/A
PRKDC	protein kinase, DNA-activated, catalytic subunit	-3.82	N/A	N/A	N/A	N/A	N/A
PRSS3	serine protease 3	N/A	N/A	N/A	N/A	6.46	N/A
PSAP	prosaposin	N/A	2.44	N/A	N/A	-2.48	N/A
PTAFR	platelet activating factor receptor	N/A	5.55	N/A	N/A	N/A	N/A
PTGES2	prostaglandin E synthase 2	-4.17	-4.38	N/A	N/A	N/A	N/A
PTK2	protein tyrosine kinase 2	N/A	2.54	N/A	N/A	N/A	N/A
PTPRJ	protein tyrosine phosphatase receptor type J	N/A	2.56	N/A	N/A	N/A	N/A
PYGL	glycogen phosphorylase L	N/A	28.02	N/A	N/A	-2.64	N/A
QPCT	glutaminyl-peptide cyclotransferase	2.85	-3.31	N/A	N/A	3.36	N/A
QSOX1	quiescin sulfhydryl oxidase 1	N/A	9.36	N/A	N/A	3.74	N/A
RAB31	RAB31, member RAS oncogene family	N/A	-3.68	N/A	N/A	N/A	N/A
RAB3A	RAB3A, member RAS oncogene family	N/A	3.73	N/A	N/A	N/A	N/A

RAB3D	RAB3D, member RAS oncogene family	N/A	N/A	N/A	N/A	-2.57	N/A
RAB5C	RAB5C, member RAS oncogene family	N/A	-3.39	N/A	N/A	N/A	N/A
RAC2	Rac family small GTPase 2	4.09	N/A	N/A	N/A	N/A	N/A
RAP1A	RAP1A, member of RAS oncogene family	N/A	-2.01	N/A	N/A	N/A	N/A
RAP2C	RAP2C, member of RAS oncogene family	N/A	-4.75	N/A	N/A	N/A	N/A
RELB	RELB proto-oncogene, NF-kB subunit	2.89	N/A	N/A	N/A	N/A	N/A
RHOF	ras homolog family member F, filopodia associated	N/A	N/A	N/A	N/A	-8.3	N/A
RNASET2	ribonuclease T2	N/A	N/A	N/A	N/A	8.41	N/A
ROCK1	Rho associated coiled-coil containing protein kinase 1	N/A	N/A	N/A	N/A	-3.39	N/A
S100A7	S100 calcium binding protein A7	N/A	-5.65	N/A	N/A	N/A	N/A
S100A7A	S100 calcium binding protein A7A	N/A	-2.01	N/A	N/A	N/A	N/A
S100P	S100 calcium binding protein P	N/A	5.64	N/A	N/A	N/A	N/A
SAA1	serum amyloid A1	-45.93	N/A	N/A	N/A	N/A	N/A
SARM1	sterile alpha and TIR motif containing 1	-14.31	N/A	N/A	N/A	N/A	N/A
SCAMP1	secretory carrier membrane protein 1	2.42	N/A	N/A	N/A	N/A	N/A
SDCBP	syndecan binding protein	-2.45	7.3	N/A	N/A	N/A	N/A
SERPINA1	serpin family A member 1	N/A	N/A	N/A	N/A	5.65	N/A
SERPINB1	serpin family B member 1	2.62	N/A	N/A	N/A	N/A	N/A
SERPING1	serpin family G member 1	N/A	N/A	N/A	N/A	10.06	N/A
SFTPD	surfactant protein D	-2.12	N/A	N/A	N/A	N/A	N/A
SHC1	SHC adaptor protein 1	-3.05	N/A	N/A	N/A	N/A	N/A
SIRPA	signal regulatory protein alpha	N/A	N/A	N/A	N/A	4.8	N/A
SLC27A2	solute carrier family 27 member 2	N/A	-18.91	N/A	N/A	N/A	N/A
SLC2A3	solute carrier family 2 member 3	194.29	N/A	N/A	N/A	7.84	N/A
SLC44A2	solute carrier family 44 member 2	N/A	N/A	N/A	N/A	13.58	N/A
SLCO4C1	solute carrier organic anion transporter family member 4C1	N/A	N/A	N/A	N/A	5.21	N/A
STK10	serine/threonine kinase 10	N/A	-2.67	N/A	N/A	N/A	N/A

SURF4	surfeit 4	-2.34	N/A	N/A	N/A	N/A	N/A
SVIP	small VCP interacting protein	-2.54	N/A	N/A	N/A	N/A	N/A
TAB3	TGF-beta activated kinase 1 (MAP3K7) binding protein 3	N/A	3.34	N/A	N/A	N/A	N/A
TAX1BP1	Tax1 binding protein 1	-2.91	N/A	N/A	N/A	-6.03	N/A
TIFA	TRAF interacting protein with forkhead associated domain	5.19	N/A	N/A	N/A	N/A	N/A
TKFC	triokinase and FMN cyclase	N/A	-5.42	N/A	N/A	N/A	N/A
TLR6	toll like receptor 6	N/A	N/A	N/A	N/A	2.34	N/A
TMEM30A	transmembrane protein 30A	N/A	5.55	N/A	N/A	N/A	N/A
TNFAIP3	TNF alpha induced protein 3	N/A	4.66	N/A	N/A	N/A	N/A
TNFAIP6	TNF alpha induced protein 6	N/A	N/A	N/A	N/A	8.02	N/A
TOM1	target of myb1 membrane trafficking protein	N/A	3.09	N/A	N/A	N/A	N/A
TRAF6	TNF receptor associated factor 6	2.71	2.17	N/A	N/A	N/A	N/A
TRAPPC1	trafficking protein particle complex subunit 1	N/A	N/A	N/A	N/A	3.52	N/A
TSPAN14	tetraspanin 14	N/A	-3.29	N/A	N/A	N/A	N/A
TXN	thioredoxin	N/A	-3.27	N/A	N/A	N/A	N/A
TXNIP	thioredoxin interacting protein	5.42	N/A	N/A	N/A	N/A	N/A
UNC13D	unc-13 homolog D	3.39	N/A	N/A	N/A	N/A	N/A
UNC93B1	unc-93 homolog B1, TLR signaling regulator	N/A	2.3	N/A	N/A	N/A	N/A
VAPA	VAMP associated protein A	N/A	3.65	N/A	N/A	N/A	N/A
VAT1	vesicle amine transport 1	N/A	5.29	N/A	N/A	N/A	N/A
VCP	valosin containing protein	-2.15	N/A	N/A	N/A	N/A	N/A
VRK3	VRK serine/threonine kinase 3	N/A	2.65	N/A	N/A	2.13	N/A
VTN	vitronectin	-6.85	N/A	N/A	N/A	N/A	N/A
WASF2	WASP family member 2	2.89	6.77	N/A	N/A	N/A	N/A
WASL	WASP like actin nucleation promoting factor	N/A	3.53	N/A	N/A	N/A	N/A
XRCC5	X-ray repair cross complementing 5	-2.71	-2.32	N/A	N/A	N/A	N/A
XRCC6	X-ray repair cross complementing 6	N/A	-2.18	N/A	N/A	N/A	N/A

	YPEL5	yippee like 5	N/A	6.77	N/A	N/A	N/A	N/A
**Adaptive Immune Response**	*ACTR1A*	actin related protein 1A	N/A	-5.35	N/A	N/A	N/A	N/A
	AKT2	AKT serine/threonine kinase 2	-3.38	-3.11	N/A	-2.26	N/A	N/A
	AKT3	AKT serine/threonine kinase 3	2.09	N/A	N/A	N/A	2.34	N/A
	AP1S2	adaptor related protein complex 1 subunit sigma 2	N/A	-5.52	N/A	N/A	N/A	N/A
	AP2M1	adaptor related protein complex 2 subunit mu 1	N/A	N/A	N/A	-2.2	N/A	N/A
	CD274	CD274 molecule	3.7	N/A	N/A	N/A	2.32	N/A
	CD40	CD40 molecule	N/A	N/A	N/A	N/A	5.24	N/A
	CD74	CD74 molecule	3.1	N/A	N/A	-3.27	N/A	N/A
	CD99	CD99 molecule (Xg blood group)	2.11	N/A	N/A	N/A	N/A	N/A
	COL17A1	collagen type XVII alpha 1 chain	N/A	N/A	N/A	N/A	41.71	N/A
	COL1A1	collagen type I alpha 1 chain	4.98	N/A	N/A	N/A	11.04	N/A
	COLEC12	collectin subfamily member 12	N/A	N/A	N/A	N/A	2.72	N/A
	FBXO30	F-box protein 30	N/A	N/A	N/A	N/A	-3.47	N/A
	HLA-DMA	major histocompatibility complex, class II, DM alpha	N/A	4.06	N/A	N/A	10.68	N/A
	HLA-DPA1	major histocompatibility complex, class II, DP alpha 1	N/A	N/A	N/A	-2.93	2.24	N/A
	HLA-DPB1	major histocompatibility complex, class II, DP beta 1	N/A	N/A	N/A	-3.2	N/A	N/A
	HLA-DRA	major histocompatibility complex, class II, DR alpha	N/A	N/A	N/A	-20.2	N/A	N/A
	HLA-DRB1	major histocompatibility complex, class II, DR beta 1	N/A	2.49	N/A	-5.14	N/A	N/A
	KIF3A	kinesin family member 3A	N/A	N/A	N/A	N/A	-3.67	N/A
	KIR2DL4	killer cell immunoglobulin like receptor, two Ig domains and long cytoplasmic tail 4	N/A	N/A	N/A	2.3	N/A	N/A
	KLHL11	kelch like family member 11	N/A	N/A	N/A	N/A	-2.04	N/A
	KLHL5	kelch like family member 5	N/A	N/A	N/A	N/A	-2.73	N/A
	LRRC41	leucine rich repeat containing 41	N/A	N/A	N/A	N/A	44.81	N/A
	MAPKAP1	MAPK associated protein 1	-3.61	-5.64	N/A	N/A	N/A	N/A
	MTOR	mechanistic target of rapamycin kinase	N/A	-2.09	N/A	N/A	N/A	N/A
	NCR3LG1	natural killer cell cytotoxicity receptor 3 ligand 1	-3.53	N/A	N/A	-3.78	N/A	N/A

	NPDC1	neural proliferation, differentiation and control 1	N/A	-2.42	N/A	N/A	N/A	N/A
	ORAI1	ORAI calcium release-activated calcium modulator 1	N/A	-3.04	N/A	N/A	N/A	N/A
	PIK3R3	phosphoinositide-3-kinase regulatory subunit 3	N/A	-4.17	N/A	N/A	-4.05	N/A
	PPP2R5B	protein phosphatase 2 regulatory subunit B'beta	2.83	N/A	N/A	N/A	N/A	N/A
	PRR5	proline rich 5	-2.03	N/A	N/A	N/A	N/A	N/A
	PTEN	phosphatase and tensin homolog	N/A	2.42	N/A	N/A	N/A	N/A
	RACGAP1	Rac GTPase activating protein 1	N/A	N/A	N/A	N/A	6.07	N/A
	RICTOR	RPTOR independent companion of MTOR complex 2	N/A	4.26	N/A	N/A	-2.71	N/A
	SH2D1B	SH2 domain containing 1B	N/A	N/A	N/A	2.76	N/A	N/A
	SLAMF7	SLAM family member 7	69.71	N/A	N/A	N/A	N/A	N/A
	TRIB3	tribbles pseudokinase 3	N/A	N/A	N/A	-12.12	-13.71	N/A
	UBE2D1	ubiquitin conjugating enzyme E2 D1	N/A	N/A	N/A	2.82	N/A	N/A
	VCAM1	vascular cell adhesion molecule 1	N/A	N/A	N/A	-8.32	N/A	N/A
**Innate Immune Response and Adaptive Immune Response**	*AP1M1*	adaptor related protein complex 1 subunit mu 1	N/A	N/A	N/A	N/A	2.51	N/A
	CALM1	calmodulin 1	-2.39	-2.16	N/A	N/A	N/A	N/A
	CTSC	cathepsin C	-9.92	N/A	N/A	-4.31	N/A	N/A
	CTSH	cathepsin H	-7.85	N/A	N/A	N/A	N/A	N/A
	DNM1	dynamin 1	-3.49	N/A	N/A	N/A	N/A	N/A
	FYN	FYN proto-oncogene, Src family tyrosine kinase	N/A	2.75	N/A	N/A	N/A	N/A
	GRB2	growth factor receptor bound protein 2	-2.89	N/A	N/A	N/A	N/A	N/A
	HLA-A	major histocompatibility complex, class I, A	N/A	3.39	N/A	N/A	N/A	N/A
	HLA-B	major histocompatibility complex, class I, B	N/A	3.83	N/A	N/A	N/A	6.24
	HLA-C	major histocompatibility complex, class I, C	N/A	3.83	N/A	N/A	N/A	5.76
	ITPR1	inositol 1,4,5-trisphosphate receptor type 1	N/A	N/A	N/A	N/A	3.44	N/A
	ITPR3	inositol 1,4,5-trisphosphate receptor type 3	N/A	4.21	N/A	N/A	N/A	N/A
	MAP3K7	mitogen-activated protein kinase kinase kinase 7	N/A	N/A	N/A	N/A	-2.27	N/A
	MAP3K8	mitogen-activated protein kinase kinase kinase 8	N/A	3.19	N/A	N/A	N/A	N/A

	MYD88	MYD88 innate immune signal transduction adaptor	N/A	-4.51	N/A	N/A	N/A	N/A
	NCK1	NCK adaptor protein 1	N/A	2.36	N/A	N/A	-3.31	N/A
	NFKB1	nuclear factor kappa B subunit 1	N/A	-3.02	N/A	N/A	N/A	N/A
	PIK3R2; IFI30	phosphoinositide-3-kinase regulatory subunit 2; IFI30 lysosomal thiol reductase	N/A	-3.05	N/A	N/A	N/A	N/A
	PPP2R1A	protein phosphatase 2 scaffold subunit Aalpha	-3.56	N/A	N/A	N/A	N/A	N/A
	PPP2R1B	protein phosphatase 2 scaffold subunit Abeta	-2.97	N/A	N/A	N/A	N/A	N/A
	PRKACA	protein kinase cAMP-activated catalytic subunit alpha	N/A	-2.49	N/A	N/A	N/A	N/A
	PTPN11	protein tyrosine phosphatase non-receptor type 11	-3.42	N/A	N/A	N/A	N/A	N/A
	RIPK2	receptor interacting serine/threonine kinase 2	N/A	2.36	N/A	N/A	N/A	N/A
	SYK	spleen associated tyrosine kinase	N/A	-2.58	N/A	N/A	N/A	N/A
	TAB2	TGF-beta activated kinase 1 (MAP3K7) binding protein 2	N/A	3.21	N/A	N/A	N/A	N/A
	TUBB4B	tubulin beta 4B class Ivb	N/A	-3.34	N/A	N/A	3.58	N/A

Supplementary Table 13. Fold change of H226-specific immune-related genes based on pathway analysis

Pathways	Gene Symbol	Gene Description	Fold Change
**Hallmark Inflammatory Response**	*ADM*	adrenomedullin	4.25
	ATP2A2	ATPase, Ca++ transporting, cardiac muscle, slow twitch 2	-2.53
	C5AR1	complement component 5a receptor 1	-2.47
	CD82	CD82 molecule	-5.22
	IL1A	interleukin 1 alpha	11.42
	IL1B	interleukin 1 beta	49.82
	IL1R1	interleukin 1 receptor, type I	-28.29
	IL6	interleukin 6	3.36
	INHBA	inhibin beta A	47.42
	IRAK2	interleukin 1 receptor associated kinase 2	3.11
	ITGA5	integrin alpha 5	14.23
	ITGB3	integrin beta 3	3.67
	LDLR	low density lipoprotein receptor; microRNA 6886	-7.58
	NLRP3	NLR family, pyrin domain containing 3	2.58
	OLR1	oxidized low density lipoprotein (lectin-like) receptor 1	-5.78
	OSMR	oncostatin M receptor	2.27
	P2RX7	purinergic receptor P2X, ligand gated ion channel, 7	5.18
	PDPN	podoplanin	-2.12
	RNF144B	ring finger protein 144B	2.99
	SLC11A2	solute carrier family 11 (proton-coupled divalent metal ion transporter), member 2	-5.57
	SLC31A1	solute carrier family 31 (copper transporter), member 1	-4.19
	SLC4A4	solute carrier family 4 (sodium bicarbonate cotransporter), member 4	12.27
	TPBG	trophoblast glycoprotein	3.57
**Hallmark Interferon alpha Response**	*IFI44*	interferon-induced protein 44	7.84
	IFI44L	interferon-induced protein 44-like	38.49
	IFIH1	interferon induced, with helicase C domain 1	6.11
	IL7	interleukin 7	-2.67
	MOV10	Mov10 RISC complex RNA helicase	-3.66
	MVB12A	multivesicular body subunit 12A; BST2 interferon stimulated positive regulator (non-protein coding)	5.28
	NCOA7	nuclear receptor coactivator 7	-4.78
	TDRD7	tudor domain containing 7	3.33
	TRAFD1	TRAF-type zinc finger domain containing 1	-5.74
	TXNIP	thioredoxin interacting protein	5.42
**Hallmark Interferon gamma Response**	*CD274*	CD274 molecule	3.7
	CFH	complement factor H	-5.58

	PFKP	phosphofructokinase, platelet	5.89
	PSMB2	proteasome subunit beta 2	-3.03
	RNF213	ring finger protein 213	2.98
	SLAMF7	SLAM family member 7	69.71
	ST3GAL5	ST3 beta-galactoside alpha-2,3-sialyltransferase 5	-21.73
**Hallmark IL2-STAT5 Signaling**	*AHCY*	adenosylhomocysteinase	-2.64
	BHLHE40	basic helix-loop-helix family, member e40	5.52
	BMPR2	bone morphogenetic protein receptor type II	2.37
	CAPG	capping protein (actin filament), gelsolin-like	2.81
	CCND2	cyclin D2	-3.77
	CCND3	cyclin D3	-3.01
	CDC6	cell division cycle 6	-11.03
	CDCP1	CUB domain containing protein 1	18.9
	DCPS	decapping enzyme, scavenger	-2.75
	ENPP1	ectonucleotide pyrophosphatase/phosphodiesterase 1	-8.86
	F2RL2	coagulation factor II (thrombin) receptor-like 2	-3.69
	HK2	hexokinase 2	7.51
	IGF1R	insulin-like growth factor 1 receptor	-4.27
	IL2RA	interleukin 2 receptor, alpha	2.22
	ITIH5	inter-alpha-trypsin inhibitor heavy chain family, member 5	5.1
	LRIG1	leucine-rich repeats and immunoglobulin-like domains protein 1	2.75
	LRRC8C	leucine rich repeat containing 8 family, member C	2.65
	MUC1	mucin 1, cell surface associated	5.15
	MYO1E	myosin IE	4.22
	NDRG1	N-myc downstream regulated 1	28.54
	NFIL3	nuclear factor, interleukin 3 regulated	4.71
	ODC1	ornithine decarboxylase 1; small nucleolar RNA, H/ACA box 80B	-2.73
	P4HA1	prolyl 4-hydroxylase, alpha polypeptide I	11.56
	PLAGL1	pleiomorphic adenoma gene-like 1	3.19
	RNH1	ribonuclease/angiogenin inhibitor 1	-3
	RORA	RAR-related orphan receptor A	47.32
	SCN9A	sodium channel, voltage gated, type IX alpha subunit	10.04
	SLC1A5	solute carrier family 1 (neutral amino acid transporter), member 5	-3.42
	SLC2A3	solute carrier family 2 (facilitated glucose transporter), member 3	194.29
	SLC39A8	solute carrier family 39 (zinc transporter), member 8	-7.68
	SOCS2	suppressor of cytokine signaling 2	2.64
	TGM2	transglutaminase 2	11.05
	TRAF1	TNF receptor-associated factor 1	2.96

	UMPS	uridine monophosphate synthetase	-2.54
	WLS	wntless Wnt ligand secretion mediator	-7.78
**Reactome Interferon Signaling**	*CAMK2A*	calcium/calmodulin-dependent protein kinase II alpha	-2.48
	EIF4G2	eukaryotic translation initiation factor 4 gamma, 2	-2.38
	KPNB1	karyopherin (importin) beta 1	-2.06
	NDC1	NDC1 transmembrane nucleoporin	-2.57
	NUP188	nucleoporin 188kDa	-3.05
	NUP210	nucleoporin 210kDa	-12.77
	NUP43	nucleoporin 43kDa	-3.32
	NUP88	nucleoporin 88kDa	-2.25
	PDE12	phosphodiesterase 12	-2.5
	PPM1B	protein phosphatase, Mg2+/Mn2+ dependent, 1B	-2.06
	RAE1	ribonucleic acid export 1	-2.11
	SEH1L	SEH1-like nucleoporin	-2.37
	TRIM29	tripartite motif containing 29	6.15
	USP41	ubiquitin specific peptidase 41	2.27
**Reactome Interferon Signaling and Hallmark Interferon gamma Response**	*DDX58*	DEAD (Asp-Glu-Ala-Asp) box polypeptide 58	4.73
	EIF4E3	eukaryotic translation initiation factor 4E family member 3	2.45
	PML	promyelocytic leukemia	3.39
**Reactome Interferon Signaling and Hallmark IL2-STAT5 Signaling**	*IFNGR1*	interferon gamma receptor 1	-7.17
**Reactome Interferon Signaling and Hallmark Inflammatory Response**	*IFNGR2*	interferon gamma receptor 2	3.04
**Reactome Interferon Signaling and Hallmark Interferon alpha Response**	*TRIM26*	tripartite motif containing 26	-3.03
**Reactome Interferon Signaling and Reactome Interferon alpha/beta signaling**	*ABCE1*	ATP binding cassette subfamily E member 1	-4.46
	IFI6	interferon, alpha-inducible protein 6	19.34
	IFIT1	interferon-induced protein with tetratricopeptide repeats 1	23.54
	PTPN11	protein tyrosine phosphatase, non-receptor type 11	-3.42
**Reactome Interferon Signaling and Reactome Interferon alpha/beta Signaling and Hallmark Interferon gamma Response**	*OAS2*	2-5-oligoadenylate synthetase 2	42.5
	OAS3	2-5-oligoadenylate synthetase 3	4.67
	SAMHD1	SAM domain and HD domain 1	3.7
	SOCS3	suppressor of cytokine signaling 3	-2.08
**Reactome Interferon Signaling and Reactome Interferon alpha/beta Signaling and Hallmark Interferon gamma Response and Hallmark Interferon alpha Response**	*MX1*	MX dynamin-like GTPase 1	14.38
	PSMB8	proteasome subunit beta 8	-3.56
	RSAD2	radical S-adenosyl methionine domain containing 2	9.88
	IFI27	interferon, alpha-inducible protein 27	15.8
	IFIT3	interferon-induced protein with tetratricopeptide repeats 3	4.59

	USP18	ubiquitin specific peptidase 18	2.03
**Reactome Interferon Signaling and Reactome Interferon alpha/beta Signaling and Hallmark Inflammatory Response and Hallmark Interferon alpha Response**	*IRF7*	interferon regulatory factor 7	2.37
**Hallmark Interferon gamma Response and Hallmark IL2-STAT5 Signaling**	*CASP3*	caspase 3	-3.73
**Hallmark Interferon gamma Response and Hallmark Interferon alpha Response**	*CD74*	CD74 molecule, major histocompatibility complex, class II invariant chain	3.1
	CMPK2	cytidine monophosphate (UMP-CMP) kinase 2, mitochondrial	2.93
	EPSTI1	epithelial stromal interaction 1	10.16
	PARP12	poly(ADP-ribose) polymerase family member 12	4.24
	PARP14	poly(ADP-ribose) polymerase family member 14	4.1
**Hallmark Interferon gamma Response and Hallmark Interferon alpha Response and Hallmark IL2-STAT5 Signaling**	*PLSCR1*	phospholipid scramblase 1	6.97
**Hallmark Inflammatory Response and Hallmark Interferon gamma Response**	*HIF1A*	hypoxia inducible factor 1, alpha subunit	-3.27
**Hallmark Inflammatory Response and Hallmark IL2-STAT5 Signaling**	*KLF6*	Kruppel-like factor 6	7.76
	LIF	leukemia inhibitory factor	-3.11
	TNFSF10	tumor necrosis factor superfamily, member 10	11.15
**Hallmark Inflammatory Response and Hallmark Interferon alpha Response**	*CCRL2*	chemokine (C-C motif) receptor-like 2	5.44
	IL15	interleukin 15	2.67

Supplementary Table 14. Fold change of MCF-7-specific immune-related genes based on pathway analysis

Pathways	Gene Symbol	Gene Description	Fold Change
**Reactome Antigen Processing-Cross Presentation**	*B2M*	beta-2-microglobulin	2.85
	CALR	calreticulin	2.17
	CTSV	cathepsin V	5.51
	HLA-A	major histocompatibility complex, class I, A	3.39
	HLA-B	major histocompatibility complex, class I, B	3.83
	HLA-C	major histocompatibility complex, class I, C	3.83
	S100A8	S100 calcium binding protein A8	-3.93
	SEC22B	SEC22 homolog B, vesicle trafficking protein (gene/pseudogene)	2.14
	SEC61B	Sec61 translocon beta subunit	-5.33
	SEC61G	Sec61 translocon gamma subunit	2.76
	STX4	syntaxin 4	2.17
	TAP1	transporter 1, ATP-binding cassette, sub-family B (MDR/TAP)	2.96
	VAMP3	vesicle associated membrane protein 3	2.12
**Reactome Interleukin-1 Signaling**	*BTRC*	beta-transducin repeat containing E3 ubiquitin protein ligase	2.16
	CUL1	cullin 1	-2.02
	IRAK1	interleukin 1 receptor associated kinase 1; microRNA 718	-2.54
	MAP2K1	mitogen-activated protein kinase kinase 1	2.91
	MAP3K8	mitogen-activated protein kinase kinase kinase 8	3.19
	NFKB1	nuclear factor of kappa light polypeptide gene enhancer in B-cells 1	-3.02
	NFKB2	nuclear factor of kappa light polypeptide gene enhancer in B-cells 2 (p49/p100)	2.68
	RIPK2	receptor-interacting serine-threonine kinase 2	2.36
	SKP1	S-phase kinase-associated protein 1	-2.63
	TAB2	TGF-beta activated kinase 1/MAP3K7 binding protein 2	3.21
	TAB3	TGF-beta activated kinase 1/MAP3K7 binding protein 3	3.34
	TRAF6	TNF receptor-associated factor 6, E3 ubiquitin protein ligase	2.17
	UBE2N	ubiquitin conjugating enzyme E2N	-3.21
	MYD88	myeloid differentiation primary response 88	-4.51
**Reactome Antigen Processing-Cross Presentation and Reactome Interleukin-1 Signaling**	*PSMA5*	proteasome subunit alpha 5	-2.28
	PSMA7	proteasome subunit alpha 7	-3.63
	PSMB3	proteasome subunit beta 3	-2.34
	PSMB7	proteasome subunit beta 7	-3.2
	PSMC3	proteasome 26S subunit, ATPase 3	-3.58
	PSMC4	proteasome 26S subunit, ATPase 4	-4.15

	PSMC4	proteasome 26S subunit, ATPase 4	-4.03
	PSMC5	proteasome 26S subunit, ATPase 5	-2.99
	PSMD1	proteasome 26S subunit, non-ATPase 1	-3.17
	PSMD11	proteasome 26S subunit, non-ATPase 11	-2.51
	PSMD14	proteasome 26S subunit, non-ATPase 14	-3.99
	PSMD2	proteasome 26S subunit, non-ATPase 2	-2.79
	PSMD3	proteasome 26S subunit, non-ATPase 3	-3.45
	PSME2	proteasome activator subunit 2; microRNA 7703	-3.4
	PSME3	proteasome activator subunit 3	-13.18
